# Fundamental limits of repeaterless quantum communications

**DOI:** 10.1038/ncomms15043

**Published:** 2017-04-26

**Authors:** Stefano Pirandola, Riccardo Laurenza, Carlo Ottaviani, Leonardo Banchi

**Affiliations:** 1 Department of Computer Science and York Centre for Quantum Technologies, University of York, York YO10 5GH, UK; 2 Department of Physics and Astronomy, University College London, Gower Street, London WC1E 6BT, UK

## Abstract

Quantum communications promises reliable transmission of quantum information, efficient distribution of entanglement and generation of completely secure keys. For all these tasks, we need to determine the optimal point-to-point rates that are achievable by two remote parties at the ends of a quantum channel, without restrictions on their local operations and classical communication, which can be unlimited and two-way. These two-way assisted capacities represent the ultimate rates that are reachable without quantum repeaters. Here, by constructing an upper bound based on the relative entropy of entanglement and devising a dimension-independent technique dubbed ‘teleportation stretching', we establish these capacities for many fundamental channels, namely bosonic lossy channels, quantum-limited amplifiers, dephasing and erasure channels in arbitrary dimension. In particular, we exactly determine the fundamental rate-loss tradeoff affecting any protocol of quantum key distribution. Our findings set the limits of point-to-point quantum communications and provide precise and general benchmarks for quantum repeaters.

## Abstract (web summary)

Quantum communications will be used to transmit entanglement and secure keys, but it is important to estimate their optimal transfer rates. Here the authors compute the fundamental limit of repeaterless quantum communications for the most relevant practical scenario.

Quantum information^1^,^2^,^3^ is evolving towards next-generation quantum technologies, such as the realization of completely secure quantum communications^4^,^5^,^6^ and the long-term construction of a quantum Internet^7^,^8^,^9^,^10^. But quantum information is more fragile than its classical counterpart, so that the ideal performances of quantum protocols may rapidly degrade in realistic practical implementations. In particular, this is a basic limitation that affects any point-to-point protocol of quantum communication over a quantum channel, where two remote parties transmit qubits, distribute entanglement or secret keys. In this communication context, it is a crucial open problem to determine the ultimate rates achievable by the remote parties, assuming that they may apply arbitrary local operations (LOs) assisted by unlimited two-way classical communication (CCs), that we may briefly call adaptive LOCCs. The maximum rates achievable by these adaptive protocols are known as two-way (assisted) capacities of a quantum channel and represent fundamental benchmarks for quantum repeaters^11^.

Before our work, a single two-way capacity was known, discovered about 20 years ago^12^. For the important case of bosonic channels^2^, there were only partial results. Building on previous ideas^13^, ref. 14 introduced the reverse coherent information. By exploiting this notion and other tools^15^,^16^, the authors of ref. 17 established lower bounds for the two-way capacities of a Gaussian channel. This inspired a subsequent work^18^, which exploited the notion of squashed entanglement^19^ to build upper bounds; unfortunately the latter were too large to close the gap with the best-known lower bounds.

Our work addresses this basic problem. We devise a general methodology that completely simplifies the study of adaptive protocols and allows us to upperbound the two-way capacities of an arbitrary quantum channel with a computable single-letter quantity. In this way, we are able to establish exact formulas for the two-way capacities of several fundamental channels, such as bosonic lossy channels, quantum-limited amplifiers, dephasing and erasure channels in arbitrary dimension. For these channels, we determine the ultimate rates for transmitting quantum information (two-way quantum capacity *Q*_2_), distributing entanglement (two-way entanglement distribution capacity *D*_2_) and generating secret keys (secret-key capacity *K*). In particular, we establish the exact rate-loss scaling that restricts any point-to-point protocol of quantum key distribution (QKD) when implemented through a lossy communication line, such as an optical fibre or a free-space link.

## Results

### General overview of the results

As already mentioned in the Introduction, we establish the two-way capacities (*Q*_2_, *D*_2_ and *K*) for a number of quantum channels at both finite and infinite dimension, that is, we consider channels defined on both discrete variable (DV) and continuous variable (CV) systems^2^. Two-way capacities are benchmarks for quantum repeaters because they are derived by removing any technical limitation from the point-to-point protocols between the remote parties, who may perform the most general strategies allowed by quantum mechanics in the absence of pre-shared entanglement. Clearly, these ultimate limits cannot be achieved by imposing restrictions on the number of channel uses or enforcing energy constraints at the input. The relaxation of such constraints has also practical reasons since it approximates the working regime of current QKD protocols, that exploit large data blocks and high-energy Gaussian modulations^13^,^20^.

To achieve our results we suitably combine the relative entropy of entanglement (REE)^21^,^22^,^23^ with teleportation^9^,^24^,^25^,^26^ to design a general reduction method, which remarkably simplifies the study of adaptive protocols and two-way capacities. The first step is to show that the two-way capacities of a quantum channel cannot exceed a general bound based on the REE. The second step is the application of a technique, dubbed ‘teleportation stretching', which is valid for any channel at any dimension. This allows us to reduce any adaptive protocol into a block form, so that the general REE bound becomes a single-letter quantity. In this way, we easily upperbound the two-way capacities of any quantum channel, with closed formulas proven for bosonic Gaussian channels^2^, Pauli channels, erasure channels and amplitude damping channels^1^.

Most importantly, by showing coincidence with suitable lower bounds, we prove simple formulas for the two-way quantum capacity *Q*_2_ (=*D*_2_) and the secret-key capacity *K* of several fundamental channels. In fact, for the erasure channel we show that *K*=1−*p* where *p* is the erasure probability (only its *Q*_2_ was previously known^12^); for the dephasing channel we show that *Q*_2_=*K*=1−*H*_2_(*p*), where *H*_2_ is the binary Shannon entropy and *p* is the dephasing probability (these results for qubits are extended to any finite dimension). Then, for a quantum-limited amplifier, we show that 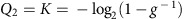 where *g* is the gain. Finally, for the lossy channel, we prove that 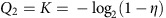 where *η* is the transmissivity. In particular, the secret-key capacity of the lossy channel is the maximum rate achievable by any optical implementation of QKD. At long distance, that is, high loss *η*≃0, we find the optimal rate-loss scaling of *K*≃1.44 *η* secret bits per channel use, a fundamental bound that only quantum repeaters may surpass.

In the following, we start by giving the main definitions. Then we formulate our reduction method and we derive the analytical results for the various quantum channels.

### Adaptive protocols and two-way capacities

**Figure 1:**
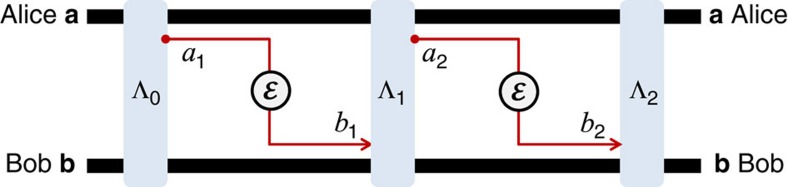
Adaptive quantum protocol. The first step is the preparation of the initial separable state 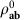 of **a** and **b** by some adaptive LOCC Λ_0_. After the preparation of the local registers, there is the first transmission: Alice picks a system from her local register *a*_1_∈**a**, so that the register is updated as **a**→**a***a*_1_; system *a*_1_ is sent through the channel 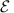, with Bob getting the output *b*_1_; Bob includes the output in his local register, which is updated as *b*_1_**b**→**b**; finally, Alice and Bob apply another adaptive LOCC Λ_1_ to their registers **a**,**b**. In the second transmission, Alice picks and sends another system *a*_2_∈**a** through channel 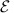 with output *b*_2_ for Bob. The parties apply a further adaptive LOCC Λ_2_ to their registers and so on. This procedure is repeated *n* times, with output state 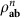 for the Alice's and Bob's local registers.

Suppose that Alice and Bob are separated by a quantum channel 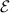 and want to implement the most general protocol assisted by adaptive LOCCs. This protocol may be stated for an arbitrary quantum task and then specified for the transmission of quantum information, distribution of entanglement or secret correlations. Assume that Alice and Bob have countable sets of systems, **a** and **b**, respectively. These are local registers which are updated before and after each transmission. The steps of an arbitrary adaptive protocol are described in Fig. 1.

After *n* transmissions, Alice and Bob share an output state 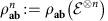 depending on the sequence of adaptive LOCCs 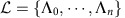. By definition, this adaptive protocol has a rate equal to *R*_*n*_ if the output 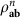 is sufficiently close to a target state *φ*_*n*_ with *nR*_*n*_ bits, that is, we may write 
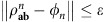
 in trace norm. The rate of the protocol is an average quantity, which means that the sequence 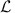 is assumed to be averaged over local measurements, so that it becomes trace-preserving. Thus, by taking the asymptotic limit in *n* and optimizing over 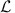, we define the generic two-way capacity of the channel as


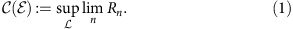


In particular, if the aim of the protocol is entanglement distribution, then the target state *φ*_*n*_ is a maximally entangled state and 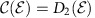. Because an ebit can teleport a qubit and a qubit can distribute an ebit, 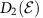 coincides with the two-way quantum capacity 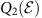. If the goal is to implement QKD, then the target state *φ*_*n*_ is a private state^27^ and 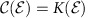. Here the secret-key capacity satisfies 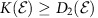, because ebits are specific types of secret bits and LOCCs are equivalent to LOs and public communication^27^. Thus, the generic two-way capacity 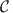 can be any of *D*_2_, *Q*_2_ or *K*, and these capacities must satisfy *D*_2_=*Q*_2_⩽*K*. Also, note that we may consider the two-way private capacity 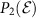, which is the maximum rate at which classical messages can be securely transmitted^15^. Because of the unlimited two-way CCs and the one-time pad, we have 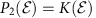, so that this equivalence is implicitly assumed hereafter.

### General bounds for two-way capacities

Let us design suitable bounds for 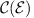. From below we know that we may use the coherent^28^,^29^ or reverse coherent^14^,^17^ information. Take a maximally entangled state of two systems *A* and *B*, that is, an Einstein–Podolsky–Rosen (EPR) state Φ_*AB*_. Propagating the *B*-part through the channel defines its Choi matrix 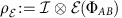. This allows us to introduce the coherent information of the channel 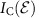 and its reverse counterpart 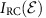, defined as 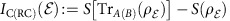, where *S*(·) is the von Neumann entropy. These quantities represent lower bounds for the entanglement that is distillable from the Choi matrix 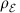 via one-way CCs, denoted as 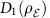. In other words, we can write the hashing inequality^16^


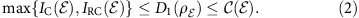


For bosonic systems, the ideal EPR state has infinite-energy, so that the Choi matrix of a bosonic channel is energy-unbounded (see Methods for notions on bosonic systems). In this case we consider a sequence of two-mode squeezed vacuum (TMSV) states^2^ Φ^*μ*^ with variance *μ*=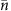
+1/2, where 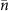 is the mean number of thermal photons in each mode. This sequence defines the bosonic EPR state as 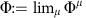. At the output of the channel, we have the sequence of quasi-Choi matrices


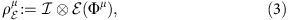


defining the asymptotic Choi matrix 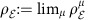. As a result, the coherent information quantities must be computed as limits on 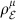 and the hashing inequality needs to be suitably extended (see Supplementary Note 2, which exploits the truncation tools of Supplementary Note 1).

In this work the crucial tool is the upper bound. Recall that, for any bipartite state *ρ*, the REE is defined as 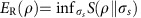, where *σ*_*s*_ is an arbitrary separable state and 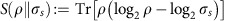 is the relative entropy^23^. Hereafter we extend this definition to include asymptotic (energy-unbounded) states. For an asymptotic state 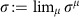 defined by a sequence of states *σ*^*μ*^, we define its REE as


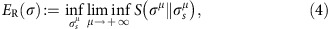


where 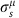 is an arbitrary sequence of separable states such that 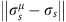
→0 for some separable *σ*_*s*_. In general, we also consider the regularized REE


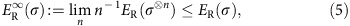


where 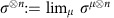 for an asymptotic state *σ*.

Thus, the REE of a Choi matrix 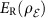 is correctly defined for channels of any dimension, both finite and infinite. We may also define the channel's REE as


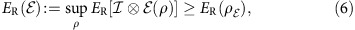


where the supremum includes asymptotic states for bosonic channels. In the following, we prove that these single-letter quantities, 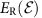 and 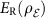, bound the two-way capacity 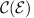 of basic channels. The first step is the following general result.

*Theorem* 1 (*general weak converse*): At any dimension, finite or infinite, the generic two-way capacity of a quantum channel 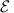 is upper bounded by the REE bound


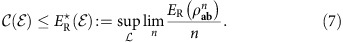


In Supplementary Note 3, we provide various equivalent proofs. The simplest one assumes an exponential growth of the shield system in the target private state^27^ as proven by ref. 30 and trivially adapted to CVs. Another proof is completely independent from the shield system. Once established the bound 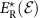, our next step is to simplify it by applying the technique of teleportation stretching, which is in turn based on a suitable simulation of quantum channels.

### Simulation of quantum channels

The idea of simulating channels by teleportation was first developed^31^,^32^ for Pauli channels^33^, and further studied in finite dimension^34^,^35^,^36^ after the introduction of generalized teleportation protocols^37^. Then, ref. 38 moved the first steps in the simulation of Gaussian channels via the CV teleportation protocol^25^,^26^. Another type of simulation is a deterministic version^39^ of a programmable quantum gate array^40^. Developed for DV systems, this is based on joint quantum operations, therefore failing to catch the LOCC structure of quantum communication. Here not only we fully extend the teleportation-simulation to CV systems, but we also design the most general channel simulation in a communication scenario; this is based on arbitrary LOCCs and may involve systems of any dimension, finite or infinite (see Supplementary Note 8 for comparisons and advances).

**Figure 2:**
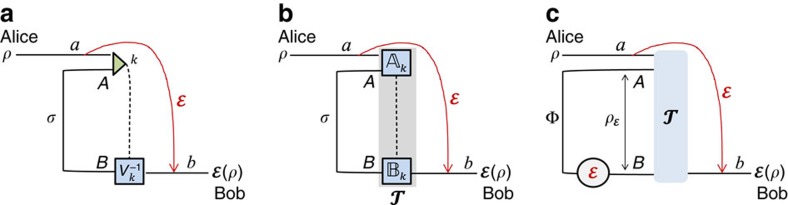
From teleportation- to LOCC-simulation of quantum channels. (**a**) Consider the generalized teleportation of an input state *ρ* of a *d*-dimensional system *a* by using a resource state *σ* of two systems, *A* and *B*, with corresponding dimensions *d* and *d*′ (finite or infinite). Systems *a* and *A* are subject to a Bell detection (triangle) with random outcome *k*. This outcome is associated with a projection onto a maximally entangled state up to an associated teleportation unitary *U*_*k*_ which is a Pauli operator for *d*<+∞ and a phase-displacement for *d*=+∞ (see Methods for the basics of quantum teleportation and the characterization of the teleportation unitaries). The classical outcome *k* is communicated to Bob, who applies a correction unitary 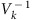 to his system *B* with output *b*. In general, *V*_*k*_ does not necessarily belong to the set {*U*_*k*_}. On average, this teleportation LOCC defines a teleportation channel 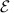 from *a* to *b*. It is clear that this construction also teleports part *a* of an input state involving ancillary systems. (**b**) In general we may replace the teleportation LOCC (Bell detection and unitary corrections) with an arbitrary LOCC 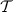: Alice performs a quantum operation 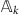 on her systems *a* and *A*, communicates the classical variable *k* to Bob, who then applies another quantum operation 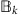 on his system *B*. By averaging over the variable *k*, so that 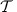 is certainly trace-preserving, we achieve the simulation 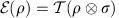 for any input state *ρ*. We say that a channel 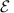 is ‘*σ*-stretchable' if it can be simulated by a resource state *σ* for some LOCC 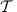. Note that Alice's and Bob's LOs 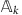 and 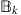 are arbitrary quantum operations; they may involve other local ancillas and also have extra labels (due to additional local measurements), in which case 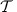 is assumed to be averaged over all these labels. (**c**) The most important case is when channel 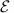 can be simulated by a trace-preserving LOCC 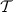 applied to its Choi matrix 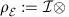 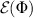, with Φ being an EPR state. In this case, we say that the channel is ‘Choi-stretchable'. These definitions are suitably extended to bosonic channels.

As explained in Fig. 2a, performing a teleportation LOCC (that is, Bell detection and unitary corrections) over a mixed state *σ* is a way to simulate a (certain type of) quantum channel 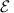 from Alice to Bob. However, more generally, the channel simulation can be realized using an arbitrary trace-preserving LOCC 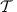 and an arbitrary resource state *σ* (see Fig. 2b). Thus, at any dimension, we say that a channel 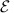 is ‘*σ*-stretchable' or ‘stretchable into *σ*' if there is a trace-preserving LOCC 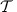 such that


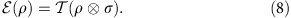


In general, we can simulate the same channel 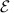 with different choices of 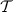 and *σ*. In fact, any channel is stretchable into some state *σ*: A trivial choice is decomposing 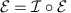, inserting 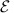 in Alice's LO and simulating 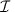 with teleportation over the ideal EPR state *σ*=Φ. Therefore, among all simulations, one needs to identify the best resource state that optimizes the functional under study. In our work, the best results are achieved when the state *σ* can be chosen as the Choi matrix of the channel. This is not a property of any channel but defines a class. Thus, we define ‘Choi-stretchable' a channel that can be LOCC-simulated over its Choi matrix, so that we may write equation (8) with 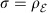 (see also Fig. 2c).

In infinite dimension, the LOCC simulation may involve limits 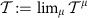 and 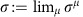 of sequences 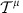 and *σ*^*μ*^. For any finite *μ*, the simulation 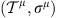 provides some teleportation channel 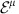. Now, suppose that an asymptotic channel 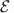 is defined as a pointwise limit of the sequence 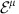, that is, we have 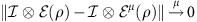 for any bipartite state *ρ*. Then, we say that 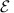 is stretchable with asymptotic simulation 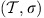. This is important for bosonic channels, for which Choi-based simulations can only be asymptotic and based on sequences 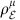.

### Teleportation covariance

We now discuss a property which easily identifies Choi-stretchable channels. Call 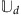 the random unitaries which are generated by the Bell detection in a teleportation process. For a qudit, 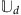 is composed of generalized Pauli operators, that is, the generators of the Weyl–Heisenberg group. For a CV system, the set 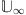 is composed of displacement operators^9^, spanning the infinite dimensional version of the previous group. In arbitrary dimension (finite or infinite), we say that a quantum channel is ‘teleportation-covariant' if, for any teleportation unitary 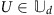, we may write


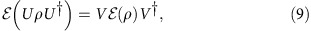


for some another unitary *V* (not necessarily in 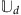).

The key property of a teleportation-covariant channel is that the input teleportation unitaries can be pushed out of the channel, where they become other correctable unitaries. Because of this property, the transmission of a system through the channel can be simulated by a generalized teleportation protocol over its Choi matrix. This is the content of the following proposition.

*Proposition 2* (*tele-covariance*): At any dimension, a teleportation-covariant channel 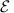 is Choi-stretchable. The simulation is a teleportation LOCC over its Choi matrix 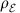, which is asymptotic for a bosonic channel.

**Figure 3:**
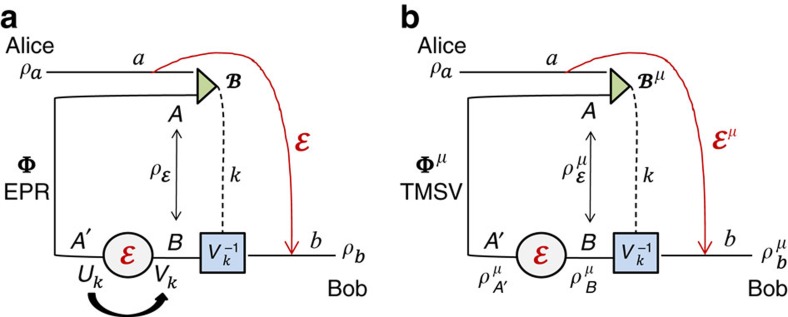
Teleportation-covariant channels are Choi-stretchable. (**a**) Consider the teleportation of an input state *ρ*_*a*_ by using an EPR state Φ_*AA*′_ of systems *A* and *A*′. The Bell detection 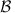 on systems *a* and *A* teleports the input state onto *A*′, up to a random teleportation unitary, that is, *ρ*_*A*′_=*U*_*k*_*ρ*_*a*_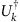. Because 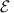 is teleportation-covariant, *U*_*k*_ is mapped into an output unitary *V*_*k*_ and we may write 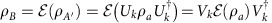. Therefore, Bob just needs to receive the outcome *k* and apply 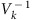, so that 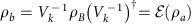. Globally, the process describes the simulation of channel 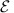 by means of a generalized teleportation protocol over the Choi matrix 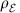. (**b**) The procedure is also valid for CV systems. If the input *a* is a bosonic mode, we need to consider finite-energy versions for the EPR state Φ and the Bell detection 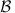, that is, we use a TMSV state Φ^*μ*^ and a corresponding quasi-projection 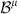 onto displaced TMSV states. At finite energy *μ*, the teleportation process from *a* to *A*′ is imperfect with some output 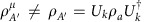. However, for any *ɛ*>0 and input state *ρ*_*a*_, there is a sufficiently large value of *μ* such that 
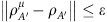
 (refs 25, 26). Consider the transmitted state 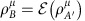. Because the trace distance decreases under channels, we have 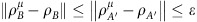. After the application of the correction unitary 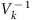, we have the output state 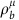 which satisfies 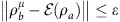. Taking the asymptotic limit of large *μ*, we achieve 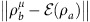
→0 for any input *ρ*_*a*_, therefore achieving the perfect asymptotic simulation of the channel. The asymptotic teleportation-LOCC is therefore 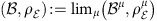 where 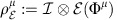. The result is trivially extended to the presence of ancillas.

The simple proof is explained in Fig. 3. The class of teleportation-covariant channels is wide and includes bosonic Gaussian channels, Pauli and erasure channels at any dimension (see Methods for a more detailed classification). All these fundamental channels are therefore Choi-stretchable. There are channels that are not (or not known to be) Choi-stretchable but still have decompositions 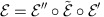 where the middle part 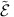 is Choi-stretchable. In this case, 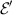 and 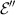 can be made part of Alice's and Bob's LOs, so that channel 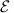 can be stretched into the state 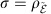. An example is the amplitude damping channel as we will see afterwards.

### Teleportation stretching of adaptive protocols

**Figure 4:**
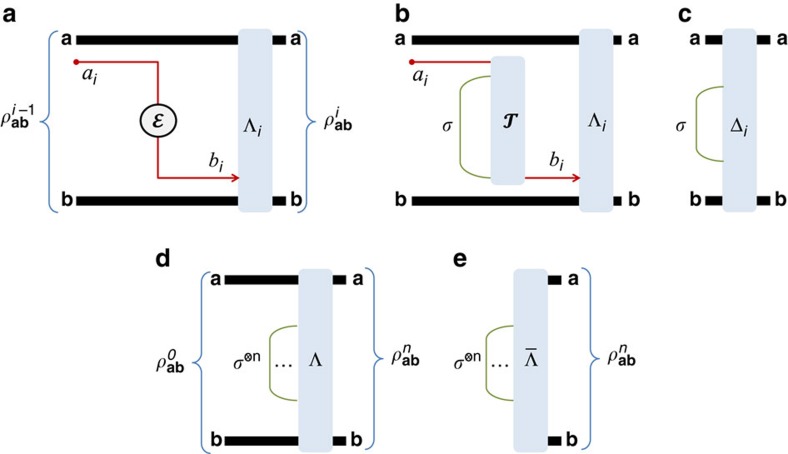
Teleportation stretching of an adaptive quantum protocol. (**a**) Consider the *i*th transmission through channel 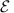, where the input (*i*−1)th register state is given by 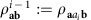. After transmission through 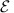 and the adaptive LOCC Λ_*i*_, the register state is updated to 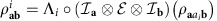. (**b**) Let us simulate the channel 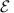 by a LOCC 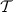 and a resource state *σ*. (**c**) The simulation LOCC 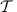 can be combined with the adaptive LOCC Λ_*i*_ into a single ‘extended' LOCC Δ_*i*_ while the resource state *σ* can be stretched back in time and out of the adaptive operations. We may therefore write 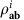
=Δ_*i*_(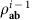
⊗*σ*). (**d**) We iterate the previous steps for all transmissions, so as to stretch *n* copies *σ*^⊗*n*^ and collapse all the extended LOCCs Δ_*n*_ o …o Δ_1_ into a single LOCC Λ. In other words, we may write 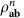
=Λ(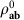
⊗*σ*^⊗*n*^). (**e**) Finally, we include the preparation of the separable state 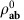 into Λ and we also average over all local measurements present in Λ, so that we may write the output state as 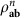
=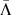
(*σ*^⊗*n*^) for a trace-preserving LOCC 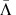. The procedure is asymptotic in the presence of asymptotic channel simulations (bosonic channels).

We are now ready to describe the reduction of arbitrary adaptive protocols. The procedure is schematically shown in Fig. 4. We start by considering the *i*th transmission through the channel 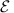, so that Alice and Bob's register state is updated from 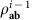 to 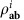. By using a simulation 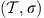, we show the input–output formula


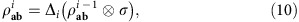


for some ‘extended' LOCC Δ_*i*_ (Fig. 4c). By iterating the previous formula *n* times, we may write the output state 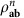
=Λ(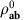
⊗*σ*^⊗*n*^) for 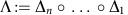 (as in Fig. 4d). Because the initial state 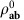 is separable, its preparation can be included in Λ and we may directly write 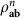
=Λ(*σ*^⊗*n*^). Finally, we average over all local measurements present in Λ, so that 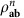
=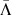
(*σ*^⊗*n*^) for a trace-preserving LOCC 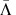 (Fig. 4e). More precisely, for any sequence of outcomes **u** with probability *p*(**u**), there is a conditional LOCC Λ_**u**_ with output 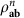
(**u**)=*p*(**u**)^−1^Λ_**u**_(*σ*^⊗*n*^). Thus, the mean output state 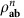 is generated by 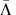
=∑_**u**_Λ_**u**_ (see Methods for more technical details on this LOCC averaging).

Note that the simulation of a bosonic channel 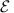 is typically asymptotic, with infinite-energy limits 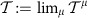 and 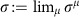. In this case, we repeat the procedure for some *μ*, with output 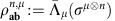, where 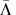
_*μ*_ is derived assuming the finite-energy LOCCs 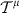. Then, we take the limit for large *μ*, so that 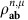 converges to 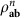 in trace norm (see Methods for details on teleportation stretching with bosonic channels). Thus, at any dimension, we have proven the following result.

*Lemma* 3 *(Stretching)*: Consider arbitrary n transmissions through a channel 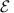 which is stretchable into a resource state σ. The output of an adaptive protocol can be decomposed into the block form


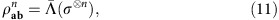


for some trace-preserving LOCC 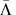. If the channel 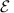 is Choi-stretchable, then we may write


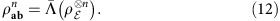


In particular, 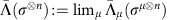 for an asymptotic channel simulation 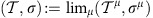.

According to this Lemma, teleportation stretching reduces an adaptive protocol performing an arbitrary task (quantum communication, entanglement distribution or key generation) into an equivalent block protocol, whose output state 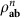 is the same but suitably decomposed as in equation (11) for any number *n* of channel uses. In particular, for Choi-stretchable channels, the output is decomposed into a tensor product of Choi matrices. An essential feature that makes the technique applicable to many contexts is the fact that the adaptive-to-block reduction maintains task and output of the original protocol so that, for example, adaptive key generation is reduced to block key generation and not entanglement distillation.

*Remark* 4: Some aspects of our method might be traced back to a precursory but very specific argument discussed in Section V of ref. 31, where protocols of quantum communication (through Pauli channels) were transformed into protocols of entanglement distillation (the idea was developed for one-way CCs, with an implicit extension to two-way CCs). However, while this argument may be seen as precursory, it is certainly not developed at the level of generality of the present work where the adaptive-to-block reduction is explicitly proven for any type of protocol and any channel at any dimension (see Supplementary Notes 9 and 10 for remarks on previous literature).

### REE as a single-letter converse bound

The combination of Theorem 1 and Lemma 3 provides the insight of our entire reduction method. In fact, let us compute the REE of the output state 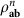, decomposed as in equation (11). Using the monotonicity of the REE under trace-preserving LOCCs, we derive


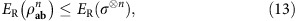


where the complicated 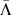 is fully discarded. Then, by replacing equation (13) into equation (7), we can ignore the supremum in the definition of 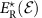 and get the simple bound


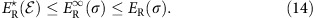


Thus, we can state the following main result.

*Theorem* 5 (*one-shot REE bound*): Let us stretch an arbitrary quantum channel 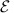 into some resource state *σ*, according to equation (8). Then, we may write


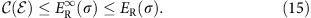


In particular, if 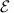 is Choi-stretchable, we have


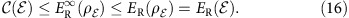


**Figure 5:**
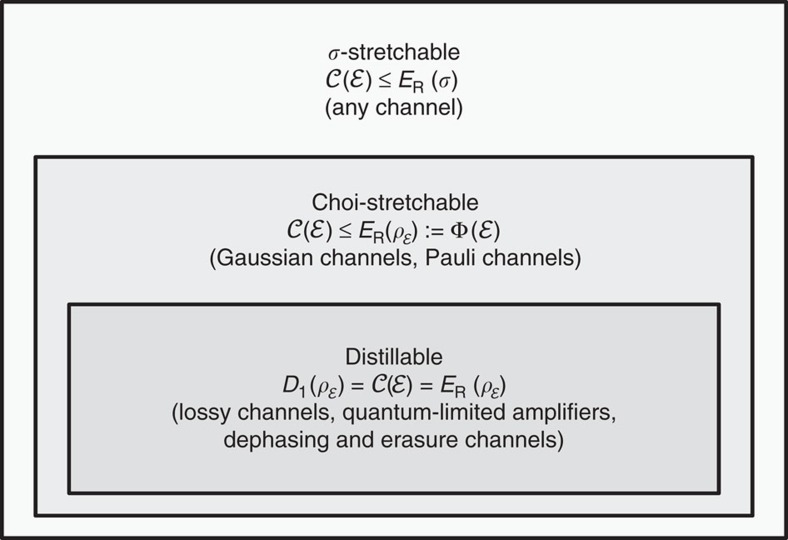
Classification of channels in DVs and CVs. We depict the classes of channels that are considered in this work, together with the bounds for their two-way capacities.

See Methods for a detailed proof, with explicit derivations for bosonic channels. We have therefore reached our goal and found single-letter bounds. In particular, note that 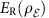 measures the entanglement distributed by a single EPR state, so that we may call it the ‘entanglement flux' of the channel 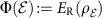. Remarkably, there is a sub-class of Choi-stretchable channels for which 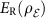 coincides with the lower bound 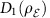 in equation (2). We call these ‘distillable channels'. We establish all their two-way capacities as 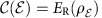. They include lossy channels, quantum-limited amplifiers, dephasing and erasure channels. See also Fig. 5.

### Immediate generalizations

Consider a fading channel, described by an ensemble 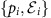, where channel 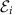 occurs with probability *p*_*i*_. Let us stretch 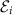 into a resource state *σ*_*i*_. For large *n*, we may decompose the output of an adaptive protocol as 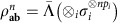, so that the two-way capacity of this channel is bounded by


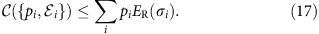


Then consider adaptive protocols of two-way quantum communication, where the parties have forward (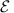) and backward 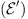 channels. The capacity 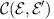 maximizes the number of target bits per channel use. Stretching 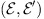 into a pair of states (*σ*, *σ*′), we find 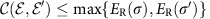. For Choi-stretchable channels, this means 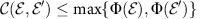, which reduces to 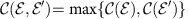 if they are distillable. In the latter case, the optimal strategy is using the channel with the maximum capacity (see Methods).

Yet another scenario is the multiband channel 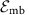, where Alice exploits *m* independent channels or ‘bands' 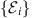, so that the capacity 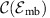 maximizes the number of target bits per multiband transmission. By stretching the bands 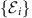 into resource states {*σ*_*i*_}, we find 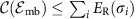. For Choi-stretchable bands, this means 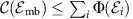, giving the additive capacity 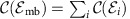 if they are distillable (see Methods).

### Ultimate limits of bosonic communications

We now apply our method to derive the ultimate rates for quantum and secure communication through bosonic Gaussian channels. These channels are Choi-stretchable with an asymptotic simulation involving 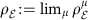. From equations (4) and (16), we may write


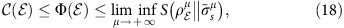


for a suitable converging sequence of separable states 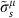.

For Gaussian channels, the sequences in equation (18) involve Gaussian states, for which we easily compute the relative entropy. In fact, for any two Gaussian states, *ρ*_1_ and *ρ*_2_, we prove the general formula *S*(*ρ*_1_||*ρ*_2_)=Σ(*V*_1_, *V*_2_)−*S*(*ρ*_1_), where Σ is a simple functional of their statistical moments (see Methods). After technical derivations (Supplementary Note 4), we then bound the two-way capacities of all Gaussian channels, starting from the most important, the lossy channel.

### Fundamental rate-loss scaling

Optical communications through free-space links or telecom fibres are inevitably lossy and the standard model to describe this scenario is the lossy channel. This is a bosonic Gaussian channel characterized by a transmissivity parameter *η*, which quantifies the fraction of input photons surviving the channel. It can be represented as a beam splitter mixing the signals with a zero-temperature environment (background thermal noise is negligible at optical and telecom frequencies).

For a lossy channel 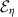 with arbitrary transmissivity *η* we apply our reduction method and compute the entanglement flux 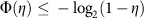. This coincides with the reverse coherent information of this channel *I*_RC_(*η*), first derived in ref. 17. Thus, we find that this channel is distillable and all its two-way capacities are given by


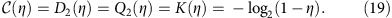


Interestingly, this capacity coincides with the maximum discord^41^ that can be distributed, since we may write^42^ *I*_RC_(*η*)=*D*(*B*|*A*), where the latter is the discord of the (asymptotic) Gaussian Choi matrix 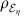 (ref. 43). We also prove the strict separation *Q*_2_(*η*)>*Q*(*η*), where *Q* is the unassisted quantum capacity^28^,^29^.

**Figure 6:**
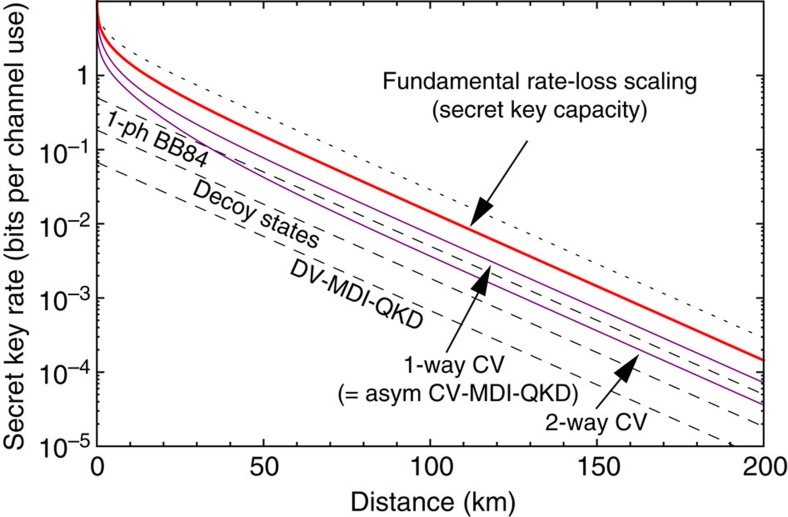
Ideal performances in QKD. We plot the secret-key rate (bits per channel use) versus Alice–Bob's distance (km) at the loss rate of 0.2 dB per km. The secret-key capacity of the channel (red line) sets the fundamental rate limit for point-to-point QKD in the presence of loss. Compare this capacity with a previous non-achievable upperbound^18^ (dotted line). We then show the maximum rates that are potentially achievable by current protocols, assuming infinitely long keys and ideal conditions, such as unit detector efficiencies, zero dark count rates, zero intrinsic error, unit error correction efficiency, zero excess noise (for CVs) and large modulation (for CVs). In the figure, we see that ideal implementations of CV protocols (purple lines) are not so far from the ultimate limit. In particular, we consider: (i) One-way no-switching protocol^63^, coinciding with CV-MDI-QKD^20^,^64^ in the most asymmetric configuration (relay approaching Alice^65^). For high loss 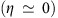, the rate scales as *η*/ln 4, which is just 1/2 of the capacity. Same scaling for the one-way switching protocol of ref. 13; (ii) Two-way protocol with coherent states and homodyne detection^66^,^67^ which scales as 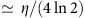 for high loss (thermal noise is needed for two-way to beat one-way QKD^66^). For the DV protocols (dashed lines), we consider: BB84 with single-photon sources^4^ with rate *η*/2; BB84 with weak coherent pulses and decoy states^6^ with rate *η*/(2*e*); and DV-MDI-QKD^68^,^69^ with rate *η*/(2*e*^2^). See Supplementary Note 6 for details on these ideal rates.

Expanding equation (19) at high loss 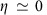, we find


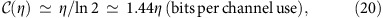


or about *η* nats per channel use. This completely characterizes the fundamental rate-loss scaling which rules long-distance quantum optical communications in the absence of quantum repeaters. It is important to remark that our work also proves the achievability of this scaling. This is a major advance with respect to existing literature, where previous studies with the squashed entanglement^18^ only identified a non-achievable upper bound. In Fig. 6, we compare the scaling of equation (20) with the maximum rates achievable by current QKD protocols.

The capacity in equation (19) is also valid for two-way quantum communication with lossy channels, assuming that *η* is the maximum transmissivity between the forward and feedback channels. It can also be extended to a multiband lossy channel, for which we write 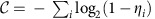, where *η*_*i*_ are the transmissivities of the various bands or frequency components. For instance, for a multimode telecom fibre with constant transmissivity *η* and bandwidth *W*, we have


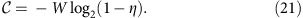


Finally, note that free-space satellite communications may be modelled as a fading lossy channel, that is, an ensemble of lossy channels 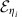 with associated probabilities *p*_*i*_ (ref. 44). In particular, slow fading can be associated with variations of satellite-Earth radial distance^45^,^46^. For a fading lossy channel 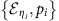, we may write


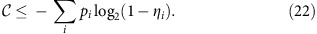


### Quantum communications with Gaussian noise

The fundamental limit of the lossy channel bounds the two-way capacities of all channels decomposable as 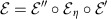 where 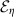 is a lossy component while 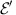 and 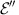 are extra channels. A channel 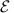 of this type is stretchable with resource state 
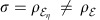
 and we may write 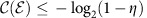. For Gaussian channels, such decompositions are known but we achieve tighter bounds if we directly stretch them using their own Choi matrix.

Let us start from the thermal-loss channel, which can be modelled as a beam splitter with transmissivity *η* in a thermal background with 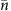 mean photons. Its action on input quadratures 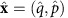 is given by 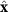
→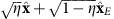 with *E* being a thermal mode. This channel is central for microwave communications^47^,^48^,^49^,^50^ but also important for CV QKD at optical and telecom frequencies, where Gaussian eavesdropping via entangling cloners results into a thermal-loss channel^2^.

For an arbitrary thermal-loss channel 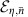 we apply our reduction method and compute the entanglement flux


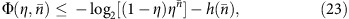


for 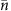
<*η*/(1−*η*), while zero otherwise. Here we set


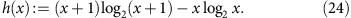


Combining this result with the lower bound given by the reverse coherent information^17^, we write the following inequalities for the two-way capacity of this channel


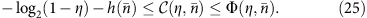


**Figure 7:**
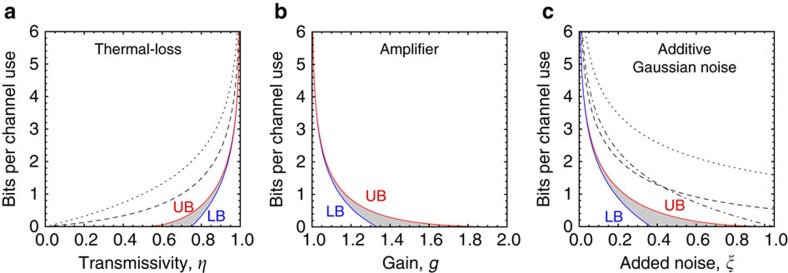
Two-way capacities for Gaussian channels in terms of the relevant channel parameters. (**a**) Two-way capacity 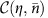 of the thermal-loss channel as a function of transmissivity *η* for 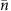
=1 thermal photon. It is contained in the shadowed area identified by the lower bound (LB) and upper bound (UB) of equation (25). Our upper bound is clearly tighter than those based on the squashed entanglement, computed in ref. 18 (dotted) and ref. 54 (dashed). Note that 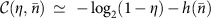 at high transmissivities. For 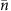
=0 (lossy channel) the shadowed region shrinks into a single line. (**b**) Two-way capacity 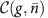 of the amplifier channel as a function of the gain *g* for 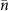
=1 thermal photon. It is contained in the shadowed specified by the bounds in equation (27). For small gains, we have 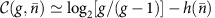. For 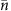
=0 (quantum-limited amplifier) the shadowed region shrinks into a single line. (**c**) Two-way capacity 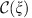 of the additive-noise Gaussian channel with added noise *ξ*. It is contained in the shadowed region specified by the bounds in equation (30). For small noise, we have 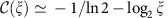. Our upper bound is much tighter than those of ref. 18 (dotted), ref. 54 (dashed) and ref. 51 (dot-dashed).

As shown in Fig. 7a, the two bounds tend to coincide at sufficiently high transmissivity. We clearly retrieve the previous result of the lossy channel for 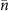
=0.

Another important Gaussian channel is the quantum amplifier. This channel 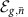 is described by 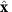
→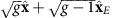, where *g*>1 is the gain and *E* is the thermal environment with 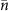 mean photons. We compute


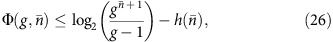


for 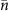
<(*g*−1)^−1^, while zero otherwise. Combining this result with the coherent information^51^, we get


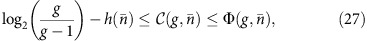


whose behaviour is plotted in Fig. 7b.

In the absence of thermal noise (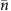
=0), the previous channel describes a quantum-limited amplifier 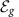, for which the bounds in equation (27) coincide. This channel is therefore distillable and its two-way capacities are


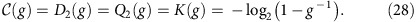


In particular, this proves that *Q*_2_(*g*) coincides with the unassisted quantum capacity *Q*(*g*)^51^,^52^. Note that a gain-2 amplifier can transmit at most 1 qubit per use.

Finally, one of the simplest models of bosonic decoherence is the additive-noise Gaussian channel^2^. This is the direct extension of the classical model of a Gaussian channel to the quantum regime. It can be seen as the action of a random Gaussian displacement over incoming states. In terms of input–output transformations, it is described by 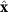
→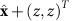 where *z* is a classical Gaussian variable with zero mean and variance *ξ*≥0. For this channel 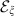 we find the entanglement flux


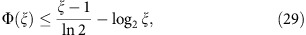


for *ξ*<1, while zero otherwise. Including the lower bound given by the coherent information^51^, we get


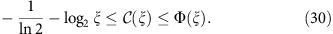


In Fig. 7c, see its behaviour and how the two bounds tend to rapidly coincide for small added noise.

### Ultimate limits in qubit communications

We now study the ultimate rates for quantum communication, entanglement distribution and secret-key generation through qubit channels, with generalizations to any finite dimension. For any DV channel 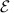 from dimension *d*_*A*_ to dimension *d*_*B*_, we may write the dimensionality bound 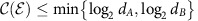. This is because we may always decompose the channel into 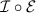 (or 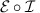), include 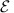 in Alice's (or Bob's) LOs and stretch the identity map into a Bell state with dimension *d*_*B*_ (or *d*_*A*_). For DV channels, we may also write the following simplified version of our Theorem 5 (see Methods for proof).

*Proposition* 6: For a Choi-stretchable channel 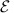 in finite dimension, we may write the chain


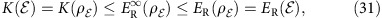


where 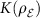 is the distillable key of 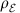.

In the following, we provide our results for DV channels, with technical details available in Supplementary Note 5.

### Pauli channels

A general error model for the transmission of qubits is the Pauli channel


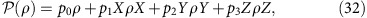


where *X*, *Y*, and *Z* are Pauli operators^1^ and **p**:={*p*_*k*_} is a probability distribution. It is easy to check that this channel is Choi-stretchable and its Choi matrix is Bell-diagonal. We compute its entanglement flux as


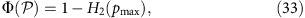


if *p*_max_:=max{*p*_*k*_}≥1/2, while zero otherwise. Since the channel is unital, we have that 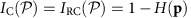, where *H* is the Shannon entropy. Thus, the two-way capacity of a Pauli channel satisfies


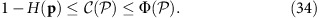


This can be easily generalized to arbitrary finite dimension (see Supplementary Note 5).

Consider the depolarising channel, which is a Pauli channel shrinking the Bloch sphere. With probability *p*, an input state becomes the maximally-mixed state


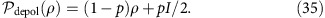


**Figure 8:**
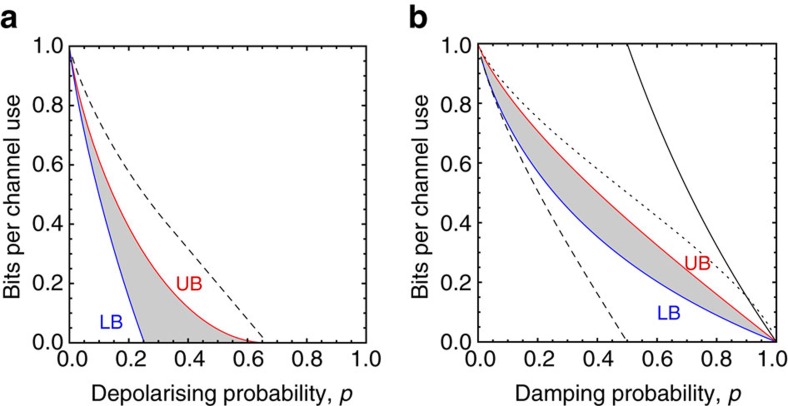
Two-way capacities of basic qubit channels. (**a**) Two-way capacity of the depolarizing channel 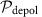 with arbitrary probability *p*. It is contained in the shadowed region specified by the bounds in equation (36). We also depict the best-known bound based on the squashed entanglement^54^ (dashed). (**b**) Two-way capacity of the amplitude damping channel 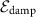 for arbitrary damping probability *p*. It is contained in the shadowed area identified by the lower bound (LB) of equation (48) and the upper bound (UB) of equation (49). We also depict the bound of equation (47) (upper solid line), which is good only at high dampings; and the bound 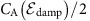 of ref. 54 (dotted line), which is computed from the entanglement-assisted classical capacity *C*_A_. Finally, note the separation of the two-way capacity 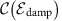 from the unassisted quantum capacity 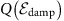 (dashed line).

Setting *κ*(*p*):=1−*H*_2_ (3*p*/4), we may then write


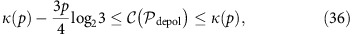


for *p*⩽2/3, while 0 otherwise (Fig. 8a). The result can be extended to any dimension *d*≥2. A qudit depolarising channel is defined as in equation (35) up to using the mixed state *I*/*d*. Setting *f*:=*p*(*d*^2^−1)/*d*^2^ and 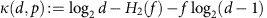, we find


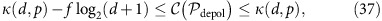


for *p*⩽*d*/(*d*+1), while zero otherwise.

Consider now the dephasing channel. This is a Pauli channel, which deteriorates quantum information without energy decay, as it occurs in spin-spin relaxation or photonic scattering through waveguides. It is defined as


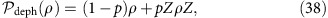


where *p* is the probability of a phase flip. We can easily check that the two bounds of equation (34) coincide, so that this channel is distillable and its two-way capacities are


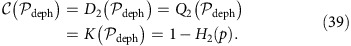


Note that this also proves 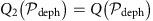, where the latter was derived in ref. 53.

For an arbitrary qudit with computational basis {|*j*〉}, the generalized dephasing channel is defined as


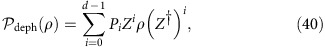


where *P*_*i*_ is the probability of *i* phase flips, with a single flip being 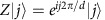. This channel is distillable and its two-way capacities are functionals of **P**={*P*_*i*_}


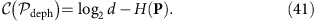


### Quantum erasure channel

A simple decoherence model is the erasure channel. This is described by


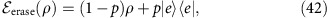


where *p* is the probability of getting an orthogonal erasure state 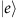. We already know that 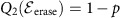 (ref. 12). Therefore, we compute the secret-key capacity.

Following ref. 12, one shows that 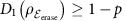. In fact, suppose that Alice sends halves of EPR states to Bob. A fraction 1−*p* will be perfectly distributed. These good cases can be identified by Bob applying the measurement 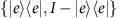 on each output system, and communicating the results back to Alice in a single and final CC. Therefore, they distill at least 1−*p* ebits per copy. It is then easy to check that this channel is Choi-stretchable and we compute 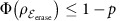. Thus, the erasure channel is distillable and we may write


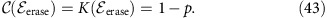


In arbitrary dimension *d*, the generalized erasure channel is defined as in equation (42), where *ρ* is now the state of a qudit and the erasure state 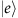 lives in the extra *d*+1 dimension. We can easily generalize the previous derivations to find that this channel is distillable and


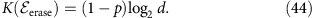


Note that the latter result can also be obtained by computing the squashed entanglement of the erasure channel, as shown by the independent derivation of ref. 54.

### Amplitude damping channel

Finally, an important model of decoherence in spins or optical cavities is energy dissipation or amplitude damping^55^,^56^. The action of this channel on a qubit is


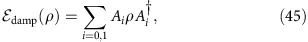


where 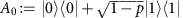, 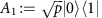, and *p* is the damping probability. Note that 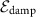 is not teleportation-covariant. However, it is decomposable as


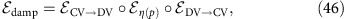


where 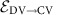 teleports the original qubit into a single-rail bosonic qubit^9^; then, 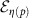 is a lossy channel with transmissivity *η*(*p*):=1−*p*; and 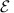
_CV→DV_ teleports the single-rail qubit back to the original qubit. Thus, 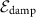 is stretchable into the asymptotic Choi matrix of the lossy channel 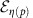. This shows that we need a dimension-independent theory even for stretching DV channels.

From Theorem 5 we get 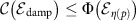, implying


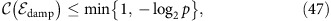


while the reverse coherent information implies^14^


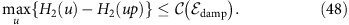


The bound in equation (47) is simple but only good for strong damping (*p*>0.9). A shown in Fig. 8b, we find a tighter bound using the squashed entanglement, that is,


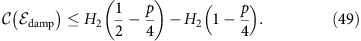


## Discussion

In this work, we have established the ultimate rates for point-to-point quantum communication, entanglement distribution and secret-key generation at any dimension, from qubits to bosonic systems. These limits provide the fundamental benchmarks that only quantum repeaters may surpass. To achieve our results we have designed a general reduction method for adaptive protocols, based on teleportation stretching and the relative entropy of entanglement, suitably extended to quantum channels. This method has allowed us to bound the two-way capacities (*Q*_2_, *D*_2_ and *K*) with single-letter quantities, establishing exact formulas for bosonic lossy channels, quantum-limited amplifiers, dephasing and erasure channels, after about 20 years since the first studies^12^,^31^.

In particular, we have characterized the fundamental rate-loss scaling which affects any quantum optical communication, setting the ultimate achievable rate for repeaterless QKD at 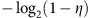 bits per channel use, that is, about 1.44*η* bits per use at high loss. There are two remarkable aspects to stress about this bound. First, it remains sufficiently tight even when we consider input energy constraints (down to ≃1 mean photon). Second, it can be reached by using one-way CCs with a maximum cost of just 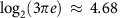 classical bits per channel use; this means that our bound directly provides the throughput in terms of bits per second, once a clock is specified (see Supplementary Note 7 for more details).

Our reduction method is very general and goes well beyond the scope of this work. It has been already used to extend the results to quantum repeaters.^57^ has shown how to simplify the most general adaptive protocols of quantum and private communication between two end-points of a repeater chain and, more generally, of an arbitrary multi-hop quantum network, where systems may be routed though single or multiple paths. Depending on the type of routing, the end-to-end capacities are determined by quantum versions of the widest path problem and the max-flow min-cut theorem. More recently, teleportation stretching has been also used to completely simplify adaptive protocols of quantum parameter estimation and quantum channel discrimination^58^. See Supplementary Discussion for a summary of our findings, other follow-up works and further remarks.

## Methods

### Basics of bosonic systems and Gaussian states

Consider *n* bosonic modes with quadrature operators 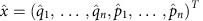. The latter satisfy the canonical commutation relations^59^


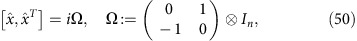


with *I*_*n*_ being the *n* × *n* identity matrix. An arbitrary multimode Gaussian state *ρ*(*u*, *V*), with mean value *u* and covariance matrix (CM) *V*, can be written as^60^


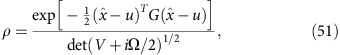


where the Gibbs matrix *G* is specified by


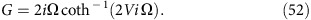


Using symplectic transformations^2^, the CM *V* can be decomposed into the Williamson's form 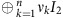 where the generic symplectic eigenvalue *ν*_*k*_ satisfies the uncertainty principle *ν*_*k*_≥1/2. Similarly, we may write *ν*_*k*_=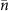
_*k*_+1/2 where 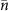
_*k*_ are thermal numbers, that is, mean number of photons in each mode. The von Neumann entropy of a Gaussian state can be easily computed as


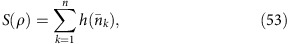


where *h*(*x*) is given in equation (24).

A two-mode squeezed vacuum (TMSV) state Φ^*μ*^ is a zero-mean pure Gaussian state with CM


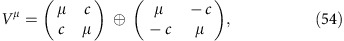


where 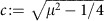 and *μ*=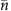
+1/2. Here 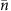 is the mean photon number of the reduced thermal state associated with each mode *A* and *B*. The Wigner function of a TMSV state Φ^*μ*^ is the Gaussian


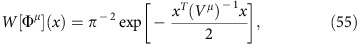


where *x* :=(*q*_*A*_, *q*_*B*_, *p*_*A*_, *p*_*B*_)^*T*^. For large *μ*, this function assumes the delta-like expression^25^


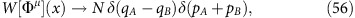


where *N* is a normalization factor, function of the anti-squeezed quadratures *q*_+_:=*q*_*A*_+*q*_*B*_ and *p*_−_:=*p*_*A*_−*p*_*B*_, such that 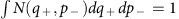. Thus, the infinite-energy limit of TMSV states 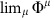 defines the asymptotic CV EPR state Φ, realizing the ideal EPR conditions 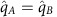 for position and 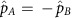 for momentum.

Finally, recall that single-mode Gaussian channels can be put in canonical form^2^, so that their action on input quadratures 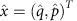 is


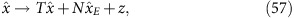


where *T* and *N* are diagonal matrices, *E* is an environmental mode with 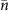
_*E*_ mean photons, and *z* is a classical Gaussian variable, with zero mean and CM *ξI* ≥0.

### Relative entropy between Gaussian states

We now provide a simple formula for the relative entropy between two arbitrary Gaussian states *ρ*_1_(*u*_1_, *V*_1_) and *ρ*_2_(*u*_2_, *V*_2_) directly in terms of their statistical moments. Because of this feature, our formula supersedes previous expressions^61^,^62^. We have the following.

*Theorem* 7: For two arbitrary multimode Gaussian states, *ρ*_1_(*u*_1_, *V*_1_) and *ρ*_2_(*u*_2_, *V*_2_), the entropic functional


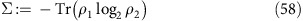


is given by


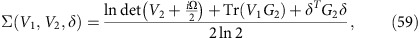


where *δ*:=*u*_1_−*u*_2_ and *G*:=*g*(*V*) as given in equation (52). As a consequence, the von Neumann entropy of a Gaussian state *ρ*(*u*, *V*) is equal to


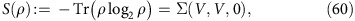


and the relative entropy of two Gaussian states *ρ*_1_(*u*_1_, *V*_1_) and *ρ*_2_(*u*_2_, *V*_2_) is given by


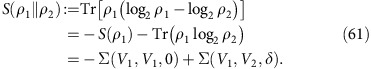


*Proof*: The starting point is the use of the Gibbs-exponential form for Gaussian states^60^ given in equation (51). Start with zero-mean Gaussian states, which can be written as 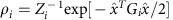, where *G*_*i*_=*g*(*V*_*i*_)is the Gibbs-matrix and *Z*_*i*_=det(*V*_*i*_+*i*Ω/2)^1/2^ is the normalization factor (with *i*=1, 2). Then, replacing into the definition of Σ given in equation (58), we find


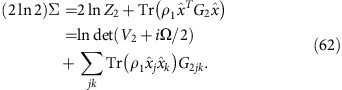


Using the commutator 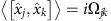 and the anticommutator 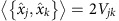, we derive


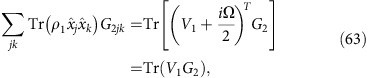


where we also exploit the fact that Tr(Ω*G*)=0, because Ω is antisymmetric and *G* is symmetric (as *V*).

Let us now extend the formula to non-zero mean values (with difference *δ*=*u*_1_−*u*_2_). This means to perform the replacement 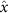
→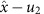, so that


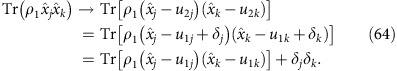


By replacing this expression in equation (63), we get


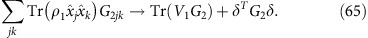


Thus, by combining equations (62) and (65), we achieve equation (59). The other equations (60) and (61) are immediate consequences. This completes the proof of Theorem 7.

As discussed in ref. 60, the Gibbs-matrix *G* becomes singular for a pure state or, more generally, for a mixed state containing vacuum contributions (that is, with some of the symplectic eigenvalues equal to 1/2). In this case the Gibbs-exponential form must be used carefully by making a suitable limit. Since Σ is basis independent, we can perform the calculations in the basis in which *V*_2_, and therefore *G*_2_, is diagonal. In this basis


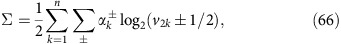


where {*v*_2*k*_} is the symplectic spectrum of *V*_2_, and


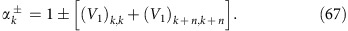


Now, if *v*_2*k*_=1/2 for some *k*, then its contribution to the sum in equation (66) is either zero or infinity.

### Basics of quantum teleportation

Ideal teleportation exploits an ideal EPR state 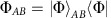 of systems *A* (for Alice) and *B* (for Bob). In finite dimension *d*, this is the maximally entangled Bell state


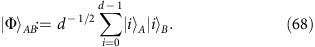


In particular, it is the usual Bell state 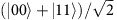 for a qubit. To teleport, we need to apply a Bell detection 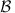 on the input system *a* and the EPR system *A* (that is, Alice's part of the EPR state). This detection corresponds to projecting onto a basis of Bell states 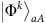 where the outcome *k* takes *d*^2^ values with probabilities *p*_*k*_=*d*^−2^.

More precisely, the Bell detection is a positive-operator valued measure with operators


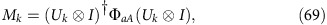


where 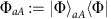 is the Bell state as in equation (68) and *U*_*k*_ is one of *d*^2^ teleportation unitaries, corresponding to generalized Pauli operators (described below). For any state *ρ* of the input system *a*, and outcome *k* of the Bell detection, the other EPR system *B* (Bob's part) is projected onto *U*_*k*_*ρ*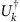. Once Alice has communicated *k* to Bob (feed-forward), he applies the correction unitary *U*_*k*_^−1^ to retrieve the original state *ρ* on its system *B*. Note that this process also teleports all correlations that the input system *a* may have with ancillary systems.

For CV systems (*d*→+∞), the ideal EPR source Φ_*AB*_ can be expressed as a TMSV state Φ^*μ*^ in the limit of infinite-energy *μ* →+ ∞. The unitaries *U*_*k*_ are phase-space displacements *D*(*k*) with complex amplitude *k* (ref. 9). The CV Bell detection is also energy-unbounded, corresponding to a projection onto the asymptotic EPR state up to phase-space displacements *D*(*k*). To deal with this, we need to consider a finite-energy version of the measurement, defined as a quasi-projection onto displaced versions of the TMSV state Φ^*μ*^ with finite parameter *μ*. This defines a positive-operator valued measure 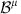 with operators


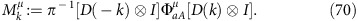


Optically, this can be interpreted as applying a balanced beam-splitter followed by two projections, one onto a position-squeezed state and the other onto a momentum-squeezed state (both with finite squeezing). The ideal CV Bell detection 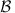 is reproduced by taking the limit of *μ*→+∞ in equation (70). Thus, CV teleportation must always be interpreted *a la* Braunstein and Kimble^25^, so that we first consider finite resources 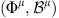 to compute the *μ*-dependent output and then we take the limit of large *μ*.

### Teleportation unitaries

Let us characterize the set of teleportation unitaries 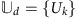 for a qudit of dimension *d*. First, let us write *k* as a multi-index *k*=(*a*, *b*) with 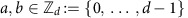. The teleportation set is therefore composed of *d*^2^ generalized Pauli operators 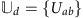, where *U*_*ab*_ :=*X*^*a*^*Z*^*b*^. These are defined by introducing unitary (non-Hermitian) operators


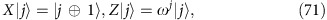


where ⊕ is the modulo *d* addition and


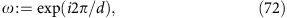


so that they satisfy the generalized commutation relation


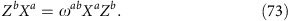


Note that any qudit unitary can be expanded in terms of these generalized Pauli operators. We may construct the set of finite-dimensional displacement operators *D*(*j*, *a*, *b*):=*ω*^*j*^*X*^*a*^*Z*^*b*^ with 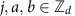 which form the finite-dimensional Weyl–Heisenberg group (or Pauli group). For instance, for a qubit (*d*=2), we have 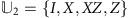 and the group ±1 × {*I*, *X*, *XZ*, *Z*}. For a CV system (*d*=+∞), the teleportation set is composed of infinite displacement operators, that is, we have 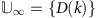, where *D*(*k*) is a phase-space displacement operator^2^ with complex amplitude *k*. This set is the infinite-dimensional Weyl–Heisenberg group.

It is important to note that, at any dimension (finite or infinite), the teleportation unitaries satisfy


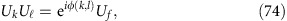


where *U*_*f*_ is another teleportation unitary and 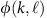 is a phase. In fact, for finite *d*, let us write *k* and 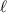 as multi-indices, that is, *k*=(*a*, *b*) and 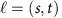. From 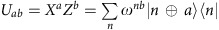, we see that 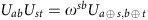. Then, for infinite *d*, we know that the displacement operators satisfy 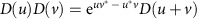, for any two complex amplitudes *u* and *v*.

Now, let us represent a teleportation unitary as


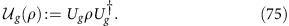


It is clear that we have 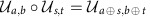 for DV systems, and 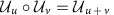 for CV systems. Therefore 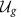 satisfies the group structure


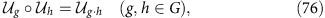


where *G* is a product of two groups of addition modulo *d* for DVs, while *G* is the translation group for CVs. Thus, the (multi-)index of the teleportation unitaries can be taken from the abelian group *G*.

### Teleportation-covariant channels

Let us a give a group representation to the property of teleportation covariance specified by equation (9). Following equation (75), we may express an arbitrary teleportation unitary as 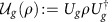 where *g*∈*G*. Calling 
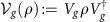
, we see that equation (9) implies


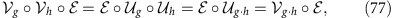


so that 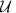 and 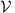 are generally different unitary representations of the same abelian group *G*. Thus, equation (9) can also be written as


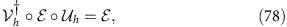


for all *h*∈*G*, where 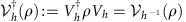.

The property of equation (9) is certainly satisfied if the channel is covariant with respect to the Weyl–Heisenberg group, describing the teleportation unitaries in both finite- and infinite-dimensional Hilbert spaces. This happens when the channel is dimension-preserving and we may set *V*_*k*_=*U*_*k*′_ for some *k*′ in equation (9). Equivalently, this also means that 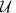 and 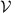 are exactly the same unitary representation in equation (78). We call ‘Weyl-covariant' these specific types of tele-covariant channels.

In finite-dimension, a Weyl-covariant channel must necessarily be a Pauli channel. In infinite-dimension, a Weyl-covariant channel commutes with displacements, which is certainly a property of the bosonic Gaussian channels. A simple channel that is tele-covariant but not Weyl-covariant is the erasure channel. This is in fact dimension-altering (since it adds an orthogonal state to the input Hilbert space) and the output correction unitaries to be used in equation (9) have the augmented form *V*_*k*_=*U*_*k*_⊕*I*. Hybrid channels, mapping DVs into CVs or vice versa, cannot be Weyl-covariant but they may be tele-covariant. Finally, the amplitude damping channel is an example of a channel, which is not tele-covariant.

Note that, for a quantum channel in finite dimension, we may easily re-write equation (9) in terms of an equivalent condition for the Choi matrix. In fact, by evaluating the equality in equation (9) on the EPR state 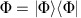 and using the property that 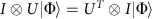, one finds


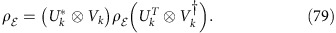


Thus, a finite-dimensional 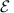 is tele-covariant if and only if, for any teleportation unitary *U*_*k*_, we may write


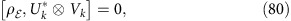


for another generally different unitary *V*_*k*_. There are finite-dimensional channels satisfying conditions stronger than equation (80). For Pauli channels, we may write 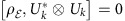 for any *k*, that is, the Choi matrix is invariant under twirling operations restricted to the generators of the Pauli group {*U*_*k*_}. For depolarising channels, we may even write 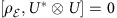 for an arbitrary unitary *U*. This means that the Choi matrix of a depolarising channel is an isotropic state.

### LOCC-averaging in teleportation stretching

Consider an arbitrary adaptive protocol described by some fundamental preparation of the local registers *ρ*_**a**_^0^⊗*ρ*_**b**_^0^ and a sequence of adaptive LOCCs 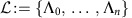. In general, these LOs may involve measurements. Call *u*_*i*_ the (vectorial) outcome of Alice's and Bob's local measurements performed within the *i*th adaptive LOCC, so that 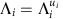. It is clear that 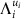 will be conditioned by measurements and outcomes of all the previous LOCCs, so that a more precise notation will be 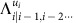 where the output *u*_*i*_ is achieved with a conditional probability 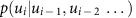. After *n* transmissions, we have a sequence of outcomes **u**=*u*_0_ … *u*_*n*_ with joint probability


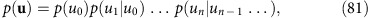


and a sequence of LOCCs


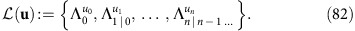


The mean rate of the protocol is achieved by averaging the output state over all possible outcomes **u**, which is equivalent to considering the output state generated by the trace-preserving LOCC-sequence 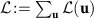.

In fact, suppose that the (normalized) output state 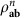
(**u**) generated by the conditional 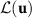 is epsilon-close to a corresponding target state *φ*_*n*_(**u**) with rate *R*_*n*_(**u**). This means that we have 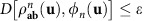 in trace distance. The mean rate of the protocol 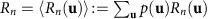 is associated with the average target state 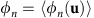. It is easy to show that *φ*_*n*_ is approximated by the mean output state 
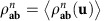
 generated by 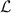. In fact, by using the joint convexity of the trace distance^1^, we may write


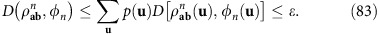


Now we show that the LOCC-simulation of a channel 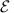 does not change the average output state 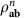 and this state can be re-organized in a block form. The *i*th (normalized) conditional output 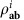 can be expressed in terms of the *i*−1th output 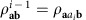 as follows


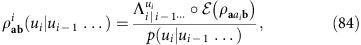


where 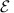 is meant as 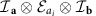 with *a*_*i*_ being the system transmitted. Thus, after *n* transmissions, the conditional output state is 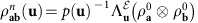, where


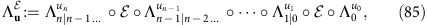


and the average output state is given by


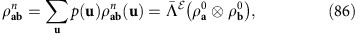


where 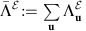.

For some LOCC 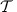 and resource state *σ*, let us write the simulation


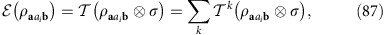


where 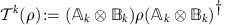 is Alice and Bob's conditional LOCC with probability *p*(*k*). For simplicity we omit other technical labels that may describe independent local measurements or classical channels, because they will also be averaged at the end of the procedure. Let us introduce the vector **k**=*k*_1_ … *k*_*n*_ where *k*_*i*_ identifies a conditional LOCC 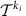 associated with the *i*th transmission. Because the LOCC-simulation of the channel is fixed, we have the factorized probability *p*(**k**)=*p*(*k*_1_) … *p*(*k*_*n*_).

By replacing the simulation in equation (84), we obtain


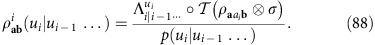


By iteration, the latter equation yields


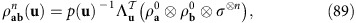


where


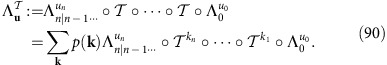


Therefore, the average output state of the original protocol may be equivalently expressed in the form


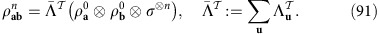


Finally, we may include the preparation *ρ*_**a**_^0^⊗*ρ*_**b**_^0^ in the LOCC, so that we may write


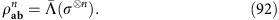


To extend this technical proof to CV systems, we perform the replacement 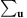
→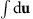 with the probabilities becoming probability densities. Then, 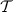 and *σ* may be both asymptotic, that is, defined as infinite-energy limits 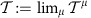 and 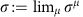 from corresponding finite-versions 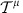 and *σ*^*μ*^. In this case, we repeat the previous procedure for some *μ* and then we take the limit on the output state 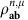.

### Details on Lemma 3 in relation to teleportation stretching with bosonic channels

For a bosonic channel, the Choi matrix and the ideal Bell detection are both energy-unbounded. Therefore, any Choi-based LOCC simulation of these channels must necessarily be asymptotic. Here we discuss in more detail how an asymptotic channel simulation 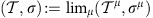 leads to an asymptotic form of stretching as described in Lemma 3. Any operation or functional applied to 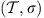 is implicitly meant to be applied to the finite-energy simulation 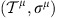, whose output then undergoes the *μ*-limit.

**Figure 9:**
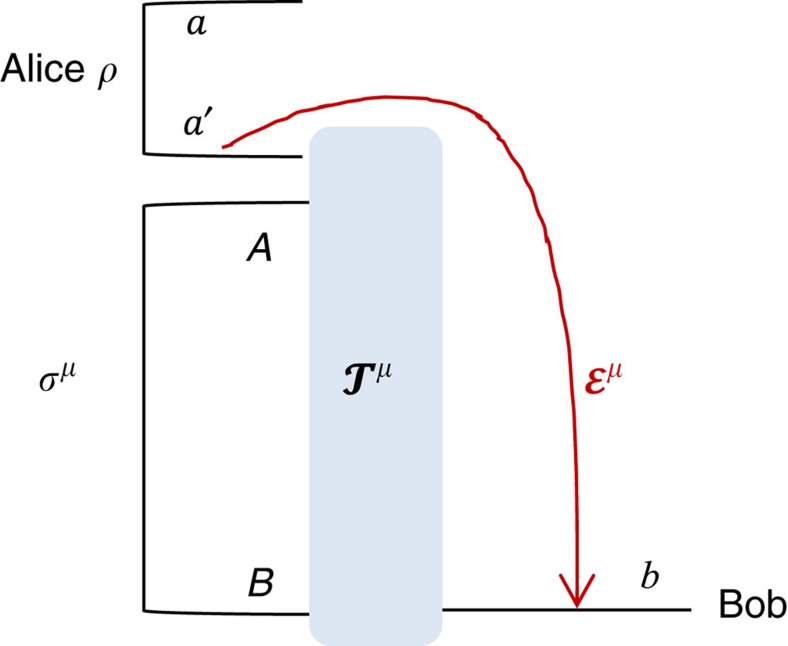
Asymptotic LOCC simulation of bosonic channels. The finite-energy LOCC simulation 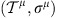 generates a teleportation channel 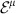. Assume that 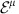 defines a target bosonic channel 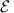 according to the pointwise limit in equation (93). Then, we say that the bosonic channel 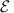 has asymptotic simulation 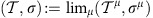.

Consider a bosonic channel 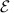 with asymptotic simulation 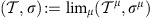. As depicted in Fig. 9, this means that there is a channel 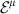 generated by 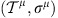 such that 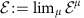 in the sense that


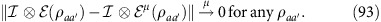


In other words, for any (energy-bounded) bipartite state *ρ*_*aa*′_, whose *a*′-part is propagated, the original channel output 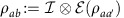 and the simulated channel output 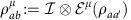 satisfy the limit


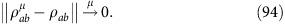


By teleportation stretching, we may equivalently decompose the output state *ρ*_*ab*_^*μ*^ into the form


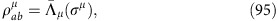


where 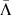
_*μ*_ is a trace-preserving LOCC, which is includes both 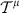 and the preparation of *ρ*_*aa*′_ (it is trace-preserving because we implicitly assume that we average over all possible measurements present in the simulation LOCC 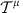). By taking the limit of *μ*→+∞ in equation (95), the state 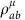 becomes the channel output state *ρ*_*ab*_ according to equation (94). Therefore, we have the limit


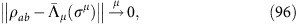


that we may compactly write as


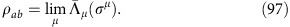


Note that we may express equation (93) in a different form. In fact, consider the set of energy-constrained bipartite states 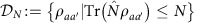, where 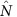 is the total number operator. Then, for two bosonic channels, 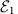 and 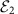, we may define the energy-bounded diamond norm


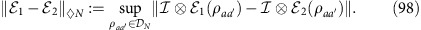


Using the latter definition and the fact that 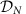 is a compact set, we have that the pointwise limit in equation (93) implies the following uniform limit


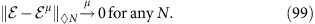


The latter expression is useful to generalize the reasoning to the adaptive protocol, with LOCCs applied before and after transmission. Consider the output 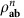 after *n* adaptive uses of the channel 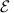, and the simulated output 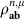, which is generated by replacing 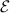 with the imperfect channel 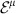. Explicitly, we may write


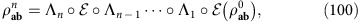


with its approximate version


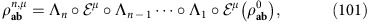


where it is understood that 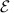 and 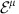 are applied to system *a*_*i*_ in the *i*th transmission, that is, 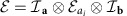.

Assume that the mean photon number of the total register states 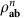 and 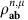 is bounded by some large but yet finite value *N*(*n*). For instance, we may consider a sequence *N*(*n*)=*N*(0)+*nt*, where *N*(0) is the initial photon contribution and *t* is the channel contribution, which may be negative for energy-decreasing channels (like the thermal-loss channel) or positive for energy-increasing channels (like the quantum amplifier). We then prove


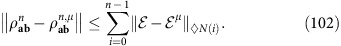


In fact, for *n*=2, we may write


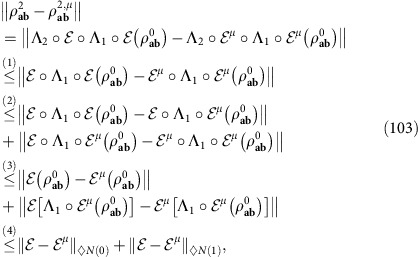


where: (1) we use monotonicity under Λ_2_; (2) we use the triangle inequality; (3) we use monotonicity with respect to 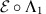; and (4) we use the definition of equation (98) assuming *a*′=*a*_*i*_ and the energy bound *N*(*n*). Generalization to arbitrary *n* is just a technicality.

By using equation (99) we may write that, for any bound *N*(*n*) and *ɛ*≥0, there is a sufficiently large *μ* such that 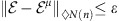, so that equation (102) becomes


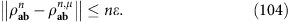


By applying teleportation stretching we derive 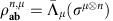, where 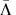
_*μ*_ includes the original LOCCs Λ_*i*_ and the teleportation LOCCs 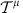. Thus, equation (104) implies


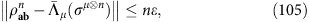


or, equivalently, 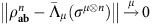.

Therefore, given an adaptive protocol with arbitrary register energy, and performed *n* times through a bosonic channel 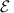 with asymptotic simulation, we may write its output state as the (trace-norm) limit


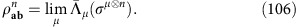


This means that we may formally write the asymptotic stretching 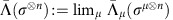 for an asymptotic channel simulation 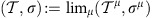.

### More details on the one-shot REE bound given in Theorem 5

The main steps for proving equation (15) are already given in the main text. Here we provide more details of the formalism for the specific case of bosonic channels, involving asymptotic simulations 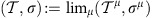. Given the asymptotic stretching of the output state 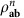 as in equation (106), the simplification of the REE bound *E*_R_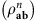 explicitly goes as follows


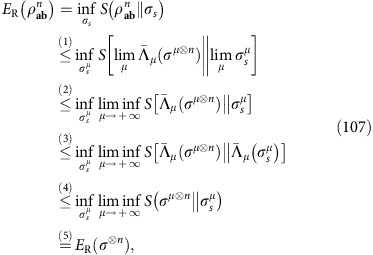


where: (1) 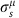 is a generic sequence of separable states that converges in trace norm, that is, such that there is a separable state 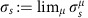 so that 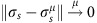; (2) we use the lower semi-continuity of the relative entropy^3^; (3) we use that 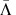
_*μ*_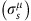 are specific types of converging separable sequences within the set of all such sequences; (4) we use the monotonicity of the relative entropy under trace-preserving LOCCs; and (5) we use the definition of REE for asymptotic states given in equation (4).

Thus, from Theorem 1, we may write the following upper bound for the two-way capacity of a bosonic channel


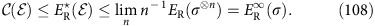


The supremum over all adaptive protocols, which defines 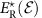 disappears in the right hand side of equation (108). The resulting bound applies to both energy-constrained protocols and the limit of energy-unconstrained protocols. The proof of the further condition 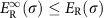 in equation (15) comes from the subadditivity of the REE over tensor product states. This subadditivity also holds for a tensor product of asymptotic states; it is proven by restricting the minimization on tensor product sequences 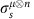 in the corresponding definition of the REE.

Let us now prove equation (16). The two inequalities in equation (16) are simply obtained by using 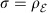 for a Choi-stretchable channel (where the Choi matrix is intended to be asymptotic for a bosonic channel). Then we show the equality 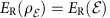. By restricting the optimization in 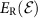 to an input EPR state Φ, we get the direct part 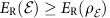 as already noticed in equation (6). For CVs, this means to choose an asymptotic EPR state 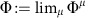, so that


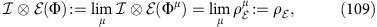


and therefore


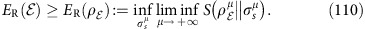


For the converse part, consider first DVs. By applying teleportation stretching to a single use of the channel 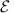, we may write 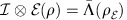 for a trace-preserving LOCC 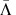. Then, the monotonicity of the REE leads to


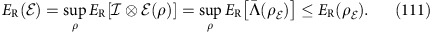


For CVs, we have an asymptotic stretching 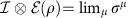 where 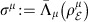. Therefore, we may write


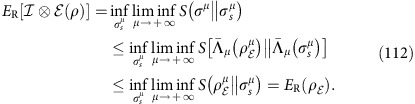


Since this is true for any *ρ*, it also applies to the supremum and, therefore, to the channel's REE 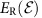.

### Proof of Proposition 6 on the one-shot REE bound for DV channels

At finite dimension, we may first use teleportation stretching to derive 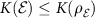 and then apply any upper bound to the distillable key 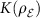, among which the REE bound has the best performance. Consider a key generation protocol described by a sequence 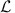 of adaptive LOCCs (implicitly assumed to be averaged). If the protocol is implemented over a Choi-stretchable channel 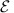 in finite dimension *d*, its stretching allows us to write the output as 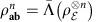 for a trace-preserving LOCC 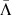. Since any LOCC-sequence 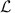 is transformed into 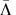, any key generation protocol through 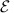 becomes a key distillation protocol over copies of the Choi matrix 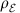. For large *n*, this means 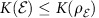.

To derive the opposite inequality, consider Alice sending EPR states through the channel, so that the shared output will be 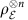. There exists an optimal LOCC on these states, which reaches the distillable key 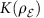 for large *n*. This is a specific key generation protocol over 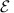, so that we may write 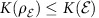. Thus, for a *d*-dimensional Choi-stretchable channel, we find


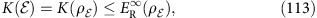


where we also exploit the fact that the distillable key of a DV state is bounded by its regularized REE^27^. It is also clear that 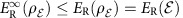, where the latter equality is demonstrated in the proof of Theorem 5.

Note that 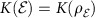 cannot be directly written for a bosonic channel, because its Choi matrix 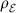 is energy-unbounded, so that its distillable key 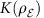 is not well-defined. By contrast, we know how to extend 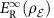 to bosonic channels and show 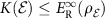 at any dimension: This is the more general procedure of Theorem 5, which first exploits the general REE bound 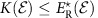 and then simplifies 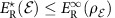 by means of teleportation stretching at any dimension.

### Two-way quantum communication

Our method can be extended to more complex forms of quantum communication. In fact, our weak converse theorem can be applied to any scenario where two parties produce an output state by means of an adaptive protocol. All the details of the protocol are contained in the LOCCs 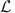 which are collapsed into 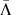 by teleportation stretching and then discarded using the REE.

**Figure 10:**
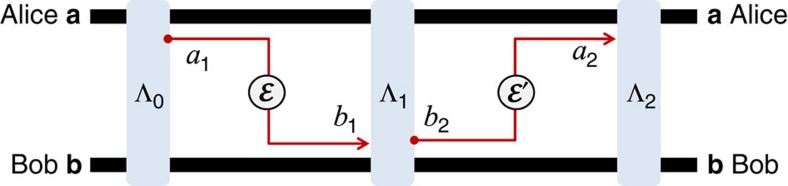
Adaptive protocol for two-way quantum or private communication. The protocol employs a forward channel 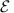 and backward channel 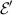. Transmissions are alternated with adaptive LOCCs 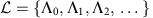.

Consider the scenario where Alice and Bob send systems to each other by choosing between two possible channels, 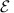 (forward) or 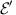 (backward), and performing adaptive LOCC after each single transmission (see also Fig. 10). The capacity 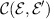 is defined as the maximum number of target bits distributed per individual transmission, by using one of the two channels 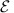 and 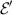, and assuming LOs assisted by unlimited two-way CCs.

In general, the feedback transmission may occur a fraction *p* of the rounds, with associated capacity


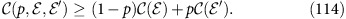


The lower bound is a convex combination of the individual capacities of the two channels, which is achievable by using independent LOCC-sequences for the two channels.

Assume that 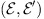 are stretchable into the pair of resource states (*σ*, *σ*′). Then, we can stretch the protocol and decompose the output state as


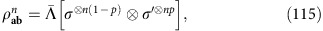


where the tensor exponents *n*(1−*p*) and *np* are integers for suitably large *n* (it is implicitly understood that we consider suitable limits in the bosonic case). Using the monotonicity of the REE under trace-preserving LOCCs and its subadditivity over tensor products, we write


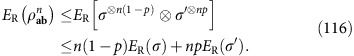


As previously said, our weak converse theorem can be applied to any adaptive protocol where two parties finally share a bipartite state 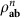. Thus, we may write


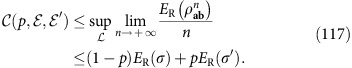


From equations (114) and (117), we find that 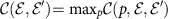 must satisfy


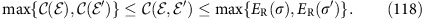


For Choi-stretchable channels, this means


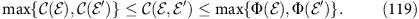


In particular, if the two channels are distillable, that is, 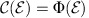 and 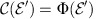, then we may write


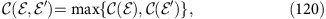


and the optimal strategy (value of *p*) corresponds to using the channel with maximum capacity.

Note that we may also consider a two-way quantum communication protocol where the forward and backward transmissions occur simultaneously, and correspondingly define a capacity that quantifies the maximum number of target bits which are distributed in each double communication, forward and backward (instead of each single transmission, forward or backward). However, this case can be considered as a double-band quantum channel.

### Multiband quantum channel

Consider the communication scenario where Alice and Bob can exploit a multiband quantum channel, that is, a quantum channel whose single use involves the simultaneous transmission of *m* distinct systems. In practice, this channel 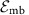 is represented by a set of *m* independent channels or bands 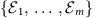, that is, it can be written as


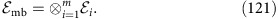


For instance, the bands may be bosonic Gaussian channels associated with difference frequencies.

In this case, the adaptive protocol is modified in such a way that each (multiband) transmission involves Alice simultaneously sending *m* quantum systems to Bob. These *m* input systems may be in a generally entangled state, which may also involve correlations with the remaining systems in Alice's register. Before and after each multiband transmission, the parties perform adaptive LOCCs on their local registers **a** and **b**. The multiband protocol is therefore characterized by a LOCC sequence 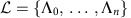 after *n* transmissions.

The definition of the generic two-way capacity is immediately extended to a multiband channel. This capacity quantifies the maximum number of target bits that are distributed (in parallel) for each multiband transmission by means of adaptive protocols. It must satisfy


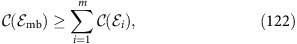


where the lower bound is the sum of the two-way capacities of the single bands 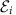. This lower bound is obtained by using adaptive LOCCs that are independent between different 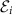, and considering an output state of the form 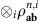 where 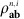 is the output associated with 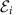.

Now consider an adaptive protocol performed over a multiband channel, whose *m* bands 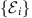 are stretchable into *m* resources states {*σ*_*i*_}. By teleportation stretching, we find that Alice and Bob's output state can be decomposed in the form


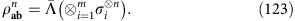


(it is understood that the formulation is asymptotic for bosonic channels). This previous decomposition leads to


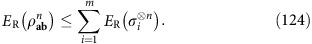


Using our weak converse theorem, we can then write


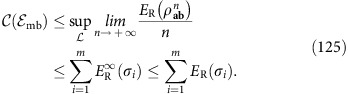


Combining equations (122) and (125) we may then write


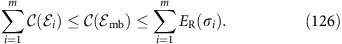


For Choi-stretchable bands, this means


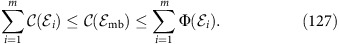


Finally, if the bands are distillable, that is, 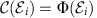, then we find the additive result


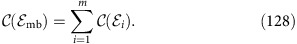


### Code availability

Source codes of the plots are available from the authors on request.

### Data availability

No relevant research data were generated in this study.

## Additional information

**How to cite this article:** Pirandola, S. *et al*. Fundamental limits of repeaterless quantum communications. *Nat. Commun.* **8,** 15043 doi: 10.1038/ncomms15043 (2017).

**Publisher's note**: Springer Nature remains neutral with regard to jurisdictional claims in published maps and institutional affiliations.

## Supplementary Material

Supplementary Information Supplementary Figures, Supplementary Notes, Supplementary Discussion and Supplementary References.
