# Supplementary material for: Fundamental limits of repeaterless quantum communications
Source: Nat Commun. 2017 Apr 26;8:15043. doi: 10.1038/ncomms15043 (PMC5414096; doi:10.1038/ncomms15043)
Supplement: Supplementary Information — Supplementary Figures, Supplementary Notes, Supplementary Discussion and Supplementary References. [file ncomms15043-s1.pdf]

Supplementary Information

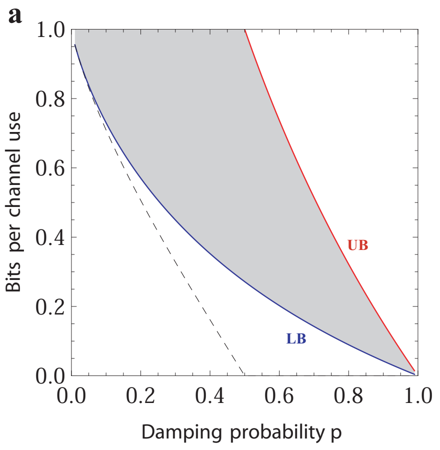

Supplementary Figure 1: Two-way capacity of the amplitude damping channel $\mathcal{E}_{\rm damp}$ with probability p. (**a**) The two-way capacity $\mathcal{C}(\mathcal{E}_{\rm damp})$ is contained in the shadowed area identified by the lower bound (LB) and the upper bound (UB) of Eq. (214). Note the separation from the unassisted quantum capacity Q of the channel (dashed). (**b**) More precisely, $\mathcal{C}(\mathcal{E}_{\rm damp})$ is contained in the shadowed area identified by the lower bound (LB) and the upper bound (UB) of Eq. (235). We also plot the unassisted quantum capacity Q of the channel (dashed), the REE upper bound of Eq. (211) (solid), and the bound of ref. [28] (dotted).

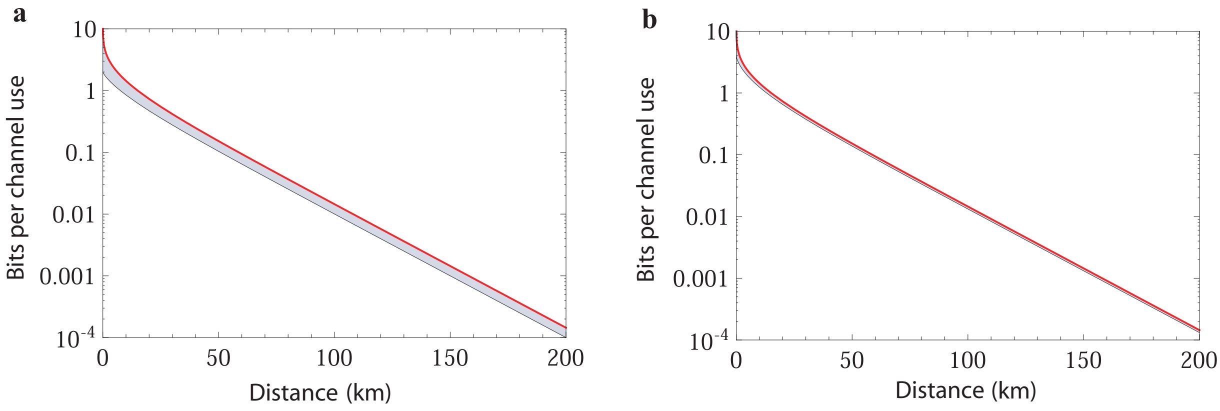

Supplementary Figure 2: Study of the tightness of our unconstrained upper bound for the lossy channel under the assumption of energy-constrained inputs. (**a**) We plot the unconstrained upper bound $\Phi(\eta) = -\log_2(1-\eta)$ (upper red line) and the constrained lower bound $I_{\rm RC}(\bar{m},\eta)$ (lower black line) given by the reverse coherent information in Eq. (247) assuming $\bar{m} = 1$ mean photons at the input. Both are plotted in terms of the distance (km) assuming the standard loss rate of 0.2dB/km. The constrained two-way capacity of the lossy channel is in the middle dark area. (**b**) Same as in (**a**) but now with $\bar{m} = 5$ mean photons. We see how the unconstrained upper bound is rapidly reached already with a few photons.

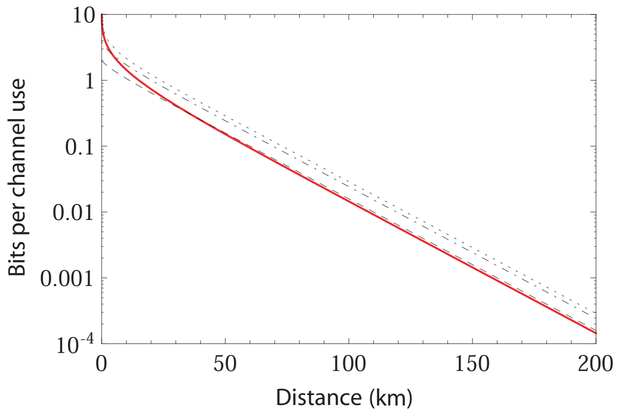

Supplementary Figure 3: Comparison with previous bounds based on the squashed entanglement. We compare the unconstrained upper bound $\Phi(\eta) = -\log_2(1-\eta)$ (solid red line) with the unconstrained TGW bound (dotted), and the constrained TGW bound for $\bar{m} = 5$ mean photons (dashed-dotted) and $\bar{m} = 1$ mean photons (dashed line). Bounds are plotted in terms of distance (km) assuming the standard loss rate of 0.2dB/km. Note that $\Phi(\eta)$ remains the tighter upper bound even if we constrain the input energy down to one mean photon. This is true everywhere, except for short distances (where the energy constraint is not so interesting since we can efficiently use highly-modulated CV-QKD).

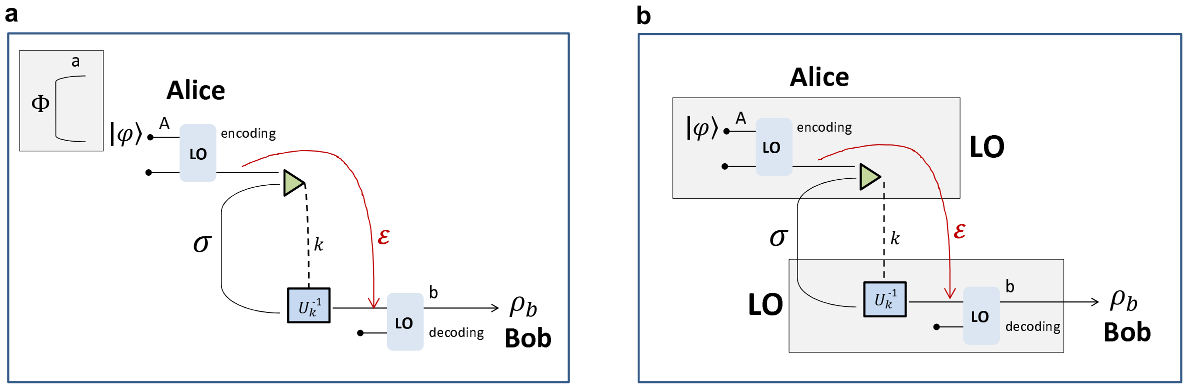

Supplementary Figure 4: Different approaches to reduce quantum communication. (**a**) Precursory BDSW reduction argument [55, Section V], explicitly considered for 2-way CCs. This may be described in 3 steps. (1) Suppose that Alice and Bob implement a QC protocol for transmitting qubits from system A to system b by means of channel $\mathcal{E}$ (red curvy line). In the upper LO, Alice applies a suitable quantum error correcting code (QECC) $\Lambda_{\text{enc}}^{m\to n}$ to encode an m-qubit logical state $|\varphi^{(m)}\rangle$ into an n-qubit codeword which is sent through $\mathcal{E}^{\otimes n}$. In the lower LO, Bob applies a decoding operation $\Lambda_{\text{dec}}^{n\to m}$, so that $\Lambda_{\text{dec}}^{n\to m} \circ \mathcal{E}^{\otimes n} \circ \Lambda_{\text{enc}}^{m\to n}$ tends to the identity, and the n-use output state ρ_b^n approximates $|\varphi^{(m)}\rangle\langle\varphi^{(m)}|$. In the general case, we assume that the previous LOs are assisted by unlimited two-way CCs between Alice and Bob. By optimizing over all QECCs and in the limit of infinite channel uses, one defines the two-way quantum capacity $Q_2(\mathcal{E})$. (2) Notice that Alice can use the QECC to send part of m ebits (see the Bell state Φ in the grey box), so that Alice and Bob share an output state ρ_{ab}^n which approximates $\Phi^{\otimes m}$. Assuming an asymptotic and optimal QECC, each ebit is reliably shared at the quantum capacity rate $Q_2(\mathcal{E})$. (3) Finally, assume that the channel $\mathcal{E}$ can be described by teleportation over the resource state σ. Any entanglement distribution strategy through channel $\mathcal{E}$ can therefore be seen as a specific protocol of entanglement distillation applied to the copies of σ. This observation leads to $Q_2(\mathcal{E}) \leq D_2(\sigma)$. (**b**) Different re-organization of the quantum operations in teleportation stretching. When we apply teleportation stretching to a QC protocol, we directly reduce the output state as follows $\rho_b(\mathcal{E}^{\otimes n}) = \bar{\Lambda}(\sigma^{\otimes n})$, for a trace-preserving LOCC $\bar{\Lambda}$ which is not connected with ED, but collapses the preparation $|\varphi^{(m)}\rangle\langle\varphi^{(m)}|$, the encoding/decoding maps, and all the teleportation operations. This is not asymptotic but done for any n.

Supplementary Note 1. PRELIMINARY TECHNICAL TOOLS

Truncation of infinite-dimensional Hilbert spaces

In the following it will be useful to use truncation tools which enables us to connect continuous-variable (CV) and discrete-variable (DV) states. Consider m bosonic modes with Hilbert space $\mathcal{H}^{\otimes m}$ and space of density operators $\mathcal{D}(\mathcal{H}^{\otimes m})$. Then, consider the energy operator $\hat{H} = \sum_{i=1}^{m} \hat{N}_i$ (with $\hat{N}_i$ being the number operator of mode i) and the following compact set of energy-constrained states [1]

$$\mathcal{D}_E(\mathcal{H}^{\otimes m}) := \{\rho \in \mathcal{D}(\mathcal{H}^{\otimes m}) \mid \mathrm{Tr}(\rho \hat{H}) \leq E\}. \tag{1}$$

It is easy to show that every such state is essentially supported on a finite-dimensional Hilbert space.

Lemma 1: Consider an energy-constrained m-mode bosonic state $\rho \in \mathcal{D}_E(\mathcal{H}^{\otimes m})$. There exists a finite-dimensional projector P_d which projects this state onto a d-dimensional support of the m-mode Hilbert space with probability

$$\mathrm{Tr}(\rho P_d) \geq 1 - \gamma, \quad \gamma := \frac{E}{\sqrt[m]{d} - 1}. \tag{2}$$

Correspondingly, the trace distance between the original state ρ and the d-dimensional truncated state

$$\delta := \frac{P_d \rho P_d}{\mathrm{Tr}(\rho P_d)} \tag{3}$$

satisfies the inequality

$$D(\rho, \delta) := \frac{1}{2} \|\rho - \delta\| \leq \sqrt{\gamma}. \tag{4}$$

Proof: Let us arrange the degenerate eigenvalues of $\hat{H}$ in increasing order as $h_0 \leq h_1 \leq \ldots \leq h_n \leq \ldots$. Each eigenvalue is computed as $\sum_{i=1}^{m} N_i$ where N_i is the photon number of mode i. The corresponding eigenstates are of the type $|\tilde{h}_n\rangle = |N_1\rangle \otimes \ldots \otimes |N_m\rangle$. For instance

$$\begin{aligned}
|\tilde{h}_0\rangle &= |0\rangle \otimes |0\rangle \otimes \ldots \otimes |0\rangle, \quad (h_0 = 0), \\
|\tilde{h}_1\rangle &= |1\rangle \otimes |0\rangle \otimes \ldots \otimes |0\rangle, \quad (h_1 = 1), \\
|\tilde{h}_2\rangle &= |0\rangle \otimes |1\rangle \otimes \ldots \otimes |0\rangle, \quad (h_2 = 1), \\
&\vdots \qquad\qquad\qquad\qquad\qquad \vdots
\end{aligned} \tag{5}$$

Note that $n+1$ counts the dimension of the truncated Hilbert space and we have $h_n \leq n$, because of the degeneracy of the eigenvalues. Since h_n is the total number of photons in all m modes, we have that each mode can have at most dimension $(h_n + 1)$, so that we may write the upper bound $n \leq n + 1 \leq (h_n + 1)^m$ or, equivalently,

$$h_n \geq \sqrt[m]{n} - 1 \tag{6}$$

Then, we proceed as in Refs. [1, 2]. Denote by $P_n := |\tilde{h}_n\rangle\langle\tilde{h}_n|$ the eigenprojector associated with $|\tilde{h}_n\rangle$. For dimension d, we consider the truncation projector

$$P_d := \sum_{n=0}^{d-1} P_n \ . \tag{7}$$

Therefore, for all $|\psi\rangle \in \mathcal{H}$, we may write

$$\langle\psi| h_d (I - P_d) |\psi\rangle = \langle\psi| \left[h_d \sum_{n=d}^{\infty} P_n \right] |\psi\rangle \leq \langle\psi| \left[\sum_{n=d}^{\infty} h_n P_n \right] |\psi\rangle \leq \langle\psi|\hat{H}|\psi\rangle. \tag{8}$$

This implies that, for all $\rho \in \mathcal{D}_E(\mathcal{H})$, we have

$$\mathrm{Tr}\left[\rho(I - P_d)\right] \leq \frac{1}{h_d} \mathrm{Tr}(\rho \hat{H}) \leq \frac{E}{h_d} \ . \tag{9}$$

According to Eq. (6), we may write $h_d \geq \sqrt[m]{d} - 1$, so that

$$\frac{E}{h_d} \leq \gamma := \frac{E}{\sqrt[m]{d} - 1}, \tag{10}$$

which proves Eq. (2). The proof of Eq. (4) is a simple modification of the one given by ref. [2]. □

Note that we may derive a similar result in terms of a truncation channel, i.e., by means of a completely positive trace-preserving (CPTP) map.

Lemma 2: Consider an energy-constrained m-mode bosonic state $\rho \in \mathcal{D}_E(\mathcal{H}^{\otimes m})$. There exists a truncation channel $\mathbb{T}_d$ which maps the state ρ into a truncated state $\tilde{\rho}$ defined over a d-dimensional support of the m-mode Hilbert space, such that

$$D(\rho, \tilde{\rho}) \leq \sqrt{\gamma} + \gamma, \tag{11}$$

where γ is defined in Eq. (2).

Proof: For any multimode energy-constrained bosonic state $\rho \in \mathcal{D}_E(\mathcal{H}^{\otimes m})$, we may define the following (non-local) truncation map

$$\tilde{\rho} := \mathbb{T}_d(\rho) = \sum_{i=0,1} \mathcal{E}_i \left(\Pi_i \rho \Pi_i^\dagger \right), \tag{12}$$

where $\Pi_0 := P_d$ and $\Pi_1 := I - P_d$, while for any projected state σ we have either the identity channel $\mathcal{E}_0(\sigma) = \sigma$ or the collapsing map $\mathcal{E}_1(\sigma) = \rho_0$, where ρ_0 is an arbitrary fixed state within the d-dimensional support. Setting $p := \mathrm{Tr}(\rho P_d)$, we may write

$$\tilde{\rho} = p\delta + (1-p)\rho_0, \tag{13}$$

where δ is defined in Eq. (3). Note that $S_0 := \|\rho - \rho_0\| \leq 2$. Then, by exploiting the convexity of the trace norm, we may write

$$D(\rho, \tilde{\rho}) = \frac{1}{2} \|\rho - \tilde{\rho}\| \leq \frac{p}{2} \|\rho - \delta\| + \frac{1-p}{2} S_0 \leq p\sqrt{\gamma} + 1 - p \leq \sqrt{\gamma} + \gamma, \tag{14}$$

where we have also used $p \leq 1$ and Lemma 1. □

Local CV-DV mappings

It is easy to modify the previous truncation tools to make them bipartite and local, i.e., based on LOs assisted by (generally two-way) CCs. Suppose that Alice and Bob share a CV bipartite state $\rho_{\mathbf{ab}}$, where Alice's local system $\mathbf{a}$ contains $m_{\mathbf{a}}$ modes and Bob's local system $\mathbf{b}$ contains $m_{\mathbf{b}}$ modes. Then, we may analyze how this state is transformed by a truncation channel which is based on LOCC. In fact, we may state the following.

Lemma 3: Consider an energy-constrained bosonic state $\rho_{\mathbf{ab}} \in \mathcal{D}_E(\mathcal{H}^{\otimes m_{\mathbf{a}}} \otimes \mathcal{H}^{\otimes m_{\mathbf{b}}})$ where Alice (Bob) has $m_{\mathbf{a}}$ ($m_{\mathbf{b}}$) modes. There is an LOCC truncation channel $\mathbb{T}_d^{\otimes}$ (local with respect to the bipartition $m_{\mathbf{a}} + m_{\mathbf{b}}$) which maps $\rho_{\mathbf{ab}}$ into a truncated state $\tilde{\rho}_{\mathbf{ab}}$ defined over a $d \times d$-dimensional support and such that

$$D(\rho_{\mathbf{ab}}, \tilde{\rho}_{\mathbf{ab}}) \leq \sqrt{\gamma} + \gamma, \quad \gamma := \frac{E}{\sqrt{d} - 1}. \tag{15}$$

The implementation of such truncation channel needs two bits of CC between Alice and Bob.

Proof: Assuming the bipartition of modes $m = m_A + m_B$, let us write the energy operator as $\hat{H} = \hat{H}_A + \hat{H}_B$, where

$$\hat{H}_A = \sum_{i=1}^{m_A} \hat{N}_i, \quad \hat{H}_B = \sum_{i=1+m_A}^{m_A+m_B} \hat{N}_i, \tag{16}$$

with $\hat{N}_i$ being the number operator of the i-th mode, with eigenstates $|N_i\rangle$. Let us arrange the eigenvalues h_n of $\hat{H}$ in increasing order $h_0 \leq h_1 \leq \ldots$ The corresponding eigenstates are of the type $|\tilde{h}_n\rangle = |N_1\rangle \otimes \ldots \otimes |N_m\rangle$. Call h_k^A (h_l^B) the eigenvalues of $\hat{H}_A$ ($\hat{H}_B$). Correspondingly, we have eigenstates of the type

$$|\tilde{h}_k^A\rangle = |N_1\rangle \otimes \ldots \otimes |N_{m_A}\rangle\,, \tag{17}$$

$$|\tilde{h}_l^B\rangle = |N_{1+m_A}\rangle \otimes \ldots \otimes |N_{m_A+m_B}\rangle\,. \tag{18}$$

It is clear that, given an arbitrary $|\tilde{h}_n\rangle$, we may always decompose it as $|\tilde{h}_n\rangle = |\tilde{h}_k^A\rangle \otimes |\tilde{h}_l^B\rangle$ for some pair of labels k and l. For this reason, any set of d states $\{|\tilde{h}_n\rangle\}$ for the m modes can certainly be represented by a tensor product of $d \times d$ states suitably chosen within the local sets $\{|\tilde{h}_k^A\rangle\}$ and $\{|\tilde{h}_l^B\rangle\}$. As a consequence, the support of a d-dimensional projector as in Eq. (7) is always contained in the support of a local $d \times d$ projector $P_d^{\otimes} = P_d^A \otimes P_d^B$, where

$$P_d^A := \sum_{k=0}^{d-1} |\tilde{h}_k^A\rangle\langle\tilde{h}_k^A|,\quad P_d^B := \sum_{l=0}^{d-1} |\tilde{h}_l^B\rangle\langle\tilde{h}_l^B|, \tag{19}$$

for some suitable choice of $\{|\tilde{h}_k^A\rangle\}$ and $\{|\tilde{h}_l^B\rangle\}$.

This implies that there always exists a local projector $P_d^{\otimes}$ for which we may write

$$\mathrm{Tr}(\rho_{\mathbf{ab}} P_d^{\otimes}) \geq \mathrm{Tr}(\rho_{\mathbf{ab}} P_d) \geq 1-\gamma,\quad \gamma = \frac{E}{\sqrt[2]{d}-1}, \tag{20}$$

where we have also used Lemma 1. Set $p := \mathrm{Tr}(\rho_{\mathbf{ab}} P_d)$ and $p' := \mathrm{Tr}(\rho_{\mathbf{ab}} P_d^{\otimes})$, so that we have truncated states

$$\delta_{\mathbf{ab}} = p^{-1} P_d \rho_{\mathbf{ab}} P_d,\quad \delta'_{\mathbf{ab}} = p'^{-1} P_d^{\otimes} \rho_{\mathbf{ab}} P_d^{\otimes}. \tag{21}$$

Because of the wider support of $P_d^{\otimes}$, it is easy to check that

$$\|\rho_{\mathbf{ab}} - \delta'_{\mathbf{ab}}\| \leq \|\rho_{\mathbf{ab}} - \delta_{\mathbf{ab}}\| \leq 2\sqrt{\gamma}, \tag{22}$$

where we have used Lemma 1 in the last inequality.

In order to construct the LOCC truncation channel, let us consider the local POVM $\Pi_{ij} := \Pi_i^{\mathbf{a}} \otimes \Pi_j^{\mathbf{b}}$ where

$$\Pi_0^{\mathbf{a(b)}} = P_d^{\mathbf{a(b)}},\quad \Pi_1^{\mathbf{a(b)}} = I^{\mathbf{a(b)}} - P_d^{\mathbf{a(b)}}. \tag{23}$$

The parties apply these projections and then they communicate their outcomes to each other, employing one bit of classical information for each one-way CC. If both parties project onto the local d-dimensional support then they apply an identity channel; if one of them projects outside this local support, they both apply a damping channel which maps any input into a fixed state within the support (which can always be chosen as the vacuum state).

More precisely, we define the LOCC truncation channel

$$\mathbb{T}_d^{\otimes}(\rho_{\mathbf{ab}}) = \sum_{i,j=0,1} \mathcal{E}_{ij}\left(\Pi_{ij}\rho_{\mathbf{ab}}\Pi_{ij}^{\dagger}\right), \tag{24}$$

where Π_{ij} is the local POVM defined above and

$$\mathcal{E}_{ij} = \begin{cases} \mathcal{I}_{\mathbf{a}} \otimes \mathcal{I}_{\mathbf{b}} & \text{for } i=j=0 \\ \mathcal{E}_{\mathbf{a}}^* \otimes \mathcal{E}_{\mathbf{b}}^* & \text{otherwise,} \end{cases} \tag{25}$$

where channel $\mathcal{E}_{\mathbf{a(b)}}^*$ provides an $m_{\mathbf{a}}$- ($m_{\mathbf{b}}$-) mode vacuum state for any input.

It is clear that the result is a truncated state $\tilde{\rho}_{\mathbf{ab}} := \mathbb{T}_d^{\otimes}(\rho_{\mathbf{ab}})$ where each set of modes $\mathbf{a}$ and $\mathbf{b}$ is supported in a d-dimensional Hilbert space. In particular, we have

$$\tilde{\rho}_{\mathbf{ab}} = p'\delta'_{\mathbf{ab}} + (1-p')\,|0\rangle_{\mathbf{ab}}\,\langle 0|\ . \tag{26}$$

Using the convexity of the trace norm, we get

$$D(\rho_{\mathbf{ab}}, \tilde{\rho}_{\mathbf{ab}}) = \frac{1}{2}\|\rho_{\mathbf{ab}} - \tilde{\rho}_{\mathbf{ab}}\| \leq \frac{p'}{2}\|\rho_{\mathbf{ab}} - \delta'_{\mathbf{ab}}\| + \frac{1-p'}{2}\|\rho_{\mathbf{ab}} - |0\rangle_{\mathbf{ab}}\langle 0|\| \leq p'\sqrt{\gamma} + 1 - p' \leq \sqrt{\gamma} + \gamma, \tag{27}$$

which conclude the proof. □

Finally, note that LOCC channels from DVs to CVs can be constructed by using hybrid quantum teleportation [3]. For instance, a polarisation qubit $\alpha\left|\uparrow\right\rangle_a + \beta\left|\downarrow\right\rangle_a$ can be teleported onto a single-rail qubit, which is the bosonic subspace spanned by the vacuum $|0\rangle_b$ and the single-photon state $|1\rangle_b$. It is sufficient to build a hyper-entangled Bell state $\left|\uparrow\right\rangle_{a'}|1\rangle_b + \left|\downarrow\right\rangle_{a'}|0\rangle_b$ and apply a discrete variable Bell detection on qubits a and a'. This teleports a onto the bosonic mode b, up to Pauli operators (suitably re-written in terms of the ladder operators) that can be undone from the output state. Such procedure can be readily extended to teleport qudits into bosonic modes in a LOCC fashion.

Supplementary Note 2. LOWER BOUND AT ANY DIMENSION

Coherent and reverse coherent information of a quantum channel

Consider a quantum channel $\mathcal{E}$ applied to some input state ρ_A of system A. Let us introduce the purification $|\psi\rangle_{RA}$ of ρ_A by means of an auxiliary system R. We can therefore consider the output $\rho_{RB} = \mathcal{I}\otimes\mathcal{E}(|\psi\rangle_{RA}\langle\psi|)$. By definition, the coherent information is [4, 5]

$$I_{\mathrm{C}}(\mathcal{E},\rho_A) = I(A\rangle B)_{\rho_{RB}} = S(\rho_B) - S(\rho_{RB}) \ , \tag{28}$$

where $\rho_B := \mathrm{Tr}_R(\rho_{RB})$ and $S(\rho) := -\mathrm{Tr}(\rho\log_2\rho)$ is the von Neumann entropy. Similarly, the reverse coherent information is given by [6, 7]

$$I_{\mathrm{RC}}(\mathcal{E},\rho_A) = I(A\langle B)_{\rho_{RB}} = S(\rho_R) - S(\rho_{RB}) \ , \tag{29}$$

where $\rho_R := \mathrm{Tr}_B(\rho_{RB})$.

When the input state ρ_A is a maximally-mixed state, its purification is a maximally-entangled state Φ_{RA}, so that ρ_{RB} is the Choi matrix of the channel, i.e., $\rho_{\mathcal{E}}$. We then define the coherent information of the channel as

$$I_{\mathrm{C}}(\mathcal{E}) = I(A\rangle B)_{\rho_{\mathcal{E}}} \ . \tag{30}$$

Similarly, its reverse coherent information is

$$I_{\mathrm{RC}}(\mathcal{E}) = I(A\langle B)_{\rho_{\mathcal{E}}} \ . \tag{31}$$

Note that for unital channels, i.e., channels preserving the identity $\mathcal{E}(I) = I$, we have $I_{\mathrm{C}}(\mathcal{E}) = I_{\mathrm{RC}}(\mathcal{E})$. This is just a consequence of the fact that, the reduced states ρ_A and ρ_R of a maximally entangled state Φ_{RA} is a maximally-mixed state I/d, where d is the dimension of the Hilbert space (including the limit for $d \to +\infty$). If the channel is unital, also the reduced state $\rho_B = \mathcal{E}(\rho_A)$ is maximally-mixed. As a result, $S(\rho_B) = S(\rho_A) = S(\rho_R)$ and we may write $I_{\mathrm{C}}(\mathcal{E}) = I_{\mathrm{RC}}(\mathcal{E}) := I_{(\mathrm{R})\mathrm{C}}(\mathcal{E})$.

In the specific case of discrete-variable systems ($d < +\infty$), we have $S(\rho_R) = \log_2 d$ and therefore

$$I_{(\mathrm{R})\mathrm{C}}(\mathcal{E}) = \log_2 d - S(\rho_{\mathcal{E}}) \ . \tag{32}$$

In particular, for unital qubit channels ($d = 2$), one has

$$I_{(\mathrm{R})\mathrm{C}}(\mathcal{E}) = 1 - S(\rho_{\mathcal{E}}) \ . \tag{33}$$

The latter two formulas will be exploited to compute the coherent information of discrete-variable channels.

The coherent information is an achievable rate for *forward* one-way entanglement distillation. Similarly, the reverse coherent information is an achievable rate for *backward* one-way entanglement distillation (i.e., assisted by a single and final CC from Bob to Alice). In fact, thanks to the hashing inequality [8], we may write

$$\max\{I_{\mathrm{C}}(\mathcal{E}), I_{\mathrm{RC}}(\mathcal{E})\} = \max\{I(A\rangle B)_{\rho_{\mathcal{E}}}, I(A\langle B)_{\rho_{\mathcal{E}}}\} \leq D_1(\rho_{\mathcal{E}}). \tag{34}$$

Hashing inequality in infinite dimension

The hashing inequality is known to be valid for finite-dimensional quantum systems. It is easy to extend this inequality to energy-constrained bosonic states by exploiting the continuity of the (reverse) coherent information in

the limit of infinite dimension. Consider the state ρ_{AB} of two bosonic modes, each mode having $\leq \bar{n}$ mean photons. Then, we may apply a projector P_d generating a d-dimensional truncated state δ_{AB} such that (see Lemma 1)

$$D(\rho_{AB}, \delta_{AB}) \leq \sqrt{\gamma}, \quad \gamma = \frac{2\bar{n}}{\sqrt{d}-1}. \tag{35}$$

According to ref. [9, Lemma 17], the trace-distance condition $D(\rho, \delta) \leq \sqrt{\gamma} < 1/6$ implies that the coherent information $I(A\rangle B) = -S(A|B)$ satisfies

$$|I(A\rangle B)_\rho - I(A\rangle B)_\delta| \leq 16\sqrt{\gamma}\log_2\left[\frac{2e(\bar{n}+1)}{1-\sqrt{\gamma}}\right] + 32H_2(3\sqrt{\gamma}) \ , \tag{36}$$

where H_2 the binary Shannon entropy

$$H_2(p) := -p\log_2 p - (1-p)\log_2(1-p). \tag{37}$$

For any $\bar{n}$, the limit $d \rightarrow +\infty$ implies that $\gamma \rightarrow 0$ and therefore

$$|I(A\rangle B)_\rho - I(A\rangle B)_\delta| \rightarrow 0 \ . \tag{38}$$

An equivalent result holds for the reverse coherent information $I(A\langle B) = -S(B|A)$.

Thus for any $\bar{n}$, the coherent and reverse coherent information are continuous in the limit of infinite dimension. This means that the hashing inequality [8] is extended to bosonic systems with constrained energy. In other words, $I(A\rangle B)_\rho$ ($I(A\langle B)_\rho$) represents an achievable rate for the distillable entanglement of the energy-bounded bosonic state ρ via forward (backward) CCs.

Extension to energy-unbounded Choi matrices of bosonic Gaussian channels

For bosonic systems, the ideal EPR state Φ is defined as the limit of two-mode squeezed vacuum (TMSV) states Φ^μ, where $\mu = \bar{n} + 1/2$ is sent to infinity (here $\bar{n}$ is the mean photon number in each mode) [10]. Thus, the Choi matrix of a Gaussian channel is defined as the asymptotic operator $\rho_{\mathcal{E}} := \lim_\mu \rho^\mu_{\mathcal{E}}$ where $\rho^\mu_{\mathcal{E}} := \mathcal{I} \otimes \mathcal{E}(\Phi^\mu)$. Correspondingly, the computation of the (reverse) coherent information of the channel is performed as a limit, i.e., we have

$$I_{\mathrm{C}}(\mathcal{E}) = I(A\rangle B)_{\rho_{\mathcal{E}}} := \lim_\mu I(A\rangle B)_{\rho^\mu_{\mathcal{E}}} \ , \tag{39}$$

$$I_{\mathrm{RC}}(\mathcal{E}) = I(A\langle B)_{\rho_{\mathcal{E}}} := \lim_\mu I(A\langle B)_{\rho^\mu_{\mathcal{E}}} \ . \tag{40}$$

As we will see afterwards in the technical derivations of Supplementary Note 4, for bosonic Gaussian channels the functionals $I(A\rangle B)_{\rho^\mu_{\mathcal{E}}}$ and $I(A\langle B)_{\rho^\mu_{\mathcal{E}}}$ are continuous, monotonic and bounded in μ. Therefore, the previous limits are finite and we can continuously extend the hashing inequality of Eq. (34) to the asymptotic Choi matrix $\rho_{\mathcal{E}}$ of a Gaussian channel, for which we may set $D_1(\rho_{\mathcal{E}}) := \lim_\mu D_1(\rho^\mu_{\mathcal{E}})$.

Supplementary Note 3. UPPER BOUND AT ANY DIMENSION

We provide alternate proofs of the weak converse theorem (Theorem 1 in the main paper). The first proof relies on an exponential growth for the total dimension of the private state [11–13] (which is justified by well-known arguments [14, 15]). The second proof relies on an exponential growth for the total energy. Finally, the third proof does not have any of the previous assumptions; in particular, it only depends on the "key part" of the private state. The first and third proofs are first given for DV channels and then extended to CV channels by means of truncation arguments (see Supplementary Note 1 for full details). The second proof simultaneously applies to both DV and CV channels, by means of embedding arguments. Besides truncation and embedding, the other main ingredients are basic properties of the trace norm and the relative entropy of entanglement (REE) [16], the "asymptotic continuity" of the REE [17, 18], and the REE upper bound for the distillable key of a quantum state [11, 12].

First proof of the weak converse theorem

Let us start by assuming that the output state $\rho^n_{\mathbf{ab}}$ in Alice and Bob's registers has total finite dimension $d_{\mathbf{ab}}$. Given $\rho^n_{\mathbf{ab}}$ and ϕ_n such that $\|\rho^n_{\mathbf{ab}} - \phi_n\| \leq \varepsilon \leq 1/3$, we may write the Fannes-type inequality [17]

$$E_{\mathrm{R}}(\phi_n) \leq E_{\mathrm{R}}(\rho^n_{\mathbf{ab}}) + 2\varepsilon \log_2 d_{\mathbf{ab}} + f(\varepsilon) \ , \tag{41}$$

where $f(\varepsilon) := 4\varepsilon - 2\varepsilon \log_2 \varepsilon$. This result is also known as asymptotic continuity of the REE. An alternate version states that $\|\rho^n_{\mathbf{ab}} - \phi_n\| \leq \varepsilon \leq 1/2$ implies [18]

$$E_{\mathrm{R}}(\phi_n) \leq E_{\mathrm{R}}(\rho^n_{\mathbf{ab}}) + 4\varepsilon \log_2 d_{\mathbf{ab}} + 2H_2(\varepsilon) \ , \tag{42}$$

where H_2 is the binary Shannon entropy. Note that the total dimension $d_{\mathbf{ab}}$ of the output state may always be considered to be greater than or equal to the dimension d_{P} of the private state. The latter involves two key systems (with total dimension d^2_{K}) and a shield system (with total dimension d_{S}), so that $d_{\mathrm{P}} = d^2_{\mathrm{K}} d_{\mathrm{S}}$. The logarithm of the dimension d_{K} determines the key rate, while the extra dimension d_{S} is needed to shield the key and can be assumed to grow exponentially in n (see the next subsection "Private states and size of the shield system" for full details on this secondary technical issue).

According to ref. [11], we may write

$$E_{\mathrm{R}}(\phi_n) \geq K(\phi_n) = \log_2 d_{\mathrm{K}} := nR^{\varepsilon}_n, \tag{43}$$

where $K(\phi_n)$ is the distillable key of ϕ_n. Therefore, from Eq. (42), we find

$$R^{\varepsilon}_n \leq \frac{E_{\mathrm{R}}(\rho^n_{\mathbf{ab}}) + 4\varepsilon \log_2 d_{\mathbf{ab}} + 2H_2(\varepsilon)}{n} \ . \tag{44}$$

For some sufficiently high $\alpha \geq 2$, let us set

$$\log_2 d_{\mathbf{ab}} \leq \alpha n R^{\varepsilon}_n \ . \tag{45}$$

Then the previous inequality becomes

$$R^{\varepsilon}_n \leq \frac{E_{\mathrm{R}}(\rho^n_{\mathbf{ab}}) + 2H_2(\varepsilon)}{n(1 - 4\varepsilon\alpha)} \ . \tag{46}$$

Asymptotically in n, we therefore get

$$\lim_n R^{\varepsilon}_n \leq \frac{1}{1 - 4\varepsilon\alpha} \lim n^{-1} E_{\mathrm{R}}(\rho^n_{\mathbf{ab}}) \ . \tag{47}$$

For $\varepsilon \rightarrow 0$, we derive

$$\lim_n R_n \leq \lim n^{-1} E_{\mathrm{R}}(\rho^n_{\mathbf{ab}}) \ , \tag{48}$$

whose optimization over adaptive protocols leads to the following weak converse bound for the key generation capacity

$$K(\mathcal{E}) := \sup_{\mathcal{L}} \lim_n R_n \leq E^{\bigstar}_{\mathrm{R}}(\mathcal{E}) := \sup_{\mathcal{L}} \lim n^{-1} E_{\mathrm{R}}(\rho^n_{\mathbf{ab}}) \ . \tag{49}$$

When $\rho^n_{\mathbf{ab}}$ is a CV bosonic state, we may consider an LOCC truncation channel $T^{\otimes}$ which maps the state into a DV state $\tilde{\rho}^n_{\mathbf{ab}} = T^{\otimes}(\rho^n_{\mathbf{ab}})$ supported in a subspace with cut-off α, so that the effective dimension is $2^{\alpha n R^{\varepsilon}_n}$ as in Eq. (45). This CV-to-DV mapping is large enough to leave the private state ϕ_n invariant, i.e., $\phi_n = T^{\otimes}(\phi_n)$. Because $\|\tilde{\rho}^n_{\mathbf{ab}} - \phi_n\| \leq \|\rho^n_{\mathbf{ab}} - \phi_n\| \leq \varepsilon$, we can then repeat the previous derivation and write Eq. (49) for $\tilde{\rho}^n_{\mathbf{ab}}$. Then, we introduce the upper-bound $E_{\mathrm{R}}(\tilde{\rho}^n_{\mathbf{ab}}) \leq E_{\mathrm{R}}(\rho^n_{\mathbf{ab}})$, which derives from the monotonicity of the REE under trace-preserving LOCCs (such as $T^{\otimes}$). For clarity, this derivation can be broken down into the following steps

$$E_{\mathrm{R}}(\tilde{\rho}^n_{\mathbf{ab}}) \overset{(1)}{=} S(\tilde{\rho}^n_{\mathbf{ab}} || \tilde{\sigma}^{\mathrm{opt}}_s) \overset{(2)}{\leq} S(\tilde{\rho}^n_{\mathbf{ab}} || \sigma'_s) \overset{(3)}{\leq} S(\rho^n_{\mathbf{ab}} || \sigma^{\mathrm{opt}}_s) = E_{\mathrm{R}}(\rho^n_{\mathbf{ab}}) \ , \tag{50}$$

where (1) we use the optimal separable state $\tilde{\sigma}^{\mathrm{opt}}_s$ which is the closest to $\tilde{\rho}^n_{\mathbf{ab}}$ in terms of relative entropy; (2) we introduce the non-optimal separable state $\sigma'_s = T^{\otimes}(\sigma^{\mathrm{opt}}_s)$, where σ^{opt}_s is the separable state closest to $\rho^n_{\mathbf{ab}}$ (because $T^{\otimes}$ is a LOCC, it preserves the separability of input states); and (3) we exploit the fact that the relative entropy cannot increase under trace-preserving LOCCs, which holds in arbitrary dimension [16, 19]. Thus, we may write Eq. (49) where $E_{\mathrm{R}}(\rho^n_{\mathbf{ab}})$ is directly computed on the bosonic state $\rho^n_{\mathbf{ab}}$.

Private states and size of the shield system

Let us discuss here the secondary technical detail related with the size of the shield system which appears in the definition of a private state. Consider a finite dimensional system of dimension $d_{\rm K}$ and basis $\{|i\rangle\}_{i=0}^{d_{\rm K}-1}$. A private state between Alice and Bob can be written in the form [11, 12]

$$\phi_{ABA'B'} = U(\Phi_{AB} \otimes \chi_{A'B'})U^{\dagger}, \tag{51}$$

where AB is the total key system in the maximally entangled state

$$\Phi_{AB} = |\Phi\rangle_{AB} \langle\Phi| , \quad |\Phi\rangle_{AB} := d_{\rm K}^{-1/2} \sum_{i=0}^{d_{\rm K}-1} |i\rangle_A |i\rangle_B , \tag{52}$$

while $A'B'$ is the shield system in a state $\chi_{A'B'}$ protecting the key from eavesdropping. In Eq. (51), the unitary U is a controlled-unitary known as "twisting unitary" which takes the form [12]

$$U = \sum_{i,j=0}^{d_{\rm K}-1} |i\rangle_A \langle i| \otimes |j\rangle_B \langle j| \otimes U_{A'B'}^{ij}, \tag{53}$$

with $U_{A'B'}^{ij}$ being arbitrary unitary operators.

One can prove that a dilation of a private state into an environment E (owned by Eve) must take the form [12]

$$\phi_{ABA'B'E} = (U \otimes I_E)(\Phi_{AB} \otimes \chi_{A'B'E})(U \otimes I_E)^{\dagger}, \tag{54}$$

with $\chi_{A'B'} = \mathrm{Tr}_E(\chi_{A'B'E})$. Note that one can also equivalently write

$$\phi_{ABA'B'E} = d_{\rm K}^{-1} \sum_{i,j=0}^{d_{\rm K}-1} |ii\rangle_{AB} \langle jj| \otimes U_{A'B'}^{ii} \chi_{A'B'E} (U_{A'B'}^{jj})^{\dagger}. \tag{55}$$

By making local measurements on the key system AB and tracing out the shield system $A'B'$, Alice and Bob retrieve the ideal classical-classical-quantum (ccq) state [12]

$$\tau_{ABE} = d_{\rm K}^{-1} \sum_{i=0}^{d_{\rm K}-1} |i\rangle_A \langle i| \otimes |i\rangle_B \langle i| \otimes \tau_E, \tag{56}$$

with τ_E arbitrary. More precisely, one shows [12] that $\tau_E^i = \tau_E$ for any i in Eq. (56). The shared randomness in the final classical AB system provides $\log_2 d_{\rm K}$ secret-key bits. Thus, the dimension $d_{\rm K}$ of each key system defines the number of secret-key bits (i.e., the rate of the protocol), while the dimension $d_{\rm S}$ of the shield system can in principle be arbitrary. The total dimension of the private state is $d_{\rm P} = d_{\rm K}^2 d_{\rm S}$.

In a key distillation protocol, where Alice and Bob start from n shared copies $\rho_{AB}^{\otimes n}$ and apply LOCCs to approximate a private state, the size of the shield $d_{\rm S}$ grows with the number of classical bits exchanged in their CCs. In fact, Eve may store all these bits in her local register and a private state can be approximated by the parties only if the dimension of Eve's register is smaller than the dimension of the shield system. This is implied by Eq. (56) as explained in ref. [12, Section III].

Now we may ask: *Is the shield size $d_{\rm S}$ super-exponential in n?* The answer is *no* for DV systems.

This was originally proven in ref. [14] and also discussed in ref. [15]. This result also holds for key distribution through memoryless channels at any dimension (finite or infinite). Let us remark that, despite the proof may appear involved, it is actually a trivial modification of the one in ref. [14, Appendix A (arXiv v3 version)]. It is based on the fact that one can always design an approximate protocol where key distribution through n uses of a finite- or infinite-dimensional channel is broken down into m identical and independent n_0-long sub-protocols. These sub-protocols provide m copies, which are truncated, measured and whose shields may be discarded. The effective increase of the shield size will then come from one-way key distillation of these output copies, which has an exponential contribution in $m < n$. In the following, we report this adaptation (with all details) only for the sake of completeness.

Lemma 4 (Shield size. Trivially adapted from ref. [14]): Consider n uses of an adaptive key generation protocol through a quantum channel at any dimension (finite or infinite). Without loss of generality, we can assume that the effective dimension of the shield system d_S grows in such a way that $\liminf_n (d_S/c^n)$ is a constant for some $c \geq 1$.

Proof: Let us represent Alice's and Bob's local registers as $\mathbf{a} = AA'$ and $\mathbf{b} = BB'$, where A and B are the local key systems, while A' and B' are the local shield systems. Denote Eve's register by E. Even if all these systems are infinite-dimensional for bosonic channels, the key systems A and B of the private state $\phi^{n_0}_{AA'BB'E}$ have finite-dimensional support, as we can see from Eq. (54). Then consider an arbitrary adaptive key generation protocol $\mathcal{P}^n_{\text{key}}$, with key rate R and communication cost that is not necessarily linear in the number n of channel uses (this cost may even be singular, i.e., involving an infinite number of classical bits per channel use). For any $\varepsilon > 0$, there is a sufficiently large integer n_0 such that its output $\rho^{n_0}_{AA'BB'E}$ satisfies

$$\|\rho^{n_0}_{AA'BB'E} - \phi^{n_0}_{AA'BB'E}\| \leq \varepsilon, \tag{57}$$

where $\phi^{n_0}_{AA'BB'E}$ is a (dilated) private state with l_{n_0} secret bits such that

$$R_{n_0} := \frac{l_{n_0}}{n_0} \geq R - \varepsilon \ . \tag{58}$$

Assume that the restricted n_0-adaptive protocol is repeated m times, so that the total number of channel uses can be written as $n = n_0 m$. Correspondingly, Alice and Bob's output state will be equal to the tensor product $(\rho^{n_0}_{AA'BB'})^{\otimes m}$ with $\rho^{n_0}_{AA'BB'} = \text{Tr}_E(\rho^{n_0}_{AA'BB'E})$. Now, assume that the parties measure their key systems in the computational basis $|i\rangle_A \otimes |j\rangle_B$ while truncating any outcome outside the finite-dimensional $2^{l_{n_0}} \times 2^{l_{n_0}}$ support $\mathcal{S}_{\text{key}}$ of the private state's key system. They then discard their shield systems. This means that they apply the LOCC channel

$$\mathbb{L}^{\otimes}(\rho^{n_0}_{AA'BB'}) = \text{Tr}_{A'B'}\left[\sum_{i,j} \mathcal{E}_{ij}\left(\Pi_{ij}\rho^{n_0}_{AA'BB'}\Pi^{\dagger}_{ij}\right)\right], \tag{59}$$

where $\Pi_{ij} := \Pi^A_i \otimes \Pi^B_j$ projects onto the local computational bases, while the conditional channel $\mathcal{E}_{ij}$ is

$$\mathcal{E}_{ij} = \begin{cases} \mathcal{I}_{AB} & \text{for } i,j \in [0, 2^{l_{n_0}} - 1] \\ \mathcal{E}^A_e \otimes \mathcal{E}^B_e & \text{otherwise,} \end{cases} \tag{60}$$

where $\mathcal{E}_e$ is a map replacing any input with an erasure state $|e\rangle$ orthogonal to the support $\mathcal{S}_{\text{key}}$. (Note that the contribution of the extra dimension of $|e\rangle$ to the output state is completely negligible since $2^{l_{n_0}}$ is very large).

The action of $\mathbb{L}^{\otimes}$ on the (dilated) output state $\rho^{n_0}_{AA'BB'E}$ is such that we achieve a truncated ccq state $\tilde{\rho}^{n_0}_{ABE} := (\mathbb{L}^{\otimes} \otimes \mathcal{I}_E)(\rho^{n_0}_{AA'BB'E})$, where the key systems A and B are classical and finite-dimensional. Similarly, the action on the (dilated) private state provides the ideal ccq target state $\tau^{n_0}_{ABE} := (\mathbb{L}^{\otimes} \otimes I_E)(\phi^{n_0}_{AA'BB'E})$ which corresponds to Eq. (56) with $\log_2 d_K = l_{n_0}$. Also note that this classicalization and truncation step just needs two bits of CC to be implemented: These bits are needed to identify those instances where the measurement of the other party falls outside the support (this classical overhead is clearly linear in the number m of blocks).

All the procedure is a trivial modification of the one in ref. [14, Appendix A (arXiv v3 version)]. Here we implement it in a coherent way and we include CV systems, for which $\mathbb{L}^{\otimes}$ measures the key systems within the finite-dimensional support of the private state, while collapsing any contribution from the remaining part of the infinite-dimensional Hilbert space. It is easy to check that $\mathbb{L}^{\otimes}$ can also be implemented in two subsequent steps: First a truncation channel into a $(2^{l_{n_0}} + 1) \times (2^{l_{n_0}} + 1)$ subspace and then a measurement channel in the computational bases.

Using the monotonicity of the trace distance under channels (at any dimension), from Eq. (57) we may write

$$\|\tilde{\rho}^{n_0}_{ABE} - \tau^{n_0}_{ABE}\| \leq \varepsilon. \tag{61}$$

Let us consider the reduced state $\tilde{\rho}^{n_0}_{AB} = \text{Tr}_E(\tilde{\rho}^{n_0}_{ABE})$. Given many copies $(\tilde{\rho}^{n_0}_{AB})^{\otimes m}$, Alice and Bob may apply one-way key distillation at an achievable rate (secret bits per block) given by the Devetak-Winter (DW) rate [8]

$$R^{\text{DW}}_{\tilde{\rho}} := I(A:B) - I(A:E) = S(A|E) - S(A|B), \tag{62}$$

where $I(:)$ is the quantum mutual information (equal to the classical mutual information on classical systems A and B), and $S(|)$ is the conditional von Neumann entropy, with all quantities being computed on the extended output $\tilde{\rho} := \tilde{\rho}^{n_0}_{ABE}$. Note that the DW rates are achievable rates at any dimension (finite or infinite).

Let us set $\tau := \tau_{ABE}^{n_0}$. We then compute the difference

$$R_{\tau}^{\mathrm{DW}} - R_{\tilde{\rho}}^{\mathrm{DW}} \leq |S(A|E)_{\tau} - S(A|E)_{\tilde{\rho}}| + |S(A|B)_{\tilde{\rho}} - S(A|B)_{\tau}| \leq 8\varepsilon \log_2 \dim \mathcal{H}_A + 4H_2(\varepsilon), \tag{63}$$

where H_2 is the binary Shannon entropy. In Eq. (63), we have used the Alicki-Fannes' inequality for the conditional quantum entropy [20] which is valid for any $||\tilde{\rho} - \tau|| \leq \varepsilon < 1$ as in Eq. (61).

Note that Eq. (63) only contains the dimension of Alice's Hilbert space $\mathcal{H}_A$ (truncated in each sub-protocol), while the Hilbert spaces of Bob and Eve do not have any restriction of their dimensionality. Because $R_{\tau}^{\mathrm{DW}} = l_{n_0}$ and $\dim \mathcal{H}_A = 2^{l_{n_0}}$, we may then write

$$R_{\tilde{\rho}}^{\mathrm{DW}} \geq (1 - 8\varepsilon) l_{n_0} - 4H_2(\varepsilon), \tag{64}$$

exactly as in ref. [14, Appendix A (arXiv v3 version)]. Therefore, by dividing the latter equation by n_0, one gets the average rate (per channel use)

$$\tilde{R} := \frac{1}{n_0} R_{\tilde{\rho}}^{\mathrm{DW}} \geq (1 - 8\varepsilon)(R - \varepsilon) - \frac{4H_2(\varepsilon)}{n_0}. \tag{65}$$

It is now important to note that Alice and Bob can achieve the average DW rate $\tilde{R}$ using an amount of one-way CC which is linear in the block number $m < n$. In fact, the communication cost (bits per block) associated with the one-way key distillation of Alice and Bob's copies $(\tilde{\rho}_{AB}^{n_0})^{\otimes m}$ is equal to the conditional (Shannon) entropy $S(A|B)$ between the two classical finite-dimensional systems A and B [8]. This overhead is bounded by $\log_2 \dim \mathcal{H}_{A,B} = l_{n_0}$ classical bits per block, so that it scales at most linearly as $m l_{n_0}$. Therefore, by decreasing ε, we get a sequence of protocols whose classical communication scales linearly in m while their rates approach R according to Eq. (65). Correspondingly, the size of the shield grows at most exponentially in m.

Let us take a closer look at the dynamics of the shield. Within each block, the shield size may increase super-exponentially (even to infinite) but then this size collapses to zero at the end of each block (after n_0 uses) once the parties have generated their finite-dimensional cc-state $\tilde{\rho}_{AB}^{n_0}$. For this reason, there is no surviving contribution to shield coming from the m sub-protocols. The only contribution to the shield size is that (exponential) coming from the protocol of one-way key distillation on the output finite-dimensional copies. Thus, at values of $n = n_0 m$ for integer m, the dimension d_{S} scales as an exponential function, i.e., it is bounded by c^n for some $c \geq 1$. If we look at the shield dynamics for every n, then we may always replace the limit by an inferior limit, i.e., we may always say that $\liminf_n (d_{\mathrm{S}}/c^n)$ is a constant (with the infimum reached by the sequence of points $n = n_0 m$). $\square$

Second proof of the weak converse theorem

This second proof simultaneously applies to DV and CV systems, and relies on the physical assumption that the energy of the output state grows at most exponentially in the number of channel uses. Consider bosonic modes, since any DV system can be unitarily embedded into a CV system (operation which does not change the trace distance). In general, we assume $m_{\mathbf{a}}$ modes at Alice's side and $m_{\mathbf{b}}$ modes at Bob's side (recall that the parties' local registers may be composed of a countable set of quantum systems). Assume that the output state $\rho_{\mathbf{ab}}^n$ and the target state $\phi_{\mathbf{ab}}^n$ have mean photon numbers bounded by E_n, where we may set $E_n \leq 2^{cn}$ for some constant c.

Let us apply a LOCC truncation channel $\mathbb{T}_d^{\otimes}$, local with respect to Alice and Bob's bipartition of modes $m_{\mathbf{a}} + m_{\mathbf{b}}$, which truncates Alice's and Bob's local Hilbert spaces to finite dimension $d = E_n^4$ (other choices are possible). This means that the truncated states $\rho_{\mathbf{ab}}^{n,d} := \mathbb{T}_d^{\otimes}(\rho_{\mathbf{ab}}^n)$ and $\phi_{\mathbf{ab}}^{n,d} := \mathbb{T}_d^{\otimes}(\phi_{\mathbf{ab}}^n)$ satisfies (see Lemma 3)

$$||\rho_{\mathbf{ab}}^{n,d} - \rho_{\mathbf{ab}}^n||, ||\phi_{\mathbf{ab}}^{n,d} - \phi_{\mathbf{ab}}^n|| \leq 2(\sqrt{\gamma} + \gamma), \quad \gamma = \frac{E_n}{E_n^2 - 1}. \tag{66}$$

Because $\|\rho_{\mathbf{ab}}^n - \phi_{\mathbf{ab}}^n\| \leq \varepsilon$, we can apply the triangle inequality and find

$$||\rho_{\mathbf{ab}}^{n,d} - \phi_{\mathbf{ab}}^{n,d}|| \leq \varepsilon + \varepsilon', \quad \varepsilon' := 4(\sqrt{\gamma} + \gamma) = O(E_n^{-1/2}). \tag{67}$$

Now the asymptotic continuity of the REE [18] leads to

$$E_{\mathrm{R}}(\phi_{\mathbf{ab}}^{n,d}) \leq E_{\mathrm{R}}(\rho_{\mathbf{ab}}^{n,d}) + 32(\varepsilon + \varepsilon') \log_2 E_n + 2H_2(\varepsilon + \varepsilon'), \tag{68}$$

where we use the fact that the total dimension of the truncated states is $d_{\mathbf{ab}} = d^2 = E_n^8$.

In Eq. (68) we may replace $E_{\rm R}(\rho^{n,d}_{\mathbf{ab}}) \leq E_{\rm R}(\rho^{n}_{\mathbf{ab}})$ due to the fact that the REE is monotonic under $\mathbb{T}^{\otimes}_d$ and invariant under embedding local unitaries. We may also replace $\log_2 E_n \leq cn$ and $E_{\rm R}(\phi^{n,d}_{\mathbf{ab}}) \geq K(\phi^{n,d}_{\mathbf{ab}}) = nR^{\varepsilon}_n(E_n)$, where the energy-constrained key rate must satisfy $\lim_n R^{\varepsilon}_n(E_n) = \lim_n R^{\varepsilon}_n$, with R^{ε}_n being the (finite) key rate associated with $\phi^{n}_{\mathbf{ab}}$. Therefore, we may write

$$nR^{\varepsilon}_n(E_n) \leq E_{\rm R}(\rho^{n}_{\mathbf{ab}}) + 32(\varepsilon+\varepsilon')cn + 2H_2(\varepsilon+\varepsilon'). \tag{69}$$

Diving by n and taking the limit for $n \to +\infty$, we get

$$\lim_n R^{\varepsilon}_n \leq \lim_n n^{-1} E_{\rm R}(\rho^{n}_{\mathbf{ab}}) + 32\varepsilon c. \tag{70}$$

Finally, by taking the limit of $\varepsilon \to 0$, we find

$$\lim_n R_n \leq \lim_n n^{-1} E_{\rm R}(\rho^{n}_{\mathbf{ab}}) \ , \tag{71}$$

which gives the final result $K(\mathcal{E}) \leq E^{\bigstar}_{\rm R}(\mathcal{E})$ by optimizing over all adaptive protocols.

Third proof of the weak converse theorem

Let us now give a final proof which is completely independent from the dimensionality of the shield system in the private state. We start from the DV case and then we prove the CV case by resorting to truncation arguments. After n uses of a DV quantum channel $\mathcal{E}$, an adaptive key-generation protocol has an output $\rho^{n}_{\mathbf{ab}} = \rho_{\mathbf{ab}}(\mathcal{E}^{\otimes n})$ such that

$$\|\rho^{n}_{\mathbf{ab}} - \phi^{n}_{\mathbf{ab}}\| \leq \varepsilon, \tag{72}$$

where $\phi^{n}_{\mathbf{ab}}$ is a private state. Let us write the local registers as $\mathbf{a} = AA'$ and $\mathbf{b} = BB'$, with AB being the key part (with dimension $d_{\rm K} \times d_{\rm K}$) and $A'B'$ being the shield. By definition of private state, we have

$$\phi^{n}_{\mathbf{ab}} = \phi^{n}_{ABA'B'} = U(\Phi^{n}_{AB} \otimes \chi_{A'B'})U^{\dagger}, \tag{73}$$

where U is a twisting unitary, $\chi_{A'B'}$ is a state of the shield, and Φ^{n}_{AB} is a Bell state with $\log d_{\rm K} = nR^{\varepsilon}_n$ secret bits.

Let us "untwist" the output state $\rho^{n}_{\mathbf{ab}} = \rho^{n}_{ABA'B'}$ and then take the partial trace over the shield system $A'B'$. This means to consider

$$\rho^{n}_{AB} = {\rm Tr}_{A'B'}\left(U^{\dagger}\rho^{n}_{ABA'B'}U\right) := \mathcal{W}(\rho^{n}_{ABA'B'}). \tag{74}$$

Trace norm is non-decreasing under partial trace and invariant under unitaries, so that Eq. (72) implies

$$\|\rho^{n}_{AB} - \Phi^{n}_{AB}\| \leq \varepsilon. \tag{75}$$

Following ref. [12], let us consider the set T of bipartite states σ_{AB} which are defined by $\sigma_{AB} = \mathcal{W}(\sigma_{ABA'B'})$ where $\sigma_{ABA'B'}$ is an arbitrary separable state (with respect to Alice and Bob's bipartition AA' and BB'). One may define the relative entropy distance from this set as

$$E^{T}_{\rm R}(\rho) = \inf_{\sigma \in T} S(\rho||\sigma) \ . \tag{76}$$

Because the set T is compact, convex and contains the maximally mixed state [12], this distance is asymptotically continuous, i.e., the condition $\|\rho_1 - \rho_2\| \leq \varepsilon < 1/2$ implies [18]

$$\left|E^{T}_{\rm R}(\rho_1) - E^{T}_{\rm R}(\rho_2)\right| \leq 4\varepsilon \log_2 d + 2H_2(\varepsilon) \ , \tag{77}$$

where d is the total dimension of the Hilbert space and H_2 is the binary Shannon entropy.

By applying this property to Eq. (75) with $d_{AB} = d^2_{\rm K}$, we then get

$$E^{T}_{\rm R}(\Phi^{n}_{AB}) \leq E^{T}_{\rm R}(\rho^{n}_{AB}) + 8\varepsilon \log_2 d_{\rm K} + 2H_2(\varepsilon). \tag{78}$$

Now we exploit two observations. The first is that

$$E^{T}_{\rm R}(\Phi^{n}_{AB}) \geq \log d_{\rm K} = nR^{\varepsilon}_n \ , \tag{79}$$

as shown in ref. [12, Lemma 7]. Then, we also have

$$E_{\mathrm{R}}^{T}(\rho_{AB}^{n}) \leq E_{\mathrm{R}}(\rho_{ABA'B'}^{n}) := E_{\mathrm{R}}(\rho_{\mathbf{ab}}^{n}) \ . \tag{80}$$

In fact, this is proven by the following chain of (in)equalities

$$E_{\mathrm{R}}^{T}(\rho_{AB}^{n}) \overset{(1)}{\leq} S[\rho_{AB}^{n}||\mathcal{W}(\sigma_{ABA'B'})] \overset{(2)}{=} S[\mathcal{W}(\rho_{ABA'B'}^{n})||\mathcal{W}(\sigma_{ABA'B'})] \overset{(3)}{\leq} S(\rho_{ABA'B'}^{n}|\sigma_{ABA'B'}) \overset{(4)}{=} E_{\mathrm{R}}(\rho_{ABA'B'}^{n}), \tag{81}$$

where (1) we use some arbitrary state $\sigma_{AB} \in T$, (2) we use Eq. (74), (3) we use the fact that the relative entropy is monotonic under partial trace and invariant under unitaries, and finally (4) we may always choose the separable state $\sigma_{ABA'B'}$ to be the one which is the closest to $\rho_{ABA'B'}^{n}$ in relative entropy (so that it defines its REE).

Using Eqs. (79) and (80) into Eq. (78), we find

$$nR_{n}^{\varepsilon} \leq E_{\mathrm{R}}(\rho_{\mathbf{ab}}^{n}) + 8\varepsilon \log_2 d_{\mathrm{K}} + 2H_2(\varepsilon). \tag{82}$$

Because $\log_2 d_{\mathrm{K}} = nR_{n}^{\varepsilon}$, this leads to

$$R_{n}^{\varepsilon} \leq \frac{E_{\mathrm{R}}(\rho_{\mathbf{ab}}^{n}) + 2H_2(\varepsilon)}{n(1-8\varepsilon)} \ , \tag{83}$$

so that, for large n, we may write

$$\lim_{n} R_{n}^{\varepsilon} \leq \frac{1}{1-8\varepsilon} \lim_{n} n^{-1} E_{\mathrm{R}}(\rho_{\mathbf{ab}}^{n}) \ . \tag{84}$$

By taking the limit of $\varepsilon \to 0$, we then find

$$\lim_{n} R_{n} \leq \lim_{n} n^{-1} E_{\mathrm{R}}(\rho_{\mathbf{ab}}^{n}). \tag{85}$$

Finally, by optimizing over all adaptive protocols $\mathcal{L}$, we establish the weak converse bound for the two-way key-generation capacity of the channel

$$K(\mathcal{E}) := \sup_{\mathcal{L}} \lim_{n} R_{n} \leq \sup_{\mathcal{L}} \lim_{n} n^{-1} E_{\mathrm{R}}(\rho_{\mathbf{ab}}^{n}) \ . \tag{86}$$

We now consider the CV case, i.e., a bosonic channel $\mathcal{E}$. In this case, the private state $\phi_{\mathbf{ab}}^{n} = \phi_{ABA'B'}^{n}$ of Eq. (73) is still built on a finite-dimensional $d_{\mathrm{K}} \times d_{\mathrm{K}}$ Bell state Φ_{AB}^{n} containing $\log d_{\mathrm{K}} = nR_{n}^{\varepsilon}$ secret bits. This Bell state may equivalently be thought to be embedded into a CV system where it is supported within a $d_{\mathrm{K}} \times d_{\mathrm{K}}$ subspace of the infinite-dimensional Hilbert space. The shield state $\chi_{A'B'}$ can be an arbitrary CV state and the twisting U is an arbitrary control-unitary as in Eq. (53) but where the target unitaries $U_{A'B'}^{ij}$ are defined on a CV state.

Let us apply an LOCC truncation channel $\mathbb{T}_{d_{\mathrm{K}}}^{\otimes}$ to the key systems A and B, so that their Hilbert spaces are truncated to finite dimension $d_{\mathrm{K}} \times d_{\mathrm{K}}$. Clearly, we have the invariance $\phi_{ABA'B'}^{n} = \mathbb{T}_{d_{\mathrm{K}}}^{\otimes} \otimes \mathcal{I}_{A'B'}(\phi_{ABA'B'}^{n})$, while we set $\rho_{ABA'B'}^{n,d_{\mathrm{K}}} := \mathbb{T}_{d_{\mathrm{K}}}^{\otimes} \otimes \mathcal{I}_{A'B'}(\rho_{ABA'B'}^{n})$, where $\mathcal{I}_{A'B'}$ is an identity channel acting on the shield systems. By using the monotonicity of the trace norm under channels, we may write

$$||\rho_{ABA'B'}^{n,d_{\mathrm{K}}} - \phi_{ABA'B'}^{n}|| \leq \varepsilon \ . \tag{87}$$

As before, let us define a channel $\mathcal{W}$ which untwists and partial-traces the states as in Eq. (74). This channel provides the $d_{\mathrm{K}} \times d_{\mathrm{K}}$ states $\tilde{\rho}_{AB}^{n} = \mathcal{W}(\rho_{ABA'B'}^{n,d_{\mathrm{K}}})$ and $\Phi_{AB}^{n} = \mathcal{W}(\phi_{ABA'B'}^{n})$, for which we may write (using monotonicity)

$$||\tilde{\rho}_{AB}^{n} - \Phi_{AB}^{n}|| \leq \varepsilon \ . \tag{88}$$

Consider now the set T of states defined by $\sigma_{AB} = \mathcal{W}(\sigma_{ABA'B'})$, where $\sigma_{ABA'B'}$ is an arbitrary separable state (with respect to the bipartition AA' and BB') where the key-part AB has dimension $d_{\mathrm{K}} \times d_{\mathrm{K}}$ while the shield-part $A'B'$ is infinite-dimensional. The set T is compact, convex and contains the maximally mixed state. Thus, the relative entropy distance $E_{\mathrm{R}}^{T}(\rho) = \inf_{\sigma \in T} S(\rho||\sigma)$ is asymptotically continuous. This means that we may write

$$E_{\mathrm{R}}^{T}(\Phi_{AB}^{n}) \leq E_{\mathrm{R}}^{T}(\tilde{\rho}_{AB}^{n}) + 8\varepsilon \log_2 d_{\mathrm{K}} + 2H_2(\varepsilon). \tag{89}$$

Now we derive

$$E_{\mathrm{R}}^{T}(\tilde{\rho}_{AB}^{n}) \overset{(1)}{\leq} E_{\mathrm{R}}(\rho_{ABA'B'}^{n,d_{\mathrm{K}}}) \overset{(2)}{\leq} E_{\mathrm{R}}(\rho_{ABA'B'}^{n}) := E_{\mathrm{R}}(\rho_{\mathbf{ab}}^{n}), \tag{90}$$

where (1) follows the derivation given in Eq. (81), and (2) comes from the monotonicity of the REE under $\mathbb{T}_{d_{\mathrm{K}}}^{\otimes} \otimes \mathcal{I}_{A'B'}$. By replacing Eqs. (79) and (90) into Eq (89), we find Eq. (82) where $\rho_{\mathbf{ab}}^{n}$ is a now a CV state. The remainder of the proof is the same as before.

Supplementary Note 4. TECHNICAL DERIVATIONS FOR BOSONIC GAUSSIAN CHANNELS

Basic tools for continuous variables

Let us consider n bosonic modes with quadrature operators $\hat{x} = (\hat{q}_1, \ldots, \hat{q}_n, \hat{p}_1, \ldots, \hat{p}_n)^T$ and canonical commutation relations [21]

$$[\hat{x}, \hat{x}^T] = i\Omega, \quad \Omega := \begin{pmatrix} 0 & 1 \\ -1 & 0 \end{pmatrix} \otimes I \ , \tag{91}$$

with I being the $n \times n$ identity matrix. An arbitrary multimode Gaussian state $\rho(u, V)$, with mean value u and covariance matrix (CM) V, can be written as [22]

$$\rho = \frac{\exp\left[-\frac{1}{2}(\hat{x}-u)^T G(\hat{x}-u)\right]}{\det\left(V + i\Omega/2\right)^{1/2}}, \tag{92}$$

where the Gibbs matrix G is specified by

$$G = 2i\Omega \, \coth^{-1}(2Vi\Omega). \tag{93}$$

The CM of a Gaussian state can be decomposed by using Williamson's theorem [10]. This provides the symplectic spectrum $\{\nu_1, \ldots, \nu_n\}$ which must satisfy the uncertainty principle $\nu_k \geq 1/2$. Similarly, we may write $\nu_k = \bar{n}_k + 1/2$ where $\bar{n}_k$ are thermal numbers, i.e., mean number of photons in each mode. The von Neumann entropy of a Gaussian state can be easily computed as

$$S(\rho) = \sum_k s(\nu_k) = \sum_k h(\bar{n}_k), \tag{94}$$

where

$$\begin{cases} s(\nu) := \left(\nu + \frac{1}{2}\right)\log_2\left(\nu + \frac{1}{2}\right) - \left(\nu - \frac{1}{2}\right)\log_2\left(\nu - \frac{1}{2}\right), \\ h(\bar{n}) := (\bar{n}+1)\log_2(\bar{n}+1) - \bar{n}\log_2 \bar{n}. \end{cases} \tag{95}$$

The most typical Gaussian state of two modes A and B is a two-mode squeezed thermal state. This has zero-mean and CM of the form

$$V = \begin{pmatrix} a & c \\ c & b \end{pmatrix} \oplus \begin{pmatrix} a & -c \\ -c & b \end{pmatrix} \ , \tag{96}$$

with arbitrary $a, b \geq 1/2$ and c satisfying the condition

$$c \leq c_{\max} := \min\left\{\sqrt{\left(a - \tfrac{1}{2}\right)\left(b + \tfrac{1}{2}\right)}, \sqrt{\left(a + \tfrac{1}{2}\right)\left(b - \tfrac{1}{2}\right)}\right\}. \tag{97}$$

These bona-fide conditions can be checked using the tools in Refs. [23, 24] adapted to our different notation. For a CM as in Eq. (96), separability corresponds to

$$c \leq c_{\text{sep}} := \sqrt{\left(a - \tfrac{1}{2}\right)\left(b - \tfrac{1}{2}\right)} \ . \tag{98}$$

Thus, at any fixed a and b, the maximally-correlated but still separable Gaussian state is given by imposing the boundary condition $c = c_{\text{sep}}$. It is easy to check that this state contains the maximum correlations among the separable states, e.g., as quantified by its (unrestricted, generally non-Gaussian) quantum discord [25].

For $c_{\text{sep}} < c \leq c_{\max}$ in Eq. (96), the Gaussian state is entangled. A specific case is the TMSV state Φ^μ with CM of the form

$$V^\mu = \begin{pmatrix} \mu & c \\ c & \mu \end{pmatrix} \oplus \begin{pmatrix} \mu & -c \\ -c & \mu \end{pmatrix}, \quad c := \sqrt{\mu^2 - 1/4}, \quad \mu \geq 1/2. \tag{99}$$

As already discussed, for $\mu \to \infty$, this state describes the asymptotic CV EPR state Φ, realizing the ideal EPR conditions $\hat{q}_A = \hat{q}_B$ and $\hat{p}_A = -\hat{p}_B$.

A Gaussian channel is a CPTP map which transforms Gaussian states into Gaussian states. Single-mode Gaussian channels can be greatly simplified by means of input-output unitaries. In fact, these can always be put in canonical form [10] whose general action on input quadratures $\hat{x} = (\hat{q}, \hat{p})^T$ is given by

$$\hat{x} \to T\hat{x} + N\hat{x}_E + z \ , \tag{100}$$

where T and N are diagonal matrices, E is an environmental mode with $\bar{n}$ mean photons, and z is a classical Gaussian variable, with zero mean and CM ξI where $\xi \geq 0$. All Gaussian channels are teleportation-covariant and, therefore, Choi-stretchable (with an asymptotic Choi matrix). Teleportation-covariance is given by the fact that any displacement of the input $\hat{x} \to \hat{x} + d_k$ is mapped into a displacement Td_k on the output.

Depending on the specific canonical form we have different expressions in Eq. (100). We have:

- The thermal-loss channel $\mathcal{E}_{\text{loss}}(\eta, \bar{n})$ with transmissivity $0 \leq \eta \leq 1$ and $\bar{n}$ thermal photons. This is described by

$$\hat{x} \to \sqrt{\eta}\hat{x} + \sqrt{1-\eta}\hat{x}_E \ . \tag{101}$$

  For $\bar{n} = 0$, the channel $\mathcal{E}_{\text{loss}}(\eta) := \mathcal{E}_{\text{loss}}(\eta, 0)$ is called pure-loss channel or just "lossy channel".

- The amplifier channel $\mathcal{E}_{\text{amp}}(\eta, \bar{n})$ with gain $\eta > 1$ and $\bar{n}$ thermal photons (in the main text we use the letter g for the gain). This corresponds to the transformation

$$\hat{x} \to \sqrt{\eta}\hat{x} + \sqrt{\eta - 1}\hat{x}_E \ . \tag{102}$$

  For $\bar{n} = 0$, the channel $\mathcal{E}_{\text{amp}}(\eta) := \mathcal{E}_{\text{amp}}(\eta, 0)$ is called "quantum-limited amplifier".

- The additive-noise Gaussian channel $\mathcal{E}_{\text{add}}(\xi)$, which simply corresponds to

$$\hat{x} \to \hat{x} + z. \tag{103}$$

- Finally, there are other secondary forms. One is the conjugate of the amplifier, which is described by $\hat{x} \to \sqrt{-\eta}Z\hat{x} + \sqrt{1-\eta}\hat{x}_E$, where $\eta < 0$ and $Z = \text{diag}(1, -1)$ is the reflection matrix. Then, other pathological forms [10]: The A_2-form, which is a 'half' depolarising channel and corresponds to $\hat{x} \to (\hat{q}, 0)^T + \hat{x}_E$; and the B_1-form, which is described by $\hat{x} \to \hat{x} + (0, \hat{p}_v)^T$ where v is the vacuum.

Coherent and reverse coherent information of a Gaussian channel

Here we discuss the computation of the (reverse) coherent information for the most important single-mode Gaussian channels, i.e., the thermal-loss channel, the amplifier channel and the additive-noise Gaussian channel. Compactly, their action on input quadratures is given by

$$\hat{x} \to \sqrt{\eta}\hat{x} + \sqrt{|1-\eta|}\hat{x}_E + z, \tag{104}$$

where $\eta \geq 0$ is the transmission (or gain), E is the environmental mode in a thermal state with $\bar{n}$ mean photons, and z is a classical Gaussian variable with CM $\xi I \geq 0$. The Choi matrix $\rho_{\mathcal{E}}$ of this Gaussian channel $\mathcal{E} = \mathcal{E}(\eta, \bar{n}, \xi)$ is defined as an asymptotic limit. At the input we consider a sequence of TMSV states Φ^μ with CM as in Eq. (99). Then, at the output, we get a sequence of finite-energy Gaussian states

$$\rho_{\mathcal{E}}^\mu := \mathcal{I} \otimes \mathcal{E}(\Phi^\mu), \tag{105}$$

whose limit defines $\rho_{\mathcal{E}} := \lim_\mu \rho_{\mathcal{E}}^\mu$. The quasi-Choi matrices $\rho_{\mathcal{E}}^\mu$ are zero-mean Gaussian states with CM

$$V^\mu(\eta, \bar{n}, \xi) = \begin{pmatrix} \mu & \gamma \\ \gamma & \beta \end{pmatrix} \oplus \begin{pmatrix} \mu & -\gamma \\ -\gamma & \beta \end{pmatrix}, \quad \beta := \eta\mu + |1-\eta| \left(\bar{n} + \frac{1}{2}\right) + \xi, \quad \gamma := \sqrt{\eta(\mu^2 - 1/4)}. \tag{106}$$

Let us consider the symplectic eigenvalues of the output CM in Eq. (106), which are given by [10]

$$\nu_\pm = \sqrt{\frac{\Delta \pm \sqrt{\Delta^2 - 4\det V^\mu}}{2}}, \quad \Delta := \mu^2 + \beta^2 - 2\gamma^2. \tag{107}$$

Using the formula of the von Neumann entropy for Gaussian states and the definitions of the coherent information $I_{\rm C}$ and reverse coherent information $I_{\rm RC}$, we may write

$$I_{\rm C}(\mathcal{E},\Phi^{\mu}) = I(A\rangle B)_{\rho^{\mu}_{\mathcal{E}}} = s(\beta) - s(\nu_-) - s(\nu_+), \quad I_{\rm RC}(\mathcal{E},\Phi^{\mu}) = I(A\langle B)_{\rho^{\mu}_{\mathcal{E}}} = s(\mu) - s(\nu_-) - s(\nu_+), \tag{108}$$

where function $s(\cdot)$ is given in Eq. (95).

It is easy to see that these quantities are continuous and increasing in μ, for any fixed values of η, $\bar{n}$ and ξ. For instance, for the lossy channel ($0 \leq \eta \leq 1$, $\bar{n} = \xi = 0$), we simply have

$$I(A\rangle B)_{\rho^{\mu}_{\mathcal{E}}} = s\left[\frac{1-\eta}{2} + \eta\mu\right] - s\left[\frac{\eta}{2} + (1-\eta)\mu\right], \quad I(A\langle B)_{\rho^{\mu}_{\mathcal{E}}} = s(\mu) - s\left[\frac{\eta}{2} + (1-\eta)\mu\right]. \tag{109}$$

Thus, the limit for $\mu \to +\infty$ in the expressions of Eq. (108) is regular and finite. The asymptotic values represent the coherent and reverse coherent information of the considered Gaussian channels, i.e., we have

$$I_{\rm C}(\mathcal{E}) = I(A\rangle B)_{\rho_{\mathcal{E}}} := \lim_{\mu} I(A\rangle B)_{\rho^{\mu}_{\mathcal{E}}}, \quad I_{\rm RC}(\mathcal{E}) = I(A\langle B)_{\rho_{\mathcal{E}}} := \lim_{\mu} I(A\langle B)_{\rho^{\mu}_{\mathcal{E}}}, \tag{110}$$

as already defined in Eqs. (39) and (40). Correspondingly, the hashing inequality can be safely extended to the limit, i.e., from

$$\max\{I(A\rangle B)_{\rho^{\mu}_{\mathcal{E}}}, I(A\langle B)_{\rho^{\mu}_{\mathcal{E}}}\} \leq D_1(\rho^{\mu}_{\mathcal{E}}), \tag{111}$$

we may write

$$\max\{I_{\rm C}(\mathcal{E}), I_{\rm RC}(\mathcal{E})\} \leq D_1(\rho_{\mathcal{E}}) := \lim_{\mu} D_1(\rho^{\mu}_{\mathcal{E}}). \tag{112}$$

For the thermal-loss channel, the best lower bound is the reverse coherent information, given by [7]

$$I_{\rm RC}(\eta,\bar{n}) = -\log_2(1-\eta) - h(\bar{n}), \tag{113}$$

where $h(\cdot)$ is the entropic function defined in Eq. (95). In particular, for a lossy channel ($\bar{n} = 0$), one has

$$I_{\rm RC}(\eta) = -\log_2(1-\eta). \tag{114}$$

For the amplifier channel, the best lower bound is given by the coherent information, which is equal to [7, 26]

$$I_{\rm C}(\eta,\bar{n}) = \log_2\left(\frac{\eta}{\eta-1}\right) - h(\bar{n}), \tag{115}$$

and becomes

$$I_{\rm C}(\eta) = \log_2\left(\frac{\eta}{\eta-1}\right), \tag{116}$$

for the quantum-limited amplifier ($\bar{n} = 0$). The coherent information and reverse coherent information of the additive-noise Gaussian channel coincide. We have [26]

$$I_{\rm C}(\xi) = I_{\rm RC}(\xi) = -\log_2\xi - \frac{1}{\ln 2}. \tag{117}$$

Due to the hashing inequality, the quantities $I_{\rm C}(\mathcal{E})$ and $I_{\rm RC}(\mathcal{E})$ are achievable rates for one-way entanglement distillation. Therefore, they also represent achievable rates for key generation, just because an ebit is a particular type of secret bit. In particular, ref. [7] proved that $I_{\rm RC}(\mathcal{E})$ is an achievable lower bound for quantum key distribution (QKD) through a Gaussian channel without the need of preliminary entanglement distillation. In fact, $I_{\rm RC}(\mathcal{E})$ can be computed as the asymptotic key rate of a coherent protocol where:

(i) Alice prepares TMSV states $\Phi^{\mu}_{AA'}$ sending A' to Bob;

(ii) Bob heterodynes each output mode B and sends final CCs back to Alice;

(iii) Alice measures all her modes A by means of an optimal coherent detection that reaches the Holevo bound.

The achievable rate of this coherent protocol is given by a Devetak-Winter rate $R_{\rm DW}$ [8]. Because Eve holds the entire purification of Alice and Bob's Gaussian output state $\rho^{\mu}_{\mathcal{E}}$ and Bob's detections are rank-1 measurements, this rate is equal to the reverse coherent information [7] $R_{\rm DW} = I(A\langle B)_{\rho^{\mu}_{\mathcal{E}}}$ computed on Alice and Bob's output. Then, by taking the limit of $\mu \to +\infty$, one obtains $K(\mathcal{E}) \geq I_{\rm RC}(\mathcal{E})$.

How to compute the entanglement flux of a Gaussian channel

Here we discuss how to compute the entanglement flux of a single-mode Gaussian channel (in canonical form). We provide the general recipe and then we go into details of the specific channels in the next subsections. The entanglement flux of a Gaussian channel $\mathcal{E}$ satisfies

$$\Phi(\mathcal{E}) \leq \liminf_{\mu\to+\infty} S(\rho^{\mu}_{\mathcal{E}}||\tilde{\sigma}^{\mu}_{s}) , \tag{118}$$

where $\rho^{\mu}_{\mathcal{E}}$ is a sequence of quasi-Choi matrices as defined in Eq. (105) with CMs as in Eq. (106), while $\tilde{\sigma}^{\mu}_{s}$ is a suitable sequence of separable Gaussian states.

For any μ, we choose a separable Gaussian state $\tilde{\sigma}^{\mu}_{s}$ with CM $\tilde{V}^{\mu}(\eta,\bar{n},\xi)$ as in Eq. (106) but with the replacement

$$\gamma \to \sqrt{(\mu-1/2)(\beta-1/2)}, \tag{119}$$

for the off-diagonal term. At fixed marginals μ and β, this is the most-correlated separable Gaussian state that we can build according to Eqs. (96) and (98); it has maximum (non-Gaussian) discord [25] and minimizes the relative entropy $S(\rho^{\mu}_{\mathcal{E}}||\tilde{\sigma}^{\mu}_{s})$ as long as $\rho^{\mu}_{\mathcal{E}}$ is an entangled state. In the specific case where the channel $\mathcal{E}$ is entanglement-breaking, then $\rho^{\mu}_{\mathcal{E}}$ becomes separable and we can trivially pick $\tilde{\sigma}^{\mu}_{s}=\rho^{\mu}_{\mathcal{E}}$, which gives $S(\rho^{\mu}_{\mathcal{E}}||\tilde{\sigma}^{\mu}_{s})=0$.

In general, we are left with the analytical calculation of the relative entropy $S(\rho^{\mu}_{\mathcal{E}}||\tilde{\sigma}^{\mu}_{s})$ between two Gaussian states. This can be done in terms of their statistical moments according to our formula for the REE between two arbitrary multimode Gaussian states, which is given in the "Methods" section of our paper. For $S(\rho^{\mu}_{\mathcal{E}}||\tilde{\sigma}^{\mu}_{s})$ we find regular expressions with a well-defined limit, so that we can put $\liminf_{\mu}=\lim_{\mu}$ in Eq. (118). We provide full algebraic details below for the various Gaussian channels.

Entanglement flux of a thermal-loss channel

Consider a thermal-loss channel $\mathcal{E}_{\text{loss}}(\eta,\bar{n})$ with transmissivity $0\leq\eta\leq1$ and thermal number $\bar{n}$, so that thermal noise has variance $\omega=\bar{n}+1/2$. For $\bar{n}\geq\eta(1-\eta)^{-1}$, this channel is entanglement-breaking and we have $\Phi(\eta,\bar{n})=0$. For $\bar{n}<\eta(1-\eta)^{-1}$ we compute the relative entropy $S^{\mu}:=S\left(\rho^{\mu}_{\mathcal{E}}||\tilde{\sigma}^{\mu}_{s}\right)$ from the CMs $V^{\mu}(\eta\leq1,\bar{n},0)$ and $\tilde{V}^{\mu}(\eta\leq1,\bar{n},0)$ of the zero-mean Gaussian states $\rho^{\mu}_{\mathcal{E}}$ and $\tilde{\sigma}^{\mu}_{s}$. Using our formula for the relative entropy between Gaussian states, we get

$$S^{\mu}=-S_1+\frac{\Delta}{2\ln2}+\frac{1}{2}\log_2\left\{\frac{2\mu-1}{4}[2\omega-1+2\eta(\mu-\omega)]\right\}, \tag{120}$$

where S_1 is the contribution of the von Neumann entropy, while the other two terms come from the entropic functional $\Sigma(V^{\mu},\tilde{V}^{\mu},0)$ (see Methods for its definition). Term Δ is analytical but too cumbersome to be reported here.

By expanding for large μ, we may write

$$\Delta\to2\left[1-2\omega\coth^{-1}\left(\frac{1+\eta}{\eta-1}\right)\right]+O(\mu^{-1}),\quad S_1\to h(\bar{n})+\log_2\left[e(1-\eta)\mu\right]+O(\mu^{-1}), \tag{121}$$

and

$$\frac{1}{2}\log_2\left\{\frac{2\mu-1}{4}[2\omega-1+2\eta(\mu-\omega)]\right\}\to\log_2\mu\sqrt{\eta}+O(\mu^{-1}) . \tag{122}$$

Taking the limit $S^{\infty}=\liminf_{\mu}S^{\mu}=\lim_{\mu}S^{\mu}$, we get

$$S^{\infty}=-\log_2\left[(1-\eta)\eta^{\bar{n}}\right]-h(\bar{n}) . \tag{123}$$

As a result, by replacing in Eq. (118), we find that the entanglement flux of a thermal-loss channel $\mathcal{E}_{\text{loss}}(\eta,\bar{n})$ satisfies

$$\Phi(\eta,\bar{n})\leq\Phi_{\text{loss}}(\eta,\bar{n}):=\begin{cases}-\log_2\left[(1-\eta)\eta^{\bar{n}}\right]-h(\bar{n}) & \text{for } \bar{n}<\frac{\eta}{1-\eta},\\ 0 \quad \text{otherwise.}\end{cases} \tag{124}$$

The thermal bound in Eq. (124) is clearly tighter than previous bounds based on the squashed entanglement, such as the "Takeoka-Guha-Wilde" (TGW) thermal bound [27]

$$K_{\text{TGW}}=\log_2\left[\frac{(1-\eta)\bar{n}+1+\eta}{(1-\eta)\bar{n}+1-\eta}\right] , \tag{125}$$

and its improved version [28]. However, $\Phi_{\rm loss}$ does not generally coincide with the achievable lower-bound [7] given by the reverse coherent information of the channel [see Eq. (113)]. Thus, the generic two-way capacity of the thermal-loss channel satisfies the sandwich relation

$$-\log_2(1-\eta) - h(\bar{n}) \leq \mathcal{C}_{\rm loss}(\eta,\bar{n}) \leq \Phi_{\rm loss}(\eta,\bar{n}). \tag{126}$$

It is easy to check that, for a lossy channel ($\bar{n}=0$), the bounds Eq. (126) coincide, therefore establishing

$$\mathcal{C}_{\rm loss}(\eta) = -\log_2(1-\eta)\ . \tag{127}$$

Relation with quantum discord

The result of Eq. (127) sets the fundamental limit for secret-key generation, entanglement distribution and quantum communication in bosonic lossy channels. For high loss it provides the fundamental rate-loss scaling of 1.44η bits per channel use. This also coincides with the maximum discord that can be distributed to the parties in a single use of the channel. In fact, we may write the reverse coherent information of a (bosonic) channel $\mathcal{E}$ as [29] $I(A\langle B)_{\rho_\mathcal{E}} = D(B|A) - E_{\rm f}(B,E)$, where $D(B|A)$ is the quantum discord [30] of Alice and Bob's (asymptotic) Choi matrix $\rho_\mathcal{E}$, while $E_{\rm f}(B,E)$ is the entanglement of formation between Bob and Eve. Because a lossy channel $\mathcal{E}_{\rm loss} := \mathcal{E}_{\rm loss}(\eta,0)$ is dilated into a beamsplitter with a vacuum environment, we have $E_{\rm f}(B,E)=0$. Thus, for a lossy channel, we simultaneously have $I(A\langle B)_{\rho_{\mathcal{E}_{\rm loss}}} = D(B|A)$ and $\mathcal{C}_{\rm loss}(\eta) = I(A\langle B)_{\rho_{\mathcal{E}_{\rm loss}}}$. These relations lead to

$$\mathcal{C}_{\rm loss}(\eta) = D(B|A)\ , \tag{128}$$

where $D(B|A)$ is the quantum discord of the (asymptotic) Gaussian Choi matrix $\rho_{\mathcal{E}_{\rm loss}}$ [25]. In particular, this discord can be computed as Gaussian discord [31, 32].

Full calculation details for the lossy channel

For the sake of completeness, we provide the specific details of the computation of the relative entropy S^μ for the specific case of a lossy channel. After some algebra, we achieve

$$S^\mu = \log_2\left[\left(\mu - \frac{1}{2}\right)\sqrt{\eta}\right] - s\left[(1-\eta)\mu + \frac{\eta}{2}\right] + \frac{\Delta}{2\ln 2}\ , \tag{129}$$

where

$$\Delta := \frac{c-(2\mu-1)(1-\eta)a}{b}\coth^{-1}\left[\frac{(1-\eta)(1-2\mu)-a}{2}\right] - \frac{c+(2\mu-1)(1-\eta)a}{b}\coth^{-1}\left[\frac{(1-\eta)(1-2\mu)+a}{2}\right], \tag{130}$$

and

$$a := \sqrt{1-(6-\eta)\eta + 4\mu[1+(4-\eta)\eta + (1-\eta)^2\mu]}, \tag{131}$$

$$b := \sqrt{8\mu + (2\mu-1)[4\eta + (2\mu-1)(1-\eta)^2]}, \tag{132}$$

$$c := 2\eta(2\mu-1)\left(2\sqrt{4\mu^2-1} - 1 - 2\mu\right) - \eta^2(2\mu-1)^2 - (1+2\mu)^2. \tag{133}$$

We now insert the expression of Δ in Eq. (129) and we take the limit for $\mu\rightarrow+\infty$. This limit is defined (i.e., $\liminf_\mu = \lim_\mu$) and we get

$$S^\infty = \lim_{\mu\rightarrow+\infty} S^\mu = -\log_2(1-\eta)\ . \tag{134}$$

We can show this limit step-by-step. First note that, for large ν, we have

$$s(\nu) \rightarrow \log_2 e\nu + O(\nu^{-1})\ . \tag{135}$$

Thus, in the limit of $\mu\rightarrow+\infty$, the first two terms in the RHS of Eq. (129) become

$$\log_2\left[\left(\mu-\frac{1}{2}\right)\sqrt{\eta}\right] \rightarrow \log_2(\mu\sqrt{\eta}) + O(\mu^{-1}), \tag{136}$$

$$-s\left[(1-\eta)\mu + \frac{\eta}{2}\right] \rightarrow -\log_2[e(1-\eta)\mu] + O(\mu^{-1}). \tag{137}$$

Then, it is easy to show that, for $\mu \to +\infty$, we have

$$\begin{aligned}\Delta &\to \left[-4(1-\eta)\mu + O(\mu^0)\right]\coth^{-1}\left[-2(1-\eta)\mu + O(\mu^0)\right] - \left[-2 + O(\mu^{-1})\right]\coth^{-1}\left[\frac{1+\eta}{1-\eta} + O(\mu^{-1})\right] \\ &\to 2 - \ln\eta + O(\mu^{-1})\ . \end{aligned} \tag{138}$$

In conclusion, by using Eqs. (136), (137) and (138) into Eq. (129), we obtain the final result in Eq. (134).

Entanglement flux of a quantum amplifier

Consider an amplifier channel $\mathcal{E}_{\rm amp}(\eta,\bar{n})$ with gain $\eta > 1$ and thermal number $\bar{n}$, so that thermal noise has variance $\omega = \bar{n} + 1/2$. For $\bar{n} \geq (\eta-1)^{-1}$ this channel is entanglement breaking and therefore $\Phi(\eta,\bar{n}) = 0$. For $\bar{n} < (\eta-1)^{-1}$ we compute the relative entropy $S^{\mu} := S\left(\rho^{\mu}_{\mathcal{E}}||\tilde{\sigma}^{\mu}_{s}\right)$ from the CMs $V^{\mu}(\eta > 1,\bar{n},0)$ and $\tilde{V}^{\mu}(\eta > 1,\bar{n},0)$ of the zero-mean Gaussian states $\rho^{\mu}_{\mathcal{E}}$ and $\tilde{\sigma}^{\mu}_{s}$. Up to terms $O(\mu^{-1})$, we get

$$S(\rho^{\mu}_{\mathcal{E}}) \to h(\bar{n}) + \log_2 e(\eta-1)\mu, \quad -\operatorname{Tr}\left(\rho^{\mu}_{\mathcal{E}}\log_2\tilde{\sigma}^{\mu}_{s}\right) \to \frac{\ln(\eta\mu^2) + 2 + 4\omega\coth^{-1}\left(\frac{\eta+1}{\eta-1}\right)}{2\ln 2}. \tag{139}$$

For large μ we therefore obtain

$$S^{\infty} = \log_2\left(\frac{\eta^{\bar{n}+1}}{\eta-1}\right) - h(\bar{n}). \tag{140}$$

Thus we find

$$\Phi(\eta,\bar{n}) \leq \Phi_{\rm amp}(\eta,\bar{n}) := \begin{cases} \log_2\left(\dfrac{\eta^{\bar{n}+1}}{\eta-1}\right) - h(\bar{n}) & \text{for } \bar{n} < (\eta-1)^{-1}, \\ 0 \quad \text{otherwise}. \end{cases} \tag{141}$$

In general, $\Phi_{\rm amp}(\eta,\bar{n})$ does not coincide with the best known lower bound which is given by the coherent information of the channel in Eq. (115). Thus, the two-way capacity of a quantum amplifier channel satisfies

$$\log_2\left(\frac{\eta}{\eta-1}\right) - h(\bar{n}) \leq \mathcal{C}_{\rm amp}(\eta,\bar{n}) \leq \Phi_{\rm amp}(\eta,\bar{n}). \tag{142}$$

It is easy to check that, for the quantum-limited amplifier ($\bar{n} = 0$), the previous upper and lower bounds coincide, thus determining its two-way capacity

$$\mathcal{C}_{\rm amp}(\eta) = \log_2\left(\frac{\eta}{\eta-1}\right). \tag{143}$$

Thus, $\mathcal{C}_{\rm amp}(\eta)$ turns out to coincide with the unassisted quantum capacity $Q_{\rm amp}(\eta)$ [26, 33]. The result of Eq. (143) sets the fundamental limit for key generation, entanglement distribution and quantum communication with amplifiers. A trivial consequence is that infinite amplification is useless for communication since $\mathcal{C}_{\rm amp}(\infty) \to 0$. For an amplifier with typical gain 2, the maximum achievable rate for quantum communication is just 1 qubit per use.

Entanglement flux of an additive-noise Gaussian channel

Consider an additive-noise Gaussian channel $\mathcal{E}_{\rm add}(\xi)$ with noise variance $\xi \geq 0$. For $\xi \geq 1$ this channel is entanglement breaking and therefore we have $\Phi(\xi) = 0$. For $\xi < 1$ we compute the relative entropy $S^{\mu} := S\left(\rho^{\mu}_{\mathcal{E}}||\tilde{\sigma}^{\mu}_{s}\right)$ from the CMs $V^{\mu}(1,0,\xi)$ and $\tilde{V}^{\mu}(1,0,\xi)$ of the zero-mean Gaussian states $\rho^{\mu}_{\mathcal{E}}$ and $\tilde{\sigma}^{\mu}_{s}$. Discarding terms $O(\mu^{-1})$, we get

$$S(\rho^{\mu}_{\mathcal{E}}) \to \log_2(e^2\xi\mu), \quad -\operatorname{Tr}\left(\rho^{\mu}_{\mathcal{E}}\log_2\tilde{\sigma}^{\mu}_{s}\right) \to \frac{\ln\left[\frac{(2\mu-1)(2\xi+2\mu-1)}{4}\right] + 2(1+\xi)}{2\ln 2}. \tag{144}$$

which leads to

$$S^{\infty} = \liminf_{\mu} S^{\mu} = \lim_{\mu} S^{\mu} = \frac{\xi - 1}{\ln 2} - \log_2 \xi \ . \tag{145}$$

Thus we find

$$\Phi(\xi) \leq \Phi_{\text{add}}(\xi) := \begin{cases} \frac{\xi-1}{\ln 2} - \log_2 \xi & \text{for } \xi < 1, \\ \\ 0 & \text{otherwise.} \end{cases} \tag{146}$$

The best lower bound is its coherent information $I_{\text{C}}(\xi)$ of Eq. (117), so that the two-way capacity satisfies

$$-1/\ln 2 - \log_2 \xi \leq \mathcal{C}_{\text{add}}(\xi) \leq \Phi_{\text{add}}(\xi) \ . \tag{147}$$

It is interesting to note how quantum communication rapidly degrades when we compose quantum channels. For instance, a quantum-limited amplifier with gain 2 can transmit $Q_2 = 1$ qubit per use from Alice to Bob. This is the same amount which can be transmitted from Bob to Charlie, through a lossy channel with transmissivity 1/2. By using Bob as a quantum repeater, Alice can therefore transmit at least 1 qubit per use to Charlie. If we remove Bob and we compose the two channels, we obtain an additive-noise Gaussian channel with variance $\xi = 1/2$, which is limited to $Q_2 \lesssim 0.278$ qubits per use.

Secondary canonical forms

For the conjugate of the amplifier it is easy to check that this channel is always entanglement-breaking, so that it has zero flux and, therefore, zero two-way capacity $\mathcal{C} = 0$. The A_2-form [10], which is a 'half' depolarising channel, is also an entanglement-breaking channel, so that $\Phi = \mathcal{C} = 0$. Finally, for the "pathological" B_1-form [10], we find the trivial bound $\Phi = +\infty$.

Supplementary Note 5. TECHNICAL DERIVATIONS FOR DISCRETE-VARIABLE CHANNELS

Given a discrete-variable channel $\mathcal{E}$ in dimension d, we can easily derive its Choi matrix $\rho_{\mathcal{E}} = I \otimes \mathcal{E}(\Phi)$ from the maximally-entangled state

$$\Phi = \frac{1}{\sqrt{d}} \sum_{i=0}^{d-1} |ii\rangle, \tag{148}$$

where $\{|0\rangle, \dots, |i\rangle, \dots, |d-1\rangle\}$ is the computational basis of the qudit. We write the spectral decomposition

$$\rho_{\mathcal{E}} = \sum_k p_k |\varphi_k\rangle\langle\varphi_k|, \tag{149}$$

where $\mathbf{p} = \{p_k\}$ are the eigenvalues of the Choi matrix. The von Neumann entropy is simply equal to the Shannon entropy of the previous eigenvalues, i.e.,

$$S(\rho_{\mathcal{E}}) = H(\mathbf{p}) := -\sum_k p_k \log_2 p_k. \tag{150}$$

From the Choi matrix we may compute the coherent and reverse coherent information of the channel. In particular, for unital channels, these quantities coincide and are given by the simple formula in Eq. (32), i.e.,

$$I_{\text{C}}(\mathcal{E}) = I_{\text{RC}}(\mathcal{E}) = \log_2 d - S(\rho_{\mathcal{E}}) = \log_2 d - H(\mathbf{p}). \tag{151}$$

To compute the entanglement flux of the channel (upper bound), recall that we have

$$\Phi(\mathcal{E}) := E_{\text{R}}(\rho_{\mathcal{E}}) \leq S(\rho_{\mathcal{E}}||\tilde{\sigma}_s) \ , \tag{152}$$

for some suitable separable state $\tilde{\sigma}_s$. Let us write its spectral decomposition

$$\tilde{\sigma}_s = \sum_k s_k |\lambda_k\rangle\langle\lambda_k|, \tag{153}$$

where $|\lambda_k\rangle$ (s_k) are the orthogonal eigenstates (eigenvalues) of $\tilde{\sigma}_s$. We may then write

$$S(\rho_{\mathcal{E}}||\tilde{\sigma}_s) = -S(\rho_{\mathcal{E}}) - \mathrm{Tr}\left(\rho_{\mathcal{E}} \log_2 \tilde{\sigma}_s\right) = -H(\mathbf{p}) - \sum_k \langle\lambda_k|\rho_{\mathcal{E}}|\lambda_k\rangle \log_2 s_k \ . \tag{154}$$

The separable state $\tilde{\sigma}_s$ may be constructed by applying the channel $I \otimes \mathcal{E}$ to the input separable state

$$\sigma_s = \frac{1}{d}\sum_{i=0}^{d-1} |ii\rangle \langle ii| \, , \tag{155}$$

so that we have the output

$$\tilde{\sigma}_s = \frac{1}{d}\sum_{i=0}^{d-1} |i\rangle\langle i| \otimes \mathcal{E}(|i\rangle\langle i|). \tag{156}$$

This specific choice will be optimal in some cases and suboptimal in others.

Erasure channel in arbitrary finite dimension

Consider a qudit in arbitrary dimension d with computational basis $\{|i\rangle\}$ (results can be easily specified to the case of a qubit $d = 2$). The erasure channel replaces an incoming qudit state ρ with an orthogonal erasure state $|e\rangle$ with some probability p. In other words, we have the action

$$\mathcal{E}_{\mathrm{erase}}(\rho) = (1-p)\rho + p|e\rangle\langle e| \ . \tag{157}$$

The simplicity of this channel relies in the fact that the input states either are perfectly transmitted or they are lost (while in other quantum channels, the input states are all transmitted into generally-different outputs). This feature allows one to apply simple reasonings such as those in ref. [34] which determined the Q_2 of this channel (more precisely, the Q_2 of the qubit erasure channel, but the extension to arbitrary d is trivial).

It is easy to see that this channel is teleportation-covariant (and therefore Choi-stretchable). In fact, any input unitary U applied to the state ρ is mapped into an output augmented unitary $U \oplus I$, i.e., we may write

$$\mathcal{E}_{\mathrm{erase}}(U\rho U^\dagger) = (U \oplus I)\mathcal{E}_{\mathrm{erase}}(\rho)(U \oplus I)^\dagger. \tag{158}$$

Let us write the Kraus decomposition of this channel

$$\mathcal{E}_{\mathrm{erase}}(\rho) = A\rho A^\dagger + \sum_{i=0}^{d-1} A_i \rho A_i^\dagger, \tag{159}$$

where $A := \sqrt{1-p}I$ (with I being the $d \times d$ identity) and $A_i := \sqrt{p}|e\rangle\langle i|$. We then compute its Choi matrix

$$\rho_{\mathcal{E}_{\mathrm{erase}}} = (1-p)\Phi + \frac{p}{d}(I \otimes |e\rangle\langle e|). \tag{160}$$

Note that $\mathrm{Tr}[\Phi(I \otimes |e\rangle\langle e|)] = 0$, so that Eq. (160) is the spectral decomposition of $\rho_{\mathcal{E}}$ over two orthogonal subspaces, where Φ has eigenvalue $1-p$, and $I \otimes |e\rangle\langle e|$ is degenerate with d eigenvalues equal to p/d. Therefore, it is easy to compute the von Neumann entropy, which is

$$S\left(\rho_{\mathcal{E}_{\mathrm{erase}}}\right) = -(1-p)\log_2(1-p) - p\log_2\left(\frac{p}{d}\right). \tag{161}$$

To compute the entanglement flux of the channel, we consider the separable state $\tilde{\sigma}_s$ in Eq. (156), which here becomes

$$\tilde{\sigma}_s = \frac{1}{d}\sum_{i=0}^{d-1} \left[(1-p)|ii\rangle\langle ii| + p|i,e\rangle\langle i,e|\right]. \tag{162}$$

We have now all the elements to be used in Eq. (154), which provides

$$\Phi(\mathcal{E}_{\mathrm{erase}}) \leq S(\rho_{\mathcal{E}_{\mathrm{erase}}}||\tilde{\sigma}_s) = (1-p)\log_2 d. \tag{163}$$

For the lower bound, one can easily check that the coherent and reverse coherent information of this channel are not sufficient to reach the upper bound, since we get

$$I_{\mathrm{C}}(\mathcal{E}_{\mathrm{erase}}) = (1-2p)\log_2 d, \quad I_{\mathrm{RC}}(\mathcal{E}_{\mathrm{erase}}) = (1-p)\log_2 d - H_2(p), \tag{164}$$

where the extra term $H_2(p)$ is the binary Shannon entropy. Note that these quantities are achievable rates for one-way entanglement distribution but not necessarily the optimal rates. Indeed it is easy to find a strategy based on one-way backward CCs which reaches $(1-p)\log_2 d$. This follows the same reasoning of ref. [34].

Alice can send halves of EPR states to Bob in large n uses of the channel. A fraction $1-p$ will be perfectly distributed. The identification of these good cases can be done by Bob performing a dichotomic POVM $\{|e\rangle\langle e|, I-|e\rangle\langle e|\}$ on each received system and communicating to Alice which instances were perfectly transmitted. At that point Alice and Bob possess $n(1-p)$ EPR states with $\log_2 d$ ebits each. On average this gives a rate of $(1-p)\log_2 d$ ebits per channel use. Thus, one may write

$$D_1(\rho_{\mathcal{E}_{\mathrm{erase}}}) \geq (1-p)\log_2 d\ , \tag{165}$$

whose combination with Eq. (163) provides

$$\mathcal{C}(\mathcal{E}_{\mathrm{erase}}) = D_2(\mathcal{E}_{\mathrm{erase}}) = Q_2(\mathcal{E}_{\mathrm{erase}}) = K(\mathcal{E}_{\mathrm{erase}}) = \Phi(\mathcal{E}_{\mathrm{erase}}) = (1-p)\log_2 d. \tag{166}$$

Since the two-way quantum capacity of the erasure channel is already known [34], our novel result regards the determination of its secret key capacity

$$K(\mathcal{E}_{\mathrm{erase}}) = (1-p)\log_2 d. \tag{167}$$

It is clear that, for qubits, we have $K(\mathcal{E}_{\mathrm{erase}}) = 1-p$.

Qubit Pauli channels

Consider a Pauli channel $\mathcal{P}$ acting on a qubit state ρ. The Kraus representation of this channel is

$$\mathcal{P}(\rho) = \sum_{k=0}^{3} p_k P_k \rho P_k^{\dagger} = p_0\rho + p_1 X\rho X + p_2 Y\rho Y + p_3 Z\rho Z, \tag{168}$$

where $\mathbf{p} := \{p_k\}$ is a probability distribution and $P_k \in \{I, X, Y, Z\}$ are Pauli operators, with I the identity and

$$X := \begin{pmatrix} 0 & 1 \\ 1 & 0 \end{pmatrix},\ Y := \begin{pmatrix} 0 & -i \\ i & 0 \end{pmatrix},\ Z := \begin{pmatrix} 1 & 0 \\ 0 & -1 \end{pmatrix}. \tag{169}$$

It is easy to check that a Pauli channel is teleportation-covariant and, therefore, Choi-stretchable. Teleportation covariance simply comes from the fact that the Pauli operators (qubit teleportation unitaries) either commute or anticommute with the other Pauli operators (Kraus operators of the channel). For a Pauli channel we can also write the stronger condition

$$[\rho_{\mathcal{P}}, P_k^{*} \otimes P_k] = 0 \ \text{ for any } k, \tag{170}$$

i.e., its Choi matrix is invariant under twirling operations restricted to the generators of the Pauli group. In fact, the Choi matrix of a Pauli channel is Bell-diagonal, i.e., it has spectral decomposition

$$\rho_{\mathcal{P}} = \sum_{k=0}^{3} p_k \Phi_k, \tag{171}$$

where the eigenvalues p_k are the channel probabilities, and the eigenvectors Φ_k are the four Bell states

$$\left\{ \frac{|00\rangle \pm |11\rangle}{\sqrt{2}},\ \frac{|10\rangle \pm |01\rangle}{\sqrt{2}} \right\}. \tag{172}$$

It is clear that $S(\rho_{\mathcal{P}}) = H(\mathbf{p})$. Then, using the separable state $\tilde{\sigma}_s$ as in Eq. (156), we derive the following upper bound for the entanglement flux of this channel

$$\Phi(\mathcal{P}) \leq 1 - H(\mathbf{p}) + H_2(p_1+p_2). \tag{173}$$

Since a Pauli channel is unital, its (reverse) coherent information is just given by $I_{(\mathrm{R})\mathrm{C}}(\mathcal{P}) = 1 - H(\mathbf{p})$. Therefore, the two-way capacity of a Pauli channel with arbitrary distribution $\mathbf{p} := \{p_k\}$ must satisfy

$$1 - H(\mathbf{p}) \leq \mathcal{C}(\mathcal{P}) \leq 1 - H(\mathbf{p}) + H_2(p_1 + p_2). \tag{174}$$

Latter result can be made stronger by exploiting the fact that $\rho_{\mathcal{P}}$ is Bell-diagonal. For any such a state we can compute the REE by using the formula of ref. [35]. In fact, let us set $p_{\max} := \max\{p_k\}$, then we may write

$$E_{\mathrm{R}}(\rho_{\mathcal{P}}) = \begin{cases} 1 - H_2(p_{\max}) & \text{if } p_{\max} \geq \frac{1}{2} \\ 0 & \text{otherwise.} \end{cases} \tag{175}$$

Thus, we have the tighter upper bound

$$1 - H(\mathbf{p}) \leq \mathcal{C}(\mathcal{P}) \leq \Phi(\mathcal{P}) = \begin{cases} 1 - H_2(p_{\max}) & \text{if } p_{\max} \geq \frac{1}{2} \\ 0 & \text{otherwise.} \end{cases} . \tag{176}$$

In the following subsections, we specialize this result to depolarising and dephasing channels.

Qubit depolarising channel

This is a Pauli channel with probability distribution

$$\mathbf{p} = \left\{1 - \frac{3p}{4}, \frac{p}{4}, \frac{p}{4}, \frac{p}{4}\right\}, \tag{177}$$

so that we have

$$\mathcal{P}_{\mathrm{depol}}(\rho) = \left(1 - \frac{3p}{4}\right)\rho + \frac{p}{4}(X\rho X + Y\rho Y + Z\rho Z) = (1-p)\rho + p\frac{I}{2} . \tag{178}$$

Let us set

$$\kappa(p) := 1 - H_2\left(\frac{3p}{4}\right). \tag{179}$$

Then, from Eq. (176), we derive the following bounds for the two-way capacity of the depolarising channel

$$\kappa(p) - \frac{3p}{4}\log_2 3 \leq \mathcal{C}(\mathcal{P}_{\mathrm{depol}}) \leq \kappa(p), \tag{180}$$

for $p \leq 2/3$, while $\mathcal{C}(\mathcal{P}_{\mathrm{depol}}) = 0$ otherwise.

Qubit dephasing channel

This is a Pauli channel with probability distribution $\mathbf{p} = \{1-p, 0, 0, p\}$, so that we have

$$\mathcal{P}_{\mathrm{deph}}(\rho) = (1-p)\rho + pZ\rho Z. \tag{181}$$

It is easy to see that $H(\mathbf{p}) = H_2(p_{\max}) = H_2(p)$, so that Eq. (176) leads to

$$\mathcal{C}(\mathcal{P}_{\mathrm{deph}}) = D_2(\mathcal{P}_{\mathrm{deph}}) = Q_2(\mathcal{P}_{\mathrm{deph}}) = K(\mathcal{P}_{\mathrm{deph}}) = \Phi(\mathcal{P}_{\mathrm{deph}}) = 1 - H_2(p), \tag{182}$$

which also coincides with the unassisted quantum capacity of this channel $Q(\mathcal{P}_{\mathrm{deph}})$ [36].

Pauli channels in arbitrary finite dimension

Let us now consider Pauli channels $\mathcal{P}_d$ in arbitrary dimension $d \geq 2$. These qudit channels are also called "Weyl channels" and they have Kraus representation

$$\mathcal{P}_d(\rho) = \sum_{a,b=0}^{d-1} p_{ab}(X^a Z^b)\rho(X^a Z^b)^\dagger, \tag{183}$$

where p_{ab} is a probability distribution for $a, b \in \mathbb{Z}_d := \{0, 1, \ldots, d-1\}$. Here X and Z are generalized Pauli operators whose action on the computational basis $\{|j\rangle\}$ is given by

$$X|j\rangle = |j \oplus 1\rangle \ , \ Z|j\rangle = \omega^j |j\rangle \ , \tag{184}$$

where $\oplus$ is the modulo d addition and

$$\omega := \exp(i2\pi/d). \tag{185}$$

These operators satisfy the generalized commutation relation

$$Z^b X^a = \omega^{ab} X^a Z^b. \tag{186}$$

Not only for $d = 2$ (qubits) but also at any $d \geq 2$ a Pauli channel is teleportation-covariant.

The channel's Choi matrix $\rho_{\mathcal{P}_d}$ is Bell-diagonal with eigenvalues $\{p_{ab}\}$, so that we may write its von Neumann entropy in terms of the Shannon entropy as follows

$$S(\rho_{\mathcal{P}_d}) = H(\{p_{ab}\}). \tag{187}$$

Note that the Choi matrix can also be written as

$$\rho_{\mathcal{P}_d} = \frac{1}{d}\sum_{a,b,j,k}^{d-1} p_{ab}(I \otimes X^a Z^b)|jj\rangle\langle kk|(I \otimes X^a Z^b)^\dagger = \frac{1}{d}\sum_{a,b,j,k}^{d-1} p_{ab}\ \omega^{b(j-k)}|j, j \oplus a\rangle\langle k, k \oplus a|. \tag{188}$$

Then, let us consider a separable state $\tilde{\sigma}_s$ which is constructed as in Eq. (156). This state can be re-written as

$$\tilde{\sigma}_s = \frac{1}{d}\sum_{a,b,i=0}^{d-1} p_{ab}|i, i \oplus a\rangle\langle i, i \oplus a|. \tag{189}$$

By applying Eq. (154), we find

$$\Phi(\mathcal{P}_d) \leq \log_2 d - H(\{p_{ab}\}) + H(\{p_a\}), \tag{190}$$

where $p_a := \sum_{b=0}^{d-1} p_{ab}$. Since the d-dimensional Pauli channel is unital, we may also write $I_{(\mathrm{R})\mathrm{C}}(\mathcal{P}_d) = \log_2 d - H(\{p_{ab}\})$, so that we derive the following bounds for its two-way capacity

$$\log_2 d - H(\{p_{ab}\}) \leq \mathcal{C}(\mathcal{P}_d) \leq \log_2 d - H(\{p_{ab}\}) + H(\{p_a\}), \tag{191}$$

which generalizes Eq. (174) to arbitrary dimension d. In the following two subsections, we consider the specific cases of the depolarising and dephasing channels in arbitrary finite dimension d.

Depolarising channel in arbitrary finite dimension

Consider a depolarising channel acting on a qudit with dimension $d \geq 2$. This channel can be written as

$$\mathcal{P}_{d\text{-depol}}(\rho) = (1-p)\rho + p\frac{I}{d} = A\rho A^\dagger + \sum_{i,j=0}^{d-1} A_{ij}\rho A_{ij}^\dagger, \tag{192}$$

where $A = \sqrt{1-p}I$ and $A_{ij} = \sqrt{p/d}|i\rangle\langle j|$. Its Choi matrix is the isotropic state

$$\rho_{\mathcal{P}_{d\text{-depol}}} = (1-p)|\Phi\rangle\langle\Phi| + \frac{p}{d^2} I \otimes I, \tag{193}$$

satisfying the twirling condition

$$\left[\rho_{\mathcal{P}_{d\text{-depol}}}, U^* \otimes U\right] = 0, \tag{194}$$

for any qudit unitary U.

The REE of an isotropic state can be evaluated exactly by using the formula of ref. [37]. Thus we can exactly compute the entanglement flux of the d-dimensional depolarising channel. Let us set

$$f := \frac{d^2-1}{d^2}p, \quad \kappa(d,p) := \log_2 d - H_2(f) - f\log_2(d-1). \tag{195}$$

Then, we may write the following expression

$$\Phi(\mathcal{P}_{d\text{-depol}}) = E_{\mathrm{R}}\left(\rho_{\mathcal{P}_{d\text{-depol}}}\right) = \begin{cases} \kappa(d,p) & \text{if } p \leq \frac{d}{d+1}, \\ 0 & \text{otherwise.} \end{cases} \tag{196}$$

Because the depolarising channel is unital, we may use Eq. (151) to compute its (reverse) coherent information. We specifically find

$$I_{(\mathrm{R})\mathrm{C}}(\mathcal{P}_{d\text{-depol}}) = \log_2 d - H_2(f) - f\log_2(d^2-1) = \kappa(d,p) - f\log_2(d+1). \tag{197}$$

Thus, the two-way capacity of this channel must satisfy the bounds

$$\kappa(d,p) - f\log_2(d+1) \leq \mathcal{C}(\mathcal{P}_{d\text{-depol}}) \leq \kappa(d,p), \tag{198}$$

for $p \leq d/(d+1)$, while zero otherwise.

Dephasing channel in arbitrary finite dimension

Consider a generalized dephasing channel affecting a qudit in arbitrary dimension $d \geq 2$. This channel has Kraus representation [38, 39]

$$\mathcal{P}_{d\text{-deph}}(\rho) = \sum_{i=0}^{d-1} P_i Z^i \rho (Z^\dagger)^i, \quad , \tag{199}$$

where Z is the generalized Pauli (phase-flip) operator defined in Eq. (184), and P_i is the probability of i phase flips.

The channel's Choi matrix is

$$\rho_{\mathcal{P}_{d\text{-deph}}} = \sum_{mjl} \frac{P_m}{d} \exp\left[\frac{2i\pi}{d}(j-l)m\right] |jj\rangle\langle ll|. \tag{200}$$

By diagonalizing, we find d non-zero eigenvalues $\mathbf{P} := \{P_0, \ldots, P_{d-1}\}$, so that the Von Neumann entropy is given by

$$S(\rho_{\mathcal{P}_{d\text{-deph}}}) = H(\mathbf{P}). \tag{201}$$

The separable state $\tilde{\sigma}_s$ in Eq. (156) turns out to be diagonal in the computational basis and takes the form

$$\tilde{\sigma}_s = \sum_{i=0}^{d-1} \frac{1}{d} |ii\rangle\langle ii| \ . \tag{202}$$

Thus, using Eq. (154), we find

$$\Phi(\mathcal{P}_{d\text{-deph}}) \leq S(\rho_{\mathcal{P}_{d\text{-deph}}} || \tilde{\sigma}_s) = \log_2 d - H(\mathbf{P}). \tag{203}$$

Since this channel is unital, from Eq. (151) we have that its (reverse) coherent information is $I_{(\mathrm{R})\mathrm{C}}(\mathcal{P}_{d\text{-deph}}) = \log_2 d - H(\mathbf{P})$, so that lower and upper bounds coincide. This means that this channel is distillable and its two-way capacity is equal to

$$C(\mathcal{P}_{d\text{-deph}}) = D_2(\mathcal{P}_{d\text{-deph}}) = Q_2(\mathcal{P}_{d\text{-deph}}) = K(\mathcal{P}_{d\text{-deph}}) = \Phi(\mathcal{P}_{d\text{-deph}}) = \log_2 d - H(\mathbf{P}). \tag{204}$$

Amplitude damping channel

The amplitude damping channel describes the process of energy dissipation through spontaneous emission in a two-level system. Its application to an input qubit state is defined by the Kraus representation

$$\mathcal{E}_{\mathrm{damp}}(\rho) = \textstyle\sum_{i=0,1} A_i \rho A_i^{\dagger}, \tag{205}$$

where

$$A_0 := |0\rangle\langle 0| + \sqrt{1-p}\,|1\rangle\langle 1|\,, \quad A_1 := \sqrt{p}\,|0\rangle\langle 1|\,, \tag{206}$$

and p is the probability of damping. This channel is not teleportation-covariant. In fact, because we have

$$|0\rangle\langle 0| \to |0\rangle\langle 0|\,, \quad |1\rangle\langle 1| \to p\,|0\rangle\langle 0| + (1-p)\,|1\rangle\langle 1|\,, \tag{207}$$

there is no unitary U able to realize $U\mathcal{E}_{\mathrm{damp}}(|0\rangle\langle 0|)U^{\dagger} = \mathcal{E}_{\mathrm{damp}}(X\,|0\rangle\langle 0|\,X)$ for Pauli operator X.

The amplitude damping channel can be decomposed as

$$\mathcal{E}_{\mathrm{damp}} = \mathcal{E}_{\mathrm{CV}\to\mathrm{DV}} \circ \mathcal{E}_{\eta(p)} \circ \mathcal{E}_{\mathrm{DV}\to\mathrm{CV}}, \tag{208}$$

where $\mathcal{E}_{\mathrm{DV}\to\mathrm{CV}}$ is an identity mapping from the original qubit (e.g. a spin) to a single-rail qubit, which is the subspace of a bosonic mode spanned by the vacuum and the single photon states; then, $\mathcal{E}_{\eta(p)}$ is a lossy channel with transmissivity $\eta(p) := 1-p$; finally, $\mathcal{E}_{\mathrm{CV}\to\mathrm{DV}}$ is an identity mapping from the single-rail qubit to the original qubit. Note that the two mappings can be performed via perfect hybrid teleportation and the middle lossy channel preserves the 2-dimensional effective Hilbert space of the system.

Thanks to this decomposition, we can include $\mathcal{E}_{\mathrm{DV}\to\mathrm{CV}}$ in Alice's LOs and $\mathcal{E}_{\mathrm{CV}\to\mathrm{DV}}$ into Bob's LOs. The middle lossy channel $\mathcal{E}_{\eta(p)}$ can therefore be stretched into its asymptotic Choi matrix $\rho_{\mathcal{E}_{\eta(p)}}$. Overall, this means that the amplitude damping channel can be stretched into the asymptotic resource state $\sigma = \rho_{\mathcal{E}_{\eta(p)}}$ by means of an asymptotic simulation. By applying teleportation stretching, we therefore reduce the output of an adaptive protocol to the form

$$\rho^n_{\mathbf{ab}} := \rho_{\mathbf{ab}}(\mathcal{E}^{\otimes n}_{\mathrm{damp}}) = \bar{\Lambda}\left(\rho^{\otimes n}_{\mathcal{E}_{\eta(p)}}\right), \tag{209}$$

where both $\bar{\Lambda}$ and $\rho_{\mathcal{E}_{\eta(p)}}$ are intended as asymptotic limits. Thus, our reduction method provides the upper bound

$$\mathcal{C}(\mathcal{E}_{\mathrm{damp}}) \leq \Phi\left[\mathcal{E}_{\eta(p)}\right] = -\log_2 p. \tag{210}$$

We can combine the latter result with the fact that we cannot exceed the logarithm of the dimension of the input Hilbert space (see this simple "dimensionality bound" in the main text, in the discussion just before Proposition 5). This leads to

$$\mathcal{C}(\mathcal{E}_{\mathrm{damp}}) \leq \min\{1, -\log_2 p\}. \tag{211}$$

The best lower bound is given by optimizing the reverse coherent information over the input states $\rho_u = \mathrm{diag}(1-u, u)$ for $0 \leq u \leq 1$. In fact, we have [6]

$$I_{\mathrm{RC}}(p) := \max_u I_{\mathrm{RC}}(\mathcal{E}_{\mathrm{damp}}, \rho_u) = \max_u \{H_2(u) - H_2(up)\}. \tag{212}$$

This is an achievable lower bound for entanglement distribution assisted by a final round of backward CCs. Note that this is strictly higher than the $Q_1 = Q$ of the channel, which is given by [6]

$$Q_1(\mathcal{E}_{\mathrm{damp}}) = \max_u \{H_2[u(1-p)] - H_2(up)\}. \tag{213}$$

Thus, in total, we may write

$$I_{\mathrm{RC}}(p) \leq \mathcal{C}(\mathcal{E}_{\mathrm{damp}}) \leq \min\{1, -\log_2 p\}, \tag{214}$$

which is shown in Supplementary Fig. 1a. See the next section for the derivation of a tighter upper bound which is based on the squashed entanglement.

Amplitude damping channel: Upper bound based on the squashed entanglement

An alternative upper bound for the two-way capacity of a quantum channel is its squashed entanglement, i.e., we may write [40]

$$\mathcal{C}(\mathcal{E}) \leq E_{\rm sq}(\mathcal{E}). \tag{215}$$

The squashed entanglement of an arbitrary channel $\mathcal{E}$, from system A to system B, is defined as [40]

$$E_{\rm sq}(\mathcal{E}) := \frac{1}{2} \max_{\rho_A} \inf_{V_{C\to EF}} [S(B|E)_\omega + S(B|F)_\omega], \tag{216}$$

where ρ_A is an arbitrary input state, and ω is the global output state

$$\omega_{BEF} := V_{C\to EF}[U^{\mathcal{E}}_{A\to BC}(\rho_A)], \tag{217}$$

with $U^{\mathcal{E}}_{A\to BC}$ being an isometric extension of $\mathcal{E}$ and $V_{C\to EF}$ being an arbitrary "squashing isometry".

In Eq. (216), the terms in the brackets are conditional von Neumann entropies computed over ω_{BEF}, i.e.,

$$S(B|E)_\omega = S(BE)_\omega - S(E)_\omega, \quad S(B|F)_\omega = S(BF)_\omega - S(F)_\omega. \tag{218}$$

Then note that the most general input state reads

$$\rho_A = \begin{pmatrix} 1-\gamma & c^* \\ c & \gamma \end{pmatrix}, \tag{219}$$

where $\gamma \in [0,1]$ is the population of the excited state $|1\rangle$, while the off-diagonal term $|c| \leq \sqrt{(1-\gamma)\gamma}$ accounts for coherence. Thus, the maximization in Eq. (216) is mapped into a maximization over parameters γ and c.

Let us compute the squashed entanglement of the amplitude damping channel $\mathcal{E}_{\rm damp}$. Recall that its action is described by Eq. (205) with Kraus operators as in Eq. (206). In the computational basis $\{|00\rangle, |01\rangle, |10\rangle, |11\rangle\}$, the unitary dilation of $\mathcal{E}_{\rm damp}$ is therefore given by the following matrix

$$U_p = \begin{pmatrix} 1 & 0 & 0 & 0 \\ 0 & \sqrt{1-p} & \sqrt{p} & \\ 0 & -\sqrt{p} & \sqrt{1-p} & 0 \\ 0 & 0 & 0 & 1 \end{pmatrix}, \tag{220}$$

so that we may write

$$\mathcal{E}_{\rm damp}(\rho_A) = {\rm Tr}_C[U_p(\rho_A \otimes |0\rangle\langle 0|_C)U_p^\dagger], \tag{221}$$

where C is an environmental qubit prepared in the fundamental state $|0\rangle$. It is clear that Eq. (221) expresses the isometric extension of the channel, i.e., it corresponds to $\mathcal{E}_{\rm damp}(\rho_A) = {\rm Tr}_C[U^{\rm damp}_{A\to BC}(\rho_A)]$.

As a squashing channel we consider another amplitude damping channel but with damping probability equal to 1/2, so that its unitary dilation is $V = U_{1/2}$. In other words, we consider the squashing isometry $V_{C\to EF} = [U^{\rm damp}_{C\to EF}]_{p=1/2}$ (so that we are more precisely deriving an upper bound of the squashed entanglement of the channel). Let us derive the global output state ω_{BEF} step-by-step.

The state of systems B and C at the output of the dilation U_p is given by

$$\rho_{BC} := U_p(\rho_A \otimes |0\rangle\langle 0|_C)U_p^\dagger = \begin{pmatrix} 1-\gamma & \sqrt{p}c^* & \sqrt{1-p}c^* & 0 \\ c\sqrt{p} & p\gamma & \sqrt{1-p}\sqrt{p}\gamma & 0 \\ c\sqrt{1-p} & \sqrt{1-p}\sqrt{p}\gamma & (1-p)\gamma & 0 \\ 0 & 0 & 0 & 0 \end{pmatrix}. \tag{222}$$

Now the system C is sent through the squashing amplitude damping channel with probability 1/2. At the output of the dilation $U_{1/2}$ we have the final output state

$$\omega_{BEF} = (I_B \otimes U_{1/2})\rho_{BC} \otimes |0\rangle\langle 0|_F (I_B \otimes U_{1/2})^\dagger = \begin{pmatrix} 1-\gamma & \frac{\sqrt{p}c^*}{\sqrt{2}} & \frac{\sqrt{p}c^*}{\sqrt{2}} & 0 & \sqrt{1-p}c^* & 0 & 0 & 0 \\ \frac{c\sqrt{p}}{\sqrt{2}} & \frac{p\gamma}{2} & \frac{p\gamma}{2} & 0 & \frac{\sqrt{(1-p)p\gamma}}{\sqrt{2}} & 0 & 0 & 0 \\ \frac{c\sqrt{p}}{\sqrt{2}} & \frac{p\gamma}{2} & \frac{p\gamma}{2} & 0 & \frac{\sqrt{(1-p)p\gamma}}{\sqrt{2}} & 0 & 0 & 0 \\ 0 & 0 & 0 & 0 & 0 & 0 & 0 & 0 \\ c\sqrt{1-p} & \frac{\sqrt{(1-p)p\gamma}}{\sqrt{2}} & \frac{\sqrt{(1-p)p\gamma}}{\sqrt{2}} & 0 & \gamma - p\gamma & 0 & 0 & 0 \\ 0 & 0 & 0 & 0 & 0 & 0 & 0 & 0 \\ 0 & 0 & 0 & 0 & 0 & 0 & 0 & 0 \\ 0 & 0 & 0 & 0 & 0 & 0 & 0 & 0 \end{pmatrix}. \tag{223}$$

We now proceed with the calculation of the entropies in Eq. (218), which are obtained from the eigenvalues of the reduced states ρ_{BE}, ρ_{BF}, ρ_E and ρ_F. We obtain

$$\rho_E = \rho_F = \begin{pmatrix} 1 - \frac{p\gamma}{2} & \frac{\sqrt{p}c^*}{\sqrt{2}} \\ \frac{c\sqrt{p}}{\sqrt{2}} & \frac{p\gamma}{2} \end{pmatrix}, \tag{224}$$

with eigenvalues

$$\lambda_{1,2} = \frac{1}{2}\left(1 \pm \sqrt{2|c|^2 p + (p\gamma - 1)^2}\right). \tag{225}$$

The eigenvalues of ρ_{BE} and ρ_{BF} are too complicated to be reported here but it is easy to check that, exactly as for $\lambda_{1,2}$ in previous Eq. (225), their dependence on c is just through the modulus $|c|$, so that we can choose c to be real without losing generality.

Because c is real, we also have that the entropic functional $F(\rho) = S(B|E)_\omega + S(B|F)_\omega$ computed over the input state ρ is exactly the same as that computed over the state $Z\rho Z$, with Z being the phase-flip Pauli operator. Using the latter observation, together with the concavity of the conditional quantum entropy, one simply has

$$F(\rho) = \frac{F(\rho) + F(Z\rho Z)}{2} \leq F\left(\frac{\rho + Z\rho Z}{2}\right) = F(\bar{\rho}), \tag{226}$$

where $\bar{\rho}$ is diagonal. This means that we may reduce the maximization to diagonal input states ($c = 0$).

As a result, we may just consider

$$\rho_E = \rho_F = \begin{pmatrix} 1 - \frac{p\gamma}{2} & 0 \\ 0 & \frac{p\gamma}{2} \end{pmatrix}, \tag{227}$$

with eigenvalues

$$\lambda_1 = \frac{p\gamma}{2},\ \lambda_2 = 1 - \frac{p\gamma}{2}, \tag{228}$$

and

$$\rho_{BE} = \rho_{BF} = \begin{pmatrix} \frac{1}{2}(p-2)\gamma + 1 & 0 & 0 & 0 \\ 0 & \frac{p\gamma}{2} & \frac{\sqrt{(1-p)p\gamma}}{\sqrt{2}} & 0 \\ 0 & \frac{\sqrt{(1-p)p\gamma}}{\sqrt{2}} & \gamma - p\gamma & 0 \\ 0 & 0 & 0 & 0 \end{pmatrix}, \tag{229}$$

with eigenvalues

$$\nu_1 = \frac{\gamma}{2}(2-p),\ \nu_2 = 1 - \nu_1,\ \nu_3 = \nu_4 = 0. \tag{230}$$

From the previous eigenvalues, we compute the conditional quantum entropies in Eq. (218). Thus, we find that the squashed entanglement of the amplitude damping channel must satisfy the bound

$$E_{\mathrm{sq}}(\mathcal{E}_{\mathrm{damp}}) \leq \max_\gamma \{H_2(\nu_1) - H_2(\lambda_1)\}, \tag{231}$$

where H_2 is the binary Shannon entropy of Eq. (37). In particular, the function $H_2(\nu_1) - H_2(\lambda_1)$ is concave and symmetric in γ, so that the maximum is reached for $\gamma = 1/2$, which corresponds to a maximally mixed state at the input. This reduces Eq. (231) to the simple bound

$$E_{\mathrm{sq}}(\mathcal{E}_{\mathrm{damp}}) \leq H_2\left(\frac{1}{2} - \frac{p}{4}\right) - H_2\left(1 - \frac{p}{4}\right). \tag{232}$$

If we choose a squashing amplitude damping channel with generic probability of damping η and we repeat the calculation from the beginning we obtain the following bound for the squashed entanglement

$$E_{\mathrm{sq}}(\mathcal{E}_{\mathrm{damp}}) \leq \frac{1}{2}\max_\gamma \min_\eta \{H_2(\gamma - p\gamma\eta) + H_2[\gamma(1-p+p\eta)] - H_2[p\gamma(1-\eta)] - H_2(p\gamma\eta)\}. \tag{233}$$

The minimum of the function inside the curly bracket is for $\eta = 1/2$, so our choice of a balanced amplitude damping channel as a squashing channel is now justified. Note that the sub-optimal choice $\eta = 0$ corresponds to use the identity as squashing channel; correspondingly, the right hand side of Eq. (233) becomes half of the entanglement-assisted classical capacity C_{A} of the amplitude damping channel, i.e.,

$$E_{\text{sq}}(\mathcal{E}_{\text{damp}}) \leq \frac{1}{2} C_{\text{A}}(\mathcal{E}_{\text{damp}}) = \frac{1}{2} \max_{\gamma} \{H_2(\gamma) + H_2[\gamma(1-p)] - H_2(p\gamma)\} . \quad (234)$$

In conclusion, combining the lower bound of Eq. (212) and the upper bound of Eq. (232), we find that the two-way capacity of the amplitude damping channel is within the sandwich

$$\max_{u} \{H_2(u) - H_2(up)\} \leq \mathcal{C}(\mathcal{E}_{\text{damp}}) \leq H_2\left(\frac{1}{2} - \frac{p}{4}\right) - H_2\left(1 - \frac{p}{4}\right) . \quad (235)$$

This is shown in Supplementary Fig. 1b, which also contains a comparison with the previous upper bound based on the REE. Note that, for high damping ($p \simeq 1$), the upper bound in Eq. (235) provides the scaling of $\lesssim 0.793(1-p)$ bits per channel use, while Eq. (211) provides the scaling of $\lesssim 1.44(1-p)$ bits per channel use.

Supplementary Note 6. MAXIMUM RATES ACHIEVABLE BY CURRENT QKD PROTOCOLS

We consider the state of the art in high-rate QKD, by analyzing the maximum rates which are achievable by current practical protocols in CVs and DVs. We assume the optimal asymptotic case of infinitely long keys, so that finite-size effects are negligible. We also assume ideal parameters. For CVs this means: Unit detector efficiency, zero excess noise, large modulation and unit reconciliation efficiency. For DVs this means: Unit detector efficiencies, zero dark count rates, zero intrinsic error, unit error correction efficiency, and no other internal loss in the devices. Note that all the following results are already present in the literature or are easily derivable from those in the literature. They are given to the reader for the sake of completeness.

Continuous-variable protocols

• No-switching protocol [41]. This is the practical CV protocol with the highest secret key rate. It is based on coherent states and heterodyne detection. In reverse reconciliation (RR), its maximum secret key rate over a lossy channel with transmissivity η is equal to

$$R_{\text{no-switch}} = \log_2 \left[\frac{\eta}{e(1-\eta)}\right] + s\left(\frac{2-\eta}{2\eta}\right) , \quad (236)$$

where $s(\cdot)$ is the entropic function given in Eq. (95). For high loss ($\eta \simeq 0$), it scales as $\simeq \eta/2\ln 2$, which is 1/2 of the secret key capacity.

• Switching protocol [42, 43]. This was the first practical CV protocol. It is based on coherent states and homodyne detection (with switching between the two quadratures). In RR, it reaches the rate

$$R_{\text{switch}} = \frac{1}{2} \log_2 \left(\frac{1}{1-\eta}\right) , \quad (237)$$

which is 1/2 of the secret key capacity. For high loss, it clearly scales as the previous protocol.

• CV measurement-device-independent (MDI) protocol [44, 45]. This is based on coherent states sent to an untrusted relay implementing a CV Bell detection. Alice-relay channel has transmissivity η_A and Bob-relay channel has transmissivity η_B, so that the total Alice-Bob channel transmissivity is $\eta = \eta_A \eta_B$. In the symmetric configuration with the relay perfectly in the middle ($\eta_A = \eta_B$) [44, 46], it has maximum rate

$$R_{\text{CVMDI-sym}} = \log_2 \left[\frac{\eta}{e^2(1-\sqrt{\eta})}\right] + s\left(\frac{1}{\sqrt{\eta}} - \frac{1}{2}\right) . \quad (238)$$

In the asymmetric configuration ($\eta_A \neq \eta_B$), it has maximum rate

$$R_{\text{CVMDI-asym}} = s\left(\frac{1}{\eta_B} - \frac{1}{2}\right) - s\left(\frac{2-\eta_A-\eta_B}{2|\eta_A-\eta_B|}\right) + \log_2\left(\frac{\eta_A\eta_B}{e|\eta_A-\eta_B|}\right) . \quad (239)$$

In particular, in the most asymmetric configuration, where the relay coincides with Alice ($\eta_A = 1$) [44, 47], we recover the one-way rate of Eq. (236).

• CV two-way protocols [48]. In the first main variant, Bob sends coherent states to Alice, who randomly displaces their amplitudes before sending them back to Bob for heterodyne detection. In RR (Bob as encoder), this protocol has maximum rate

$$R_{\text{2way-het}} = \frac{1}{2}\left\{ s\left[\frac{2-\eta+\eta^2}{2\eta(1+\eta)}\right] + \log_2\left[\frac{\eta(1+\eta)}{e(1-\eta)}\right]\right\}. \tag{240}$$

In the second main variant, the protocol runs as before except that Bob's measurement is homodyne detection (with switching between the quadratures). In RR, it has maximum rate [49]

$$R_{\text{2way-hom}} = \frac{1}{4}\log_2\left(\frac{1+\eta^2}{1-\eta}\right). \tag{241}$$

It is easy to check that both the variants scale as $\simeq \eta/4\ln 2$ for high loss. Despite the fact that two-way protocols have lower key rates than one-way protocols in a lossy channel, they are more robust when excess noise is present. In this case, one considers the "security threshold" of the protocol which is defined as the maximum tolerable excess noise above which the rate becomes negative. Two-way protocols have higher security thresholds than one-way protocols [48, 49].

Discrete-variable protocols

Here we consider various DV protocols. As said before, we assume the optimal asymptotic case of infinitely long keys and also ideal parameters, which here means: Unit detector efficiencies, zero dark count rates, zero intrinsic error, unit error correction efficiency, and no other internal loss in the devices. Under these assumptions, we consider the ideal BB84 protocol with single photon sources [50], the BB84 with weak coherent pulses and decoy states [51, 52], and DV-MDI-QKD [53, 54].

Let us consider the BB84 protocol [50] assuming that Alice's source generates perfect single-photon pulses. The general formula of the key rate can be found in ref. [51]. It reduces to the following expression

$$R = \bar{R}\left\{[1 - H_2(Q)] - \delta(Q)\right\}, \tag{242}$$

where H_2 is the binary Shannon entropy. In Eq. (242), $\delta(Q) = f\ H_2(Q)$ is a function accounting for the leak of information from imperfect error correction, $f \geq 1$ is the efficiency of the classical error correction codes, Q is the total error rate (QBER), and $\bar{R}$ is the total detection rate after quantum communication (the raw key). Under ideal conditions of zero dark-count rates, unit efficiency detectors, perfect visibility, and perfect classical error correction ($f = 1$), one has $Q = 0$ and obtains the following maximum rate $R_{\text{BB84-1ph}} = \eta/2$, setting the maximum rate for the current DV protocols.

A realistic photon source is a device emitting attenuated coherent pulses. In this case, the performance of the protocol depends on an additional parameter which is the intensity of the source. In the BB84 protocol, with weak coherent pulses and decoy states [52], Alice randomly changes the intensity μ of the pulses, and reveals publicly their values during the final classical communication. In this way Eve cannot adapt her attacks during the quantum communication. The μ-dependent key rate of the protocol is given by [51]

$$R^\mu = \bar{R}\left\{Y_0^\mu + Y_1^\mu\left[1 - H_2\left(\frac{Q^\mu}{Y_1^\mu}\right)\right] - \delta(Q^\mu)\right\}, \tag{243}$$

where Q^μ is the μ-dependent QBER, and $Y_n^\mu = R_n^\mu/\bar{R}$ is the ratio between the μ-dependent detection rate R_n^μ, associated to Alice sending n photons, and the total detection rate $\bar{R}$. Assuming ideal conditions, one finds $R^\mu = e^{-\mu}\eta\mu/2$. The optimal key rate is obtained by maximizing over the intensities, i.e., $R = \max_\mu R^\mu$. It is easy to check that the optimum is given by $\mu = 1$ and the maximum key rate becomes $R_{\text{BB84-decoy}} = \eta/(2e)$.

Finally consider DV-MDI-QKD. The general expression of the key rate is given by the following expression [54]

$$R = P_Z^{11} Y_Z^{11}\left[1 - H_2(e_Z^{11})\right] - G_Z\ \delta(Q_Z), \tag{244}$$

where $P_Z^{11} = \mu_A\mu_B\exp[-(\mu_A+\mu_B)]$ is the joint probability that both emitters (with intensities μ_A and μ_B) generate a single-photon pulse. The quantity Y_Z^{11} gives the gain in the Z-basis (one assumes $Y_X^{11} = Y_Z^{11}$ for the X-basis),

and e_Z^{11} is the error rate in the Z-basis. Finally, the quantity G_Z describes the gain and Q_Z the QBER, both in the Z-basis. Under ideal conditions, the μ-dependent key rate becomes

$$R^{\mu_A \mu_B} = \frac{1}{2} e^{-(\mu_A+\mu_B)} \eta_A \eta_B \mu_A \mu_B, \tag{245}$$

where η_A and η_B are the transmissivities of Alice's and Bob's channels. It is easy to check that the maximum is taken for $\mu_A = \mu_B = 1$, providing $R_{\text{DV-MDI}} = \eta/(2e^2)$.

Supplementary Note 7. ENERGY CONSTRAINTS AND COST OF CLASSICAL COMMUNICATION

Input energy constraints

It is important to remark that the two-way capacities that we computed for Gaussian channels are bounded quantities, which do not diverge even if the maximum is achieved in the limit of infinite input energy (excluding the case of a pathological canonical form). In fact, one may consider an alphabet of input states whose mean number of photons is capped at some finite value $\bar{N}$. This assumption automatically defines a hard-constrained two-way capacity $\mathcal{C}(\mathcal{E}, \bar{N})$. For a bosonic Gaussian channel, $\mathcal{C}(\mathcal{E}, \bar{N})$ is increasing in $\bar{N}$ but also upper-bounded by the entanglement flux of the channel $\Phi(\mathcal{E})$. (In fact, note that all the procedure of teleportation stretching still applies if we enforce an input energy constraint for the adaptive protocols. For instance the constraint can be realized by a pinching map which is then absorbed in Alice's LOs). As a result, the asymptotic limit of the unconstrained capacity $\mathcal{C}(\mathcal{E}) := \lim_{\bar{N}} \mathcal{C}(\mathcal{E}, \bar{N})$ is finite. This is clearly true for $Q_2(\mathcal{E})$, $D_2(\mathcal{E})$ and $K(\mathcal{E})$, but the situation would be different for the two-way classical capacity of the channel.

Another possibility is imposing a "soft constraint" on the input energy. This means to fix the average number of photons at the input to some finite value $\bar{m}$. In this case, it is interesting to see that our "unconstrained" upper bounds remain sufficiently tight even in the presence of such an energy constraint. The best way to show this is considering our main result for the lossy channel with arbitrary transmissivity η, for which we have proven that

$$Q_2(\eta) = D_2(\eta) = K(\eta) = \Phi(\eta) = -\log_2(1-\eta). \tag{246}$$

Even if we constrain the input to $\bar{m}$ mean photons, it is easy to show that:

(1) The unconstrained bound $\Phi(\eta)$ is still very tight, since it is rapidly approached from below by the reverse coherent information computed at finite energy;

(2) The unconstrained bound $\Phi(\eta)$ remains tighter than other constrained bounds based on the squashed entanglement, even when $\bar{m}$ is of the order of a few photons.

Let us start with point (1). From Eq. (109), we see that the reverse coherent information associated with a lossy channel and a TMSV state is

$$I_{\text{RC}}(\bar{m}, \eta) = h(\bar{m}) - h\left[(1-\eta)\bar{m}\right], \tag{247}$$

which is obtained by setting $\mu = \bar{m} + 1/2$ in Eq. (109) and using the h-function of Eq. (95). In Supplementary Fig. 2, we see that $I_{\text{RC}}(\bar{m}, \eta)$ rapidly approaches the unconstrained upper bound $\Phi(\eta)$ already for $\bar{m} \simeq 1-5$ photons.

Let us now discuss point (2). We compare the unconstrained upper bound $\Phi(\eta)$ with the unconstrained TGW upper bound for the lossy channel [27]

$$K_{\text{TGW}}(\eta) = \log_2\left(\frac{1+\eta}{1-\eta}\right), \tag{248}$$

and its energy-constrained version

$$K_{\text{TGW}}(\eta, \bar{m}) = h\left[\frac{(1+\eta)\bar{m}}{2}\right] - h\left[\frac{(1-\eta)\bar{m}}{2}\right]. \tag{249}$$

(Note that the latter was just a partial result [27] used to derive the bound in Eq. (248) for $\bar{m} \to +\infty$).

In Supplementary Fig. 3 we clearly see that $\Phi(\eta)$ not only is tighter than $K_{\text{TGW}}(\eta)$ but also outperforms the constrained version $K_{\text{TGW}}(\eta, \bar{m})$ for all input energies down to one mean photon. This is certainly true in the regime of intermediate-long distances (> 25 km), where DV-QKD protocols have ideal performances at one mean photon per

channel use. At short distances (< 25 km), energy constraints do not really have so much practical value since we can efficiently use highly-modulated CV-QKD whose number of photons is high enough to approach the asymptotic infinite-energy behavior. In general, note that CV-QKD protocols with highly-modulated Gaussian states can be used at any distance. Their performance is not limited by the input energy, but critically depends on the efficiency of the output detection scheme and the quality of the data-processing (reconciliation efficiency).

Cost of classical communication

It is important to discuss the cost associated with the CCs. In fact, in order to achieve its performance, an optimal protocol will need a certain number of classical bits per channel use. Furthermore, the physical transmission of these bits is ultimately restricted by the speed of light. It is therefore essential to consider these aspects in order to translate a capacity, which is expressed in terms of target-bits (e.g. secret bits) per channel use, into a practical throughput, which is expressed in terms of target-bits per second. Consider the case of a bosonic lossy channel which is the most important for quantum optical communications.

By definition, an adaptive protocol is assisted by unlimited and two-way CCs. This is a very general formulation but it has an issue for practical applications: An adaptive protocol, which may be optimal in terms of target-bits per channel use, may have zero throughput in terms of target-bits per second, just due the fact that its implementation may require infinite rounds of feed-forward and feedback CCs in each channel use. The existence of such protocol is not excluded by the TGW bounds [27] of Eqs. (248) and (249), which are non-tight and do not have control on the CCs. By contrast, this problem is completed solved by our bound.

In fact, for any distillable channel $\mathcal{E}$ (e.g., bosonic lossy channel, quantum-limited amplifier, dephasing or erasure channel), the generic two-way capacity $\mathcal{C}(\mathcal{E})$ is equal to $D_1(\rho_{\mathcal{E}})$, which is the entanglement distillable from the Choi matrix of the channel by means of one-way CCs (forward, from Alice to Bob, or backward, from Bob to Alice). This means that an optimal protocol achieving the capacity is non-adaptive and it does not involve infinite rounds of CCs, but just a single round of forward or backward CCs.

For the specific case of a bosonic lossy channel, with transmissivity η, we find that an optimal key-generation protocol, achieving the repeaterless bound $K(\eta) = -\log_2(1-\eta)$, can be implemented by using backward CCs. In fact, as already discussed in Supplementary Note 4, an optimal key-generation protocol is the following: Alice prepares TMSV states $\Phi^{\mu}_{AA'}$ sending A' to Bob; Bob heterodynes each output mode, with outcome Y, and sends final CCs back to Alice; Alice measures all her modes A by means of an optimal coherent detection. Taking the limit for large μ, the key rate of the parties achieves the bound $K(\eta)$.

Because this is a Devetak-Winter rate (in reverse reconciliation), the amount of CCs required by the protocol (bits per channel use) is equal to the following conditional entropy [8]

$$\gamma_{\mathrm{CC}} := S(Y|A) = S(Y) - [S(A) - S(A|Y)], \tag{250}$$

where $S(Y) = H(Y)$ is the Shannon entropy of Bob's outcomes Y, while $S(A)$ and $S(A|Y)$ are the von Neumann entropies of Alice's reduced state ρ_A and conditional state $\rho_{A|Y}$. These quantities are all easily computable for any finite value of μ. By taking the limit for large μ, we derive the asymptotic cost

$$\gamma_{\mathrm{CC}}(\eta) = \frac{2\eta\log_2\pi + (2\eta-3)\log_2(3-2\eta) + 3\log_2 3}{2\eta} \leq \log_2(3\pi e) \approx 4.68 \text{ classical bits/use}, \tag{251}$$

where the latter bound is achieved for low transmissivities (long-distances), i.e., $\gamma_{\mathrm{CC}}(\eta \simeq 0) \simeq \log_2(3\pi e)$. According to Eq. (251), at any transmissivity η, Bob needs to send Alice no more than $\log_2(3\pi e)$ classical bits per channel use.

Consider a practical scenario where the rounds of the protocol are not infinite but yet a very large number, e.g., $n = 10^9$, so that the performance of such a large block of data is close to the asymptotic one. The amount of classical bits to be transmitted is linear in n, and the total cost is no larger than 4.68×10^9 bits, i.e., less than 1 gigabyte per block. Assuming the existence of a broadband classical channel between Alice and Bob, the extra time associated with the transmission of this classical overhead can be made negligible (for instance, it may happen at the beginning of the second large block of quantum communication). Assuming that the procedures of error correction and privacy amplification are also sufficiently fast within the block, then the final achievable throughput (secret-bits per second) will only depend on the capacity $K(\eta)$ (secret-bits per use) multiplied by the clock of the system (uses per second). Clearly, this is a simplified reasoning which does not consider other technical issues.

Supplementary Note 8. ADVANCES IN CHANNEL SIMULATION

The idea of channel simulation was originally introduced by Bennett-DiVincenzo-Smolin-Wootters (BDSW) [55] as a simple modification of the original teleportation protocol. Instead of performing standard teleportation by using a Bell state, one may consider an arbitrary mixed state as a resource. As a result, the effect of teleportation is not an identity map (transfer operator) but a noisy channel from the input to the output. BDSW introduced this teleportation-simulation argument to simulate DV channels that preserve the finite dimension d of the input Hilbert space $\mathcal{H}^d$, also known as the "tight" case [56]. Let us discuss the BDSW simulation in more detail.

Consider a mixed state σ of two qudits, A and B, both having dimension d, i.e., their joint Hilbert space is $\mathcal{H}_A^d \otimes \mathcal{H}_B^d$. The "teleportation channel" associated with the density operator $\sigma \in \mathcal{D}(\mathcal{H}_A^d \otimes \mathcal{H}_B^d)$ is the dimension-preserving quantum channel $\mathcal{T}_\sigma : \mathcal{D}(\mathcal{H}^d) \rightarrow \mathcal{D}(\mathcal{H}^d)$, which is given by teleporting an input d-dimensional qudit by using the resource state σ. The procedure goes as follows. Alice measures qudit A and input qudit a in a Bell detection, whose outcome $k \in \{0, \ldots, d^2-1\}$ is associated with a qudit Pauli unitary U_k. This detection projects Bob's qudit B onto a k-dependent state. Once the outcome k is communicated to Bob, he applies the Pauli correction U_k^{-1} to qudit B thus retrieving the final state on the output qudit b. The average over all outcomes k defines the teleportation channel $\mathcal{T}_\sigma$ from the states of a to those of b.

BDSW [55, Section V] also recognized that a Pauli channel $\mathcal{E}$ (there called "generalized depolarizing channel") can be simulated by teleporting over its Choi matrix $\rho_\mathcal{E}$, so that $\mathcal{E} = \mathcal{T}_{\rho_\mathcal{E}}$. This particular case was later re-considered in ref. [57] as a property of mutual reproducibility between mixed states and quantum channels. In a few words, we may store a channel $\mathcal{E}$ into its Choi matrix $\rho_\mathcal{E}$ (by sending half of an EPR state), and then recover the channel back by performing teleportation over $\rho_\mathcal{E}$. At this point, a natural question to ask is the following:

Can we generate other DV channels (beyond Pauli) using the teleportation-simulation of BDSW [55, Section V]?

The answer is *no*. In fact, ref. [58] showed that the standard teleportation protocol (based on Bell detection and Pauli corrections) performed over an arbitrary $d \times d$ state σ can only simulate a quantum channel of the form

$$\mathcal{T}_\sigma(\rho) = \sum_{ab} \mathrm{Tr}(\sigma M_{ab})\; U_{(-a)b}^\dagger\; \rho\; U_{(-a)b}\ , \tag{252}$$

where $M_{ab} := (U_{ab} \otimes I)^\dagger \left|\Phi\right\rangle \left\langle\Phi\right| (U_{ab} \otimes I)$ are the POVM elements of the Bell detection (with $\left|\Phi\right\rangle$ being a d-dimensional Bell state), and U_{ab} are Pauli operators. This is clearly a d-dimensional Pauli channel. The possibility to generate other DV channels relies on a stronger modification of the original teleportation protocol, where we allow for more general quantum operations [56, 59] and also for the possibility of varying the dimension of the Hilbert space. Recently, ref. [60] considered a generalization of the teleportation-simulation argument for DV channels, using tools from ref. [56] and moving important steps into the study of teleportation covariance (see also ref. [61]). Similarly, ref. [62] moved the first steps in the simulation of single-mode Gaussian channels by using Gaussian resources and the standard CV teleportation protocol [63].

In our paper we provide the most general and rigorous formulation. In fact, we remove all the assumptions regarding the dimension of the quantum systems which may also vary through the channel. Thus we may tele-simulate, DV channels, CV channels and even hybrid channels, i.e., mappings between DVs and CVs. More generally, our simulation is not limited to teleportation-LOCCs (i.e., Bell detection and unitary corrections), but considers completely general LOCCs which may also be asymptotic, i.e., defined as suitable sequences. Furthermore, the simulating LOCCs may also include portions of the channels (i.e., we may decompose a channel $\mathcal{E}$ as $\mathcal{E}_2 \circ \tilde{\mathcal{E}} \circ \mathcal{E}_1$ and include $\mathcal{E}_1$ and $\mathcal{E}_2$ in the LOCCs). For all these reasons, we may simulate *any* quantum channel at any dimension. As discussed in the main text, the best case is when the simulation can be done directly on the channel's Choi matrix. To identify this case we introduce the criterion of teleportation-covariance at any dimension, finite or infinite.

Note that ours is the most general simulation to be used in quantum/private communication, which is a setting where two remote parties can only apply LOCCs. In this regard, it is different and more precise than the channel simulation realized by using a deterministic version [64] of a programmable quantum gate array (PQGA) [65, 66]. This is also known as "quantum simulation" [67] and considers the simulation of "programmable channels" by means of *joint* operations. A programmable channel is defined as a (finite-dimensional) channel $\mathcal{E}$ that can be simulated as

$$\mathcal{E}(\rho) = \Omega(\rho \otimes \sigma_\mathcal{E}), \tag{253}$$

for a universal joint quantum operation Ω and some programme state $\sigma_\mathcal{E}$. This clearly fails to catch the LOCC structure which is essential for protocols of quantum/private communication. Furthermore, this type of simulation has not been developed into an asymptotic version (via CV teleportation), which is clearly needed for the representation of bosonic channels. Finally, the universal character of the operation Ω restricts the class of channels that can be simulated

(universality implies that we cannot include portions of the channel in the operation, missing a procedure that allows one to simulate all channels). Our LOCC-simulation of channels solves all these issues.

To conclude, we give the timeline of the previous main contributions before our formulation of channel simulation:

1996 BDSW introduces the teleportation-simulation of Pauli channels [55, Section V]

1997 Nielsen and Chuang introduce the PQGA [65]

1998 Braunstein and Kimble design a realistic protocol for CV teleportation [63]

1999 Horodeckis consider the BDSW simulation for channel reproducibility [57]

2001 Bowen and Bose show that the BDSW simulation can only simulate Pauli channels [58]

2001 Werner discusses generalized teleportation protocols [56]

2008 Ji et al. use a deterministic PQGA to simulate certain DV channels in the context of quantum metrology [64]

2009 Niset et al. simulate Gaussian channels in the context of one-way Gaussian entanglement distillation [62]

2015 Leung and Matthews first discuss teleportation covariance in connection with the simulation of DV channels [60]

2015-6 The present paper rigorously generalizes the idea of teleportation-simulation to CV systems (bosonic channels). More generally, it introduces the LOCC-simulation of any channel at any dimension (including asymptotic simulations), and identifies the criterion of teleportation-covariance at any dimension (finite or infinite).

Supplementary Note 9. ADVANCES IN PROTOCOL REDUCTION

Teleportation stretching is a general method to reduce adaptive protocols into corresponding block protocols achieving exactly the same original task. Furthermore, it may be applied to any channel at any dimension, finite or infinite, thanks to our development of the tool of channel simulation (see Supplementary Note 8). In terms of reduction of protocols, a precursory but very restricted argument was given in BDSW [55, Section V]. Here we discuss this preliminary argument and we point out the main and non-trivial advances brought by our general formulation.

BDSW showed how to transform a quantum communication (QC) protocol, through a finite-dimensional Pauli channel $\mathcal{E}$, into an entanglement distillation (ED) protocol, implemented over mixed states σ. The connection was established by interpreting $\mathcal{E}$ as the teleportation channel generated by σ (which can be taken to be a Choi-matrix for a Pauli channel). This allowed them to prove

$$Q_1(\mathcal{E}) \leq D_1(\sigma), \tag{254}$$

for protocols based on 1-way CCs. They also realized that the argument could be applied to transform QC protocols based on 2-way CCs, so that they implicitly extended the previous result to the following inequality

$$Q_2(\mathcal{E}) \leq D_2(\sigma). \tag{255}$$

An explicit proof for Eq. (255) is reported in Supplementary Fig. 4.

Let us now compare teleportation stretching with the precursory BDSW argument. We identify a number of non-trivial differences and advances.

1. **Finite-size decomposition and connection with REE**. The BDSW reduction argument was formulated in an asymptotic fashion, i.e., for large n, which is sufficient to prove Eqs. (254) and (255). Teleportation stretching regards any n, and gives the finite-size decomposition of the output $\bar{\Lambda}(\sigma^{\otimes n})$ for a trace-preserving LOCC $\bar{\Lambda}$ collapsing all the adaptive LOCCs. The finite-size decomposition $\bar{\Lambda}(\sigma^{\otimes n})$ could have not been exploited by BDSW, due to missing tools for the simplification of $\bar{\Lambda}$. This simplification is today achieved by combining teleportation stretching with the REE, which is the key insight giving applicability to the technique.

2. **Task preserving**. The BDSW reduction argument was specifically formulated to transform a QC protocol into an ED protocol, therefore changing the task of the original protocol. In teleportation stretching, we maintain the task. In the example of Supplementary Fig. 4b, we show the different re-organization of the quantum operations of the QC protocol. Teleportation stretching would directly reduce the output of the QC protocol as follows $\rho_b(\mathcal{E}^{\otimes n}) = \bar{\Lambda}(\sigma^{\otimes n})$, for a trace-preserving LOCC $\bar{\Lambda}$ which is not connected with ED but collapses the preparation $|\varphi^{(m)}\rangle\langle\varphi^{(m)}|$, the encoding/decoding maps, and the teleportation operations.

3. **Any task**. Maintaining the task and output of the original protocol is crucial, because the reduction can now be applied to any kind of adaptive protocol, not just quantum communication, but any other task, including key generation (considered in this paper) and parameter estimation/channel discrimination (considered in ref. [68]). This aspect is also important in order to extend the procedure to more complex scenarios, from two-way quantum communication to the presence of quantum repeaters in arbitrary network topologies [69].

4. **Any channel and dimension**. The BDSW reduction argument was given for the restricted class of Pauli channel. Teleportation stretching is formulated for any channel at any dimension (finite or infinite). This is non-trivial because it involves the use of asymptotic simulations for fundamental channels such as the bosonic Gaussian channels and the amplitude damping channel. In general, we may write an output decomposition of the type $\lim_\mu \bar{\Lambda}_\mu(\sigma^{\mu\otimes n})$ for sequences of trace-preserving LOCCs $\bar{\Lambda}_\mu$ and resource states σ^μ.

In the literature, we can also find another type of adaptive-to-block reduction, which is based on the use of a deterministic PQGA. It is known that a PQGA can simulate an arbitrary unitary or channel in a probabilistic way [65]. However, as discussed in Supplementary Note 8, one may also define a class of programmable channels for which the PQGA works deterministically: These are (finite-dimensional) channels $\mathcal{E}$ that can be simulated as in Eq. (253) for a universal generally-joint quantum operation Ω and a programme state $\sigma_{\mathcal{E}}$. It is easy to check that, in a protocol, this "quantum simulation" [67] leads to an output decomposition of the type $Q(\sigma_{\mathcal{E}}^{\otimes n})$, where Q is a joint quantum operation for Alice and Bob. Clearly this is not suitable for quantum/private communication, where the parties are restricted to LOCCs and, therefore, both the channel simulation and the adaptive-to-block reduction must maintain the LOCC structure of the original protocol. Furthermore, it lacks an asymptotic formulation which is needed for bosonic channels and also the flexibility to include portions of the channels in the simulating operations (these are elements introduced by our approach). It is worth to mention that the quantum simulation plays a role for the simplification of adaptive protocols in quantum metrology and channel discrimination, where the parties are close (they are indeed the same entity) and may therefore apply joint unitaries and joint measurements. See refs. [68, 70].

Supplementary Note 10. ADVANCES IN BOUNDING TWO-WAY CAPACITIES

By simulating Pauli channels, BDSW showed how to reduce a quantum communication protocol into an entanglement distillation protocol. By combining this argument with an opposite implication, they were able to show that, for a Pauli channel $\mathcal{E}$, one may write $Q_1(\mathcal{E}) = D_1(\rho_{\mathcal{E}})$, which was implicitly extended to

$$Q_2(\mathcal{E}) = D_2(\rho_{\mathcal{E}}). \tag{256}$$

The latter result is not exploitable for computing the two-way quantum capacity Q_2 unless one identifies simple (and tight) upper bounds for D_2. Such elements were missing in 1996 but today we can exploit powerful tools.

Using today's knowledge, the simplest approach is to combine Eq. (256) with the fact that $D_2(\rho_{\mathcal{E}}) \leq K(\rho_{\mathcal{E}})$ (since an ebit is a particular type of secret-bit) and the REE upper bound on the distillable key of quantum states [11], so that $K(\rho_{\mathcal{E}}) \leq E_{\mathrm{R}}^{\infty}(\rho_{\mathcal{E}})$. All this leads us to write

$$Q_2(\mathcal{E}) \leq E_{\mathrm{R}}^{\infty}(\rho_{\mathcal{E}}) \leq E_{\mathrm{R}}(\rho_{\mathcal{E}}) \ . \tag{257}$$

Our work shows the bound of Eq. (257) for any finite-dimensional Choi-stretchable channel. In particular, we show that the single-letter REE bound of Eq. (257) is tight for dephasing and erasure channels.

The next non-trivial generalization is moving from quantum to *private* communication. In this regard, the notions of private capacities [71] and private states [11, 12] were available well after 1996. Note that we may consider the secret-key capacity K, which is the number of secret bits which are distributed between the parties (via adaptive protocols), and the two-way private capacity P_2, which is the maximum rate at which classical messages can be securely encoded and transmitted [71]. Because of the unlimited two-way CCs and the one-time pad, we have $P_2 = K$. For a finite-dimensional Choi-stretchable channel $\mathcal{E}$, it is easy to write the equivalence

$$P_2(\mathcal{E}) = K(\mathcal{E}) = K(\rho_{\mathcal{E}}) \ . \tag{258}$$

The simplest way to show this is to apply teleportation stretching to reduce adaptive key-generation protocols, which leads to $K(\mathcal{E}) = K(\rho_{\mathcal{E}})$ as in Proposition 6 of our main text. An alternate way is to show $P_2(\mathcal{E}) = K(\rho_{\mathcal{E}})$ by means of a suitable extension of the BDSW reduction argument. In fact, for a finite-dimensional Choi-stretchable channel, we may transform a protocol of private communication [71] through $\mathcal{E}$ into a protocol of key-distillation [11, 12] over the Choi matrix $\rho_{\mathcal{E}}$, so that $P_2(\mathcal{E}) \leq K(\rho_{\mathcal{E}})$. The latter bound is achievable by a protocol where Alice transmits part

of Bell states, so that the parties distill a key from the output Choi matrices, which is then used to send the message via the one-time pad. Note that these extensions from quantum to private communication, and from entanglement to key distillation were not available in 1996, which is why Eq. (258) can only be written today. At the same time, it is surprising that Eq. (258) was never written before our work, with many of the tools being available since 2005.

Now it is very important to observe that both Eqs. (257) and (258) cannot be used to investigate the most important setting for quantum/private communication, which is the bosonic one. Furthermore, they miss to provide single-letter bounds for other DV channels which involve asymptotic simulations (e.g., amplitude damping). For these important reasons, it is necessary to develop a general theory which is dimension-independent and applicable to channels of any dimension, finite or infinite. This is the main content of our Theorem 5 in the main text. This states that, for any channel $\mathcal{E}$ stretchable into a resource state σ (even asymptotically), we may write

$$\mathcal{C}(\mathcal{E}) \leq E_{\mathrm{R}}^{\infty}(\sigma) \leq E_{\mathrm{R}}(\sigma), \tag{259}$$

where $\mathcal{C}(\mathcal{E})$ is any among the two-way capacities $Q_2(\mathcal{E}) = D_2(\mathcal{E}) \leq P_2(\mathcal{E}) = K(\mathcal{E})$. In particular, for a Choi stretchable channel ($\sigma = \rho_{\mathcal{E}}$), we have

$$\mathcal{C}(\mathcal{E}) \leq E_{\mathrm{R}}^{\infty}(\rho_{\mathcal{E}}) \leq E_{\mathrm{R}}(\rho_{\mathcal{E}}). \tag{260}$$

Recall that the proof of Eq. (259) relies on the following steps:

- First the derivation of the REE bound $\mathcal{C}(\mathcal{E}) \leq E_{\mathrm{R}}^{\bigstar}(\mathcal{E})$ for any channel $\mathcal{E}$ at any dimension (weak converse theorem)
- Second, the adaptive-to-block reduction by teleportation stretching at any dimension, which decomposes the output of an arbitrary adaptive protocol into $\bar{\Lambda}(\sigma^{\otimes n})$ or a suitable asymptotic form.

Because the REE is a functional which is monotonic under trace-preserving LOCCs and subadditive over tensor products, we may then derive Eq. (259). It is clear that this procedure can be adapted to simplify any functional which is monotonic under LOCCs, which includes the Rains bound [72, 73] and entanglement monotones.

SUPPLEMENTARY DISCUSSION

Schematic summary of our key findings

(1) We have designed an **adaptive-to-block reduction** method which reduces any adaptive protocol for quantum communication, entanglement distribution and key generation to the computation of a single-letter quantity. This is possible by combining the following two main ingredients:

(1.1) Channel's REE. We have extended the notion of relative entropy of entanglement (REE) from states to channels. In particular, we have shown that the two-way capacity $\mathcal{C}(\mathcal{E})$ of any channel $\mathcal{E}$ is upperbounded by a suitably-defined REE bound $E_{\mathrm{R}}^{\bigstar}(\mathcal{E})$.

(1.2) LOCC simulation and teleportation stretching. We have introduced the most general form of simulation of a quantum channel within a quantum/private communication scenario. This is based on arbitrary LOCCs (even asymptotic) and can be used to stretch an arbitrary channel $\mathcal{E}$ into a resource state σ. By exploiting this simulation, we have shown how to reduce an adaptive protocol (achieving an arbitrary task) into a block form, so that its output can be decomposed as $\bar{\Lambda}(\sigma^{\otimes n})$ for a trace-preserving LOCC $\bar{\Lambda}$. This is valid at any dimension (finite or infinite) and can be extended to more complex communication scenarios.

Thus, the insight of our entire reduction method is the combination of (1.1) and (1.2). 'REE+teleportation stretching' allows us to exploit the properties of the REE (monotonicity, subadditivity) and simplify $E_{\mathrm{R}}^{\bigstar}(\mathcal{E})$ into a single-letter quantity so that we may write $\mathcal{C}(\mathcal{E}) \leq E_{\mathrm{R}}(\sigma)$ for any σ-stretchable channel. This is valid at any dimension.

(2) Teleportation covariance. At any dimension (finite or infinite), we have identified a simple criterion (teleportation covariance) which allows us to find those channels which are stretchable into their Choi matrices (Choi-stretchable channels). For these channels, we may write $\mathcal{C}(\mathcal{E}) \leq E_{\mathrm{R}}(\rho_{\mathcal{E}})$, with the latter being the entanglement flux of the channel.

(3) Tight bounds and two-way capacities. We have shown that the entanglement flux is the tightest upper bound for the two-way capacities of many quantum channels at any dimension, including Pauli, erasure and bosonic Gaussian channels. In particular, we have established the two-way capacities (Q_2, D_2 and K) of the bosonic lossy channel, the quantum-limited amplifier, and the dephasing channel in arbitrary finite dimension, plus the secret key capacity K of the erasure channel in arbitrary finite dimension. All these capacities have extremely simple formulas. For our calculations we have derived a simple formula for the relative entropy between two arbitrary Gaussian states.

(4) Fundamental rate-loss tradeoff. We have finally characterized the rate-loss tradeoff affecting quantum optical communications, so that the rate of repeaterless QKD is restricted to 1.44η bits per channel use at long distances. This rate is achievable with one-way CCs and provides the maximum throughput of a point-to-point QKD protocol.

Recent developments in quantum and private communications

Repeater-assisted capacities and multi-hop networks

As also mentioned in the discussion of the main text, an important generalization of the results has been achieved in ref. [69] with the study and determination of repeater-assisted capacities in the presence of unlimited two-way CCs. Ref. [69] establishes the ultimate rates for transmitting quantum information, distributing entanglement and secret keys in repeater-assisted quantum communications, under the most fundamental decoherence models for both discrete and continuous variable systems, including lossy channels, quantum-limited amplifiers, dephasing and erasure channels. These capacities are derived considering the most general adaptive protocols of quantum and private communication between the two end-points of a repeater chain and, more generally, of an arbitrarily-complex quantum network or internet, where systems may be routed though single or multiple paths. Methodology combines tools from quantum information and classical network theory. Converse results are derived by introducing a tensor-product representation for a quantum communication network, where quantum channels are replaced by their Choi matrices. Exploiting this representation and suitable entanglement cuts of the network, one can upperbound the end-to-end capacities by means of the relative entropy of entanglement. Achievability of the bounds is proven by combining point-to-point quantum communications with classical network algorithms, so that optimal routing strategies are found by determining the widest path and the maximum flow in the network. In this way, ref. [69] extends both the widest path problem and the max-flow min-cut theorem from classical to quantum communications.

Single-hop networks (broadcast, multiple-access and interference channels)

Ref. [74] investigates the maximum rates for transmitting quantum information, distributing entanglement and secret keys in a single-hop multipoint network, with the assistance of unlimited two-way classical communication among all the parties. Ref. [74] first considers a sender directly communicating with an arbitrary number of receivers, so called quantum broadcast channel. In this case, it provides a simple analysis in the bosonic setting considering quantum broadcasting through a sequence of beamsplitters. This specific case has been also investigated in ref. [75] where the use of our method (REE+teleportation stretching) has led to the determination of the capacity region of the lossy broadcast channel. Then, ref. [74] also considers the multipoint setting where an arbitrary number of senders directly communicate with a single receiver, so called quantum multiple-access channel. Finally, ref. [74] studies the general case of a quantum interference channel where an arbitrary number of senders directly communicate with an arbitrary number of receivers. Upper bounds are formulated for quantum systems of arbitrary dimension, so that they can be applied to many different physical scenarios involving multipoint quantum and private communication.

Improving the lower bound for the thermal-loss channel

It remains an open problem to determine the two-way capacities of several channels, most notably that of the thermal-loss channel $\mathcal{E}_{\text{loss}}(\eta, \bar{n})$. Here we have shown lower- and upper-bounds in Eq. (126). Recently, ref. [76] has studied the specific case of the secret-key capacity $K(\eta, \bar{n})$ of this channel investigating a region where the lower-bound given by the reverse coherent information can be beaten. This is possible by resorting to a Gaussian QKD protocol based on trusted-noise detection. However, the improved lower bound is still far from closing the gap.

Improved upper bounds based on the squashed entanglement and secret-key capacity of the erasure channel

Note that the first version of our paper appeared on the arXiv in October 2015 [77]. It originally contained the main result for the bosonic lossy channels. The other results for DV and CV channels were given in a second paper, uploaded on the arXiv in mid December 2015 [78]. These two papers were later merged into a single contribution, which is the present manuscript.

In late November 2015, one month after our first arXiv version, another manuscript appeared on the arXiv by Goodenough *et al.* [28]. This is a very interesting paper that improves the upper bounds of ref. [27] based on the squashed entanglement. As is clear from our main text, these improved bounds are still larger than ours based on the REE. However there are two notable exceptions: the amplitude damping channel and the erasure channel. For the amplitude damping channel, ref. [28] led us to improve our previous results and to find the tightest known upper-bound based on the squashed entanglement, which is the one given in Eq. (232). Regarding the erasure channel, the REE and the squashed entanglement lead to the same upper bound, so that both methods are sufficient to determine the secret-key capacity of this channel.

In our main text, we acknowledge the independent derivation of ref. [28] for the secret-key capacity of the erasure channel. This is independent because of the completely different method. It is simultaneous because it has been achieved in a short time window between our first [77] and second [78] arXiv papers. Goodenough *et al.* [28] first wrote their upper bound for the erasure channel without making the crucial observation that it was tight. They then realized this important fact after seeing our updated results on the arXiv two weeks later [78], where we first explicitly claimed the secret-key capacity of the erasure channel. In a later update of their manuscript (arXiv version 2, April 2016) they then remarked the tightness and claimed to have found the capacity too. In agreement with these authors, we have therefore decided to credit each other for the independent derivation of the secret-key capacity of the erasure channel.

Further remarks

Simulation and stretching of bosonic channels

In March 2016, several months after our manuscript was available on the arXiv, an author uploaded a paper [79] discussing some mathematical aspects associated with our treatment of teleportation stretching with bosonic channels. Let us briefly give some background before clarifying that these mathematical aspects were already taken into account and addressed in our arXiv version 2 of December 2015 [80].

Teleportation stretching of bosonic channels involves the use of an asymptotic CV EPR state Φ, defined as the limit of TMSV states Φ^μ. As a consequence, we have to consider the following steps: (i) We first perform an imperfect stretching of the protocol based on a finite-energy TMSV state Φ^μ; (ii) we compute the relevant functionals on the finite-energy decomposition of the output; and (iii) we take the infinite-energy limit $\mu \to +\infty$ on the final result. This is actually a standard procedure in any calculus with a delta function, which is implicitly meant to be a limit of test functions. This is also why the Vaidman teleportation protocol [81] (based on an asymptotic delta-like CV EPR state) has to be implicitly replaced by the Braunstein-Kimble protocol [63], where the resource state is a TMSV state Φ^μ and the infinite-energy limit is computed at the end on the fidelity.

Such a basic argument was already present in our earlier arXiv versions. Already in December 2015 [80] we stated that, for bosonic channels, one needs to relax the condition of infinite energy and replace the asymptotic CV EPR state Φ by a sequence of TMSV states Φ^μ, defining a sequence of Choi-approximating states $\rho^\mu_{\mathcal{E}} := \mathcal{I} \otimes \mathcal{E}(\Phi^\mu)$. The latter states are then used to compute the relative entropy of entanglement before taking the limit for large μ; see Eq. (9) and corresponding text of ref. [80]. Therefore, our treatment of bosonic channels was already rigorous and correct well before ref. [79]. However, we have also realized that these non-trivial steps were too implicit. For this reason, we have decided to fully expand the specific treatment of bosonic channels in more recent arXiv versions of our manuscript. Furthermore, in order to be completely rigorous, we have also accounted for the fact that the CV Bell detection also needs to be approximated by a suitable limit of finite-energy measurements.

Shield system

In earlier arXiv versions of our manuscript, we proved our weak converse theorem by exploiting an (at most) exponential growth of the dimensionality of the shield system in the private state. This corresponds to the first proof in Supplementary Note 3. This assumption on the shield size is correct and fully justified by the argument of refs. [14, 15] which may be applied to both DV and CV channels, as presented in Lemma 4 of Supplementary Note 3 for the sake of completeness. Despite the correctness of this approach, in later arXiv versions we have also provided two additional proofs, alternative but essentially equivalent to the first one (with exactly the same conclusions). Our second proof relies on an exponential increase of the mean number of photons in the private state, while our third proof is independent from the shield system. See Supplementary Note 3 for full details. It is clear that these proofs are all *complete proofs* which do not need further confirmation or validation by follow-up works.

Supplementary References

[1] Holevo, A. S. Entanglement-assisted capacity of constrained channels. *Probab. Theory Appli.* **48**, 243–255 (2004).

[2] D'Ariano, G. M., Kretschmann, D., Schlingemann, D. & Werner, R. F. Reexamination of quantum bit commitment: The possible and the impossible. *Phys. Rev. A* **76**, 032328 (2007).

[3] Pirandola, S. *et al.* Advances in quantum teleportation. *Nature Photon.* **9**, 641-652 (2015).

[4] Schumacher, B. & Nielsen, M. A. Quantum data processing and error correction. *Phys. Rev. A* **54**, 2629–2635 (1996).

[5] Lloyd, S. Capacity of the noisy quantum channel. *Phys. Rev. A* **55**, 1613–1622 (1997).

[6] García-Patrón, R., Pirandola, S., Lloyd, S. & Shapiro, J. H. Reverse coherent information. *Phys. Rev. Lett.* **102**, 210501 (2009).

[7] Pirandola, S., R. García-Patrón, Braunstein, S. L. & Lloyd, L. Direct and reverse secret-key capacities of a quantum channel. *Phys. Rev. Lett.* **102**, 050503 (2009).

[8] Devetak, I. & Winter, A. Distillation of secret key and entanglement from quantum states. *Proc. R. Soc. A* **461**, 207–235 (2005).

[9] Winter, A. Tight uniform continuity bounds for quantum entropies: conditional entropy, relative entropy distance and energy constraints. *Commun. Math. Phys.* **347**, 291–313 (2016).

[10] Weedbrook, C. *et al.* Gaussian quantum information. *Rev. Mod. Phys.* **84**, 621–669 (2012).

[11] Horodecki, K., Horodecki, M., Horodecki, P. & Oppenheim, J. Secure key from bound entanglement. *Phys. Rev. Lett.* **94**, 160502 (2005).

[12] Horodecki, K., Horodecki, M., Horodecki, P. & Oppenheim, J. General Paradigm for Distilling Classical Key From Quantum States. *IEEE Trans. Inf. Theory* **55**, 1898–1929 (2009).

[13] Renes, J. & Smith, G. Noisy Processing and Distillation of Private Quantum States. *Phys. Rev. Lett.* **98**, 020502 (2007).

[14] Christiandl, M., Ekert, A., Horodecki, M., Horodecki, P., Oppenheim, J. & Renner, R. Unifying classical and quantum key distillation. *Lecture Notes in Computer Science* **4392**, 456–478 (2007). See also preprint at https://arxiv.org/abs/quant-ph/0608199v3 (2006).

[15] Christiandl, M., Schuch, N. & Winter, A. Entanglement of the antisymmetric state. *Comm. Math. Phys.* **311**, 397–422 (2012).
[16] Vedral, V. The role of relative entropy in quantum information theory. *Rev. Mod. Phys.* **74**, 197–234 (2002).
[17] Donald, M. J. & Horodecki, M. Continuity of Relative Entropy of Entanglement. *Phys. Lett. A* **264**, 257–260 (1999).
[18] Synak-Radtke, B. & Horodecki, M. On asymptotic continuity of functions of quantum states. *J. Phys. A: Math. Gen.* **39**, L423–L437 (2006).
[19] Holevo, A. Quantum Systems, Channels, Information: A Mathematical Introduction (De Gruyter, Berlin-Boston, 2012).
[20] Alicki, R. & Fannes, M. Continuity of conditional quantum mutual information. *J. Phys. A: Math. Gen.* **37**, L55–L57 (2004).
[21] Arvind, B. Dutta, N. Mukunda, & R. Simon. The real symplectic groups in quantum mechanics and optics. *Pramana* **45**, 471–497 (1995).
[22] Banchi, L., Braunstein, S.L. & Pirandola, S. Quantum fidelity for arbitrary Gaussian states. *Phys. Rev. Lett.* **115**, 260501 (2015).
[23] Pirandola, S., Serafini, A. & Lloyd, S. Correlation matrices of two-mode bosonic systems. *Phys. Rev. A* **79**, 052327 (2009).
[24] Pirandola, S. Entanglement Reactivation in Separable Environments. *New J. Phys.* **15**, 113046 (2013).
[25] Pirandola, S., Spedalieri, G., Braunstein, S. L., Cerf, N. J. & Lloyd, S. Optimality of Gaussian discord. *Phys. Rev. Lett.* **113**, 140405 (2014).
[26] Holevo, A. S. & Werner, R. F. Evaluating capacities of bosonic Gaussian channels. *Phys. Rev. A* **63**, 032312 (2001).
[27] Takeoka, M., Guha, S. & Wilde, M. M. Fundamental rate-loss tradeoff for optical quantum key distribution. *Nat. Commun.* **5**, 5235 (2015).
[28] Goodenough, K., Elkouss, D. & Wehner, S. Assessing the performance of quantum repeaters for all phase-insensitive Gaussian bosonic channels. Preprint at https://arxiv.org/abs/1511.08710v1 (2015).
[29] Pirandola, S. Quantum discord as a resource for quantum cryptography. *Sci. Rep.* **4**, 6956 (2014).
[30] Modi, K. *et al.* The classical-quantum boundary for correlations: Discord and related measures. *Rev. Mod. Phys.* **84**, 1655–1707 (2012).
[31] Adesso, G. & Datta, A. Quantum versus classical correlations in Gaussian states. *Phys. Rev. Lett.* **105**, 030501 (2010).
[32] Giorda, P. & Paris, M.G.A. Gaussian Quantum Discord. *Phys. Rev. Lett.* **105**, 020503 (2010).
[33] Wolf, M. M., Pérez-García, D. & Giedke, G. Quantum Capacities of Bosonic Channels. *Phys. Rev. Lett.* **98**, 130501 (2007).
[34] Bennett, C. H., DiVincenzo, D. P., & Smolin, J. A. Capacities of Quantum Erasure Channels. *Phys. Rev. Lett.* **78**, 3217–3220 (1997).
[35] Vedral, V., Plenio, M. B., Rippin, M. A. & Knight, P. L. Quantifying Entanglement. *Phys. Rev. Lett.* **78**, 2275–2279 (1997).
[36] Devetak, I. & Shor, P.W. The Capacity of a Quantum Channel for Simultaneous Transmission of Classical and Quantum Information, *Commun. Math. Phys.* **256**, 287–303 (2005).
[37] Synak, B., Horodecki, K & Horodecki, M. Bounds on localisable information via semidefinite programming. *J. Math. Phys.* **46**, 082107 (2005).
[38] Fukuda, M. & Holevo, A. S. On Weyl covariant channels. Preprint at https://arxiv.org/abs/quant-ph/0510148 (2005).
[39] Pirandola, S., Mancini, S., Braunstein, S. L. & Vitali, D. Minimal qudit code for a qubit in the phase-damping channel. *Phys. Rev. A* **77**, 032309 (2008).
[40] Takeoka, M., Guha, S. & Wilde, M. M. The Squashed Entanglement of a Quantum Channel. *IEEE Transactions on Information Theory* **60**, 4987–4998 (2014).
[41] Weedbrook, C. *et al.* Quantum Cryptography Without Switching. *Phys. Rev. Lett.* **93**, 170504 (2004).
[42] Grosshans, F. & Grangier, P. Continuous Variable Quantum Cryptography Using Coherent States. *Phys. Rev. Lett.* **88**, 057902 (2002).
[43] Grosshans, F. *et al.* Quantum key distribution using gaussian-modulated coherent states. *Nature* **421**, 238–241 (2003).
[44] Pirandola, S. *et al.* High-rate measurement-device-independent quantum cryptography. *Nature Photon.* **9**, 397–402 (2015).
[45] Pirandola, S. *et al.* Reply to 'Discrete and continuous variables for measurement-device-independent quantum cryptography'. *Nature Photon.* **9**, 773–775 (2015).
[46] Ottaviani, C., Spedalieri, G., Braunstein, S.L. & Pirandola, S. Continuous-variable quantum cryptography with an untrusted relay: Detailed security analysis of the symmetric configuration. *Phys. Rev. A* **91**, 022320 (2015).
[47] Spedalieri, G. *et al.* Quantum cryptography with an ideal local relay. *Proceedings of the SPIE Security + Defence 2015 conference on Quantum Information Science and Technology*, Toulouse, France (21-24 September 2015). Paper 96480Z. See also Preprint at https://arxiv.org/abs/1509.01113 (2015).
[48] Pirandola, S., Mancini, S., Lloyd, S. & Braunstein, S. L. Continuous-variable quantum cryptography using two-way quantum communication. *Nature Phys.* **4**, 726–730 (2008).
[49] Ottaviani, C. & Pirandola S. General immunity and superadditivity of two-way Gaussian quantum cryptography. *Sci. Rep.* **6**, 22225 (2016).
[50] Bennett, C. H. & Brassard, G. Quantum cryptography: Public key distribution and coin tossing. *Proc. IEEE International Conf. on Computers, Systems, and Signal Processing*, Bangalore, pp. 175–179 (1984).
[51] Scarani, V. *et al.* The security of practical quantum key distribution. *Rev. Mod. Phys.* **81**, 1301–1350 (2009).
[52] Hwang, W.-Y. Quantum Key Distribution with High Loss: Toward Global Secure Communication. *Phys. Rev. Lett.* **91**, 057901 (2003).
[53] Braunstein, S. L. & Pirandola, S. Side-channel-free quantum key distribution. *Phys. Rev. Lett.* **108**, 130502 (2012).
[54] Lo, H.-K., Curty, M. & Qi, B. Measurement-Device-Independent Quantum Key Distribution. *Phys. Rev. Lett.* **108**, 130503 (2012).

[55] Bennett, C. H., DiVincenzo, D. P., Smolin, J. A. & Wootters, W. K. Mixed-state entanglement and quantum error correction. *Phys. Rev. A* **54**, 3824–3851 (1996).
[56] Werner, R. F. All teleportation and dense coding schemes. *J. Phys. A* **34**, 7081–7094 (2001).
[57] Horodecki, M., Horodecki, P. & Horodecki, R. General teleportation channel, singlet fraction, and quasidistillation. *Phys. Rev. A* **99**, 1888–1898 (1999).
[58] Bowen, G. & Bose, S. Teleportation as a Depolarizing Quantum Channel, Relative Entropy and Classical Capacity. *Phys. Rev. Lett.* **87**, 267901 (2001).
[59] Albeverio, S., Fei, S.-M. & Yang, W.-L. Optimal teleportation based on Bell measurements. *Phys. Rev. A* **66**, 012301 (2002).
[60] Leung, D. & Matthews, W. On the power of ppt-preserving and nonsignalling codes. *IEEE Transactions on Information Theory* **61**, 4486–4499 (2015).
[61] Müller-Hermes, A. Transposition in Quantum Information Theory. *Master Thesis, Technische Universität München* (2012).
[62] J. Niset, J., Fiurasek, J. & Cerf, N. J. No-go theorem for Gaussian quantum error correction. *Phys. Rev. Lett.* **102**, 120501 (2009).
[63] Braunstein, S. L. & Kimble, H. J. Teleportation of continuous quantum variables. *Phys. Rev. Lett.* **80**, 869–872 (1998).
[64] Ji, Z., Wang, G., Duan, R., Feng, Y. & Ying, M. Parameter estimation of quantum channels. *IEEE Trans. Inform. Theory* **54**, 5172–5185 (2008).
[65] Nielsen, M. A. & Chuang, I. L. Programmable Quantum Gate Arrays. *Phys. Rev. Lett.* **79**, 321–324 (1997).
[66] Ishizaka, S. & Hiroshima, T. Asymptotic Teleportation Scheme as a Universal Programmable Quantum Processor. *Phys. Rev. Lett.* **101**, 240501 (2008).
[67] Kolodynski J. & Demkowicz-Dobrzanski, R. Efficient tools for quantum metrology with uncorrelated noise. *New J. Phys.* **15**, 073043 (2013).
[68] Pirandola, S. & Lupo, C. Ultimate precision of adaptive noise estimation. *Phys. Rev. Lett.* **118**, 100502 (2017).
[69] Pirandola, S. Capacities of repeater-assisted quantum communications. Preprint at https://arxiv.org/abs/1601.00966 (2016).
[70] Demkowicz-Dobrzanski, R. & Maccone, L. Using Entanglement Against Noise in Quantum Metrology. *Phys. Rev. Lett.* **113**, 250801 (2014).
[71] Devetak, I. The private classical capacity and quantum capacity of a quantum channel. *IEEE Trans. Info. Theory* **51**, 44–55 (2005).
[72] Rains, E. M. A semidefinite program for distillable entanglement. *IEEE Trans. Info. Theory* **47**, 2921–2933 (2001).
[73] Audenaert, K., De Moor, B., Vollbrecht, K. G. H. & Werner, R. F. Asymptotic relative entropy of entanglement for orthogonally invariant states. *Physical Review A* **66**, 032310 (2002).
[74] Laurenza, R. & Pirandola, S. General bounds for sender-receiver capacities in multipoint quantum communications. Preprint at https://arxiv.org/abs/1603.07262 (2016).
[75] Takeoka, M., Seshadreesan, K. P. & Wilde, M. M. Unconstrained distillation capacities of a pure-loss bosonic broadcast channel. Preprint at https://arxiv.org/abs/1601.05563v3 (2016).
[76] Ottaviani, C., Laurenza, R., Cope, T. P. W., Spedalieri, G., Braunstein, S. L. & Pirandola, S. Secret key capacity of the thermal-loss channel: Improving the lower bound. *Proc. SPIE 9996, Quantum Information Science and Technology II, 999609* (2016); doi:10.1117/12.2244899. Preprint at https://arxiv.org/abs/1609.02169 (2016).
[77] Pirandola, S., Laurenza, R., Ottaviani, C., Banchi, L. The Ultimate Rate of Quantum Cryptography. Preprint at https://arxiv.org/abs/1510.08863v1 (Version 1, 29 October 2015).
[78] Pirandola, S. & Laurenza, R. General Benchmarks for Quantum Repeaters. Preprint at https://arxiv.org/abs/1512.04945v1 (Version 1, 15 December 2015).
[79] Namiki, R. Teleportation stretching for lossy Gaussian channels. Preprint at https://arxiv.org/abs/1603.05292v1 (2016).
[80] Pirandola, S., Laurenza, R., Ottaviani, C., Banchi, L. The Ultimate Rate of Quantum Communications. Preprint at https://arxiv.org/abs/1510.08863v2 (Version 2, 8 December 2015).
[81] Vaidman, L. Teleportation of quantum states. *Phys. Rev. A* **49**, 1473–1476 (1994).
